# Life After Mild Traumatic Brain Injury: Widespread Structural Brain Changes Associated With Psychological Distress Revealed With Multimodal Magnetic Resonance Imaging

**DOI:** 10.1016/j.bpsgos.2022.03.004

**Published:** 2022-03-16

**Authors:** Francesca Sibilia, Rachel M. Custer, Andrei Irimia, Farshid Sepehrband, Arthur W. Toga, Ryan P. Cabeen, TRACK-TBI Investigators, Opeolu Adeoye, Neeraj Badjatia, Yelena Bodien, M. Ross Bullock, Randall Chesnut, John D. Corrigan, Karen Crawford, Ramon Diaz-Arrastia, Ann-Christine Duhaime, Richard Ellenbogen, V. Ramana Feeser, Adam R. Ferguson, Brandon Foreman, Raquel Gardner, Etienne Gaudette, Dana Goldman, Luis Gonzalez, Shankar Gopinath, Rao Gullapalli, J. Claude Hemphill, Gillian Hotz, Frederick K. Korley, Joel Kramer, Natalie Kreitzer, Chris Lindsell, Joan Machamer, Christopher Madden, Alastair Martin, Thomas McAllister, Randall Merchant, Laura B. Ngwenya, Florence Noel, David Okonkwo, Eva Palacios, Daniel Perl, Ava Puccio, Miri Rabinowitz, Claudia Robertson, Jonathan Rosand, Angelle Sander, Gabriella Satris, David Schnyer, Seth Seabury, Sabrina Taylor, Arthur Toga, Alex Valadka, Mary Vassar, Paul Vespa, Kevin Wang, John K. Yue, Ross Zafonte

**Affiliations:** a Laboratory of Neuro Imaging, Mark and Mary Stevens Neuroimaging and Informatics Institute, Keck School of Medicine, University of Southern California, Los Angeles, California; b Ethel Percy Andrus Gerontology Center, Leonard Davis School of Gerontology, University of Southern California, Los Angeles, California; c Department of Biomedical Engineering, Viterbi School of Engineering, University of Southern California, Los Angeles, California

**Keywords:** Anxiety, Brain vasculature, Depression, Somatization, Structural imaging, Traumatic brain injury

## Abstract

**Background:**

Traumatic brain injury (TBI) can alter brain structure and lead to onset of persistent neuropsychological symptoms. This study investigates the relationship between brain injury and psychological distress after mild TBI using multimodal magnetic resonance imaging.

**Methods:**

A total of 89 patients with mild TBI from the TRACK-TBI (Transforming Research and Clinical Knowledge in Traumatic Brain Injury) pilot study were included. Subscales of the Brief Symptoms Inventory 18 for depression, anxiety, and somatization were used as outcome measures of psychological distress approximately 6 months after the traumatic event. Glasgow Coma Scale scores were used to evaluate recovery. Magnetic resonance imaging data were acquired within 2 weeks after injury. Perivascular spaces (PVSs) were segmented using an enhanced PVS segmentation method, and the volume fraction was calculated for the whole brain and white matter regions. Cortical thickness and gray matter structures volumes were calculated in FreeSurfer; diffusion imaging indices and multifiber tracts were extracted using the Quantitative Imaging Toolkit. The analysis was performed considering age, sex, intracranial volume, educational attainment, and improvement level upon discharge as covariates.

**Results:**

PVS fractions in the posterior cingulate, fusiform, and postcentral areas were found to be associated with somatization symptoms. Depression, anxiety, and somatization symptoms were associated with the cortical thickness of the frontal-opercularis and occipital pole, putamen and amygdala volumes, and corticospinal tract and superior thalamic radiation. Analyses were also performed on the two hemispheres separately to explore lateralization.

**Conclusions:**

This study shows how PVS, cortical, and microstructural changes can predict the onset of depression, anxiety, and somatization symptoms in patients with mild TBI.

Traumatic brain injury (TBI) is defined as a nondegenerative, noncongenital insult to the brain from an external mechanical force. Based on the duration of loss of consciousness and posttraumatic amnesia, TBI can be defined as mild, moderate, or severe, measured via the Glasgow Coma Scale (GCS) (1). A GCS score between 13 and 15 defines mild TBI (mTBI), GCS scores from 9 to 12 indicate moderate TBI, and scores of GCS ≤ 8 indicate severe TBI (2). In mTBI, which represents 70% of all TBI cases (3), loss of consciousness is ∼30 minutes or less and posttraumatic amnesia is <24 hours (4), leading to potential cognitive, physical, and psychological impairments. There is evidence of cognitive and physical postinjury improvement (5,6), while less is known about the psychological consequences in mTBI subacute stages (>3 months). Psychological distress is defined as a “unique discomforting, emotional state experienced by an individual in response to a specific stressor or demand that results in harm, either temporary or permanent, to the person” (7).

The onset of psychological symptoms in mTBI is usually seen 24 to 72 hours after the traumatic event, with improvements within 3 months from injury (8). The risk of developing depression and anxiety can persist after acute stages (9, 10, 11). The onset of psychological symptoms is therefore an important predictor for monitoring recovery within 6 months following TBI (12). Emotional dysregulation following TBI reflects a potential neural injury that can be detected with neuroimaging techniques, such as magnetic resonance imaging (MRI) (12). In recent years, automated neuroimaging analysis tools have been used to study structural alterations in cortical thickness, white matter (WM) microstructure, and vascular components following mTBI (13); combining more MRI approaches has led to more accurate estimations of recovery time in acute stages and in differentiating patients with mTBI from healthy control subjects (14).

Most studies focused on a single type of structural alteration, but it is likely that the onset of psychological distress is caused by simultaneous alterations across multiple brain components. Addressing this issue requires further investigation of multiple distinct brain systems in the same cohort with respect to a wider range of measures of psychological stress and well-being. Neurologic and cognitive alterations in patients with TBI have a remarkable impact on life quality and satisfaction levels after TBI, especially in social and emotional functioning (15), and the onset of psychological distress can be influenced by the level of happiness perceived, as previously shown (16,17). Nevertheless, the association between structural MRI parameters and life satisfaction in patients with TBI is still unclear. Investigating such a relationship is crucial to understanding the underlying neural disruptions that can affect quality of life in patients with mTBI.

The aim of this study is to identify anatomical associations with depressive, anxiety, and somatization symptoms in patients with mTBI 6 months after the traumatic event. We used a multimodal structural neuroimaging approach that provides a combined view of cortical morphometry, WM connectivity, and perivascular spaces (PVSs). We further investigated the relationship between the structural MRI measures and level of life satisfaction within the first 6 months after TBI.

## Methods and Materials

### Participants

Participants (*N* = 89, age range: 16–80 years old) were part of the TRACK-TBI (Transforming Research and Clinical Knowledge in Traumatic Brain Injury) pilot study. TRACK-TBI is a prospective, multicenter observational study conducted at 18 U.S. trauma centers, enrolling patients with TBI between February 26, 2014, and August 8, 2018. Demographic and imaging data and psychological assessment were downloaded from the Federal Interagency Traumatic Brain Injury Research repository (https://fitbir.nih.gov).

Inclusion criteria for the TRACK-TBI pilot were age over 16 years old, acute external force trauma to the head, presentation to an enrolling center, and performance of noncontrast head computed tomography to assess for evidence of acute TBI that meets the American Congress of Rehabilitation Medicine criteria (https://acrm.org/wp-content/uploads/pdf/TBIDef_English_10-10.pdf) within 24 hours of injury. Exclusion criteria were pregnancy, ongoing life-threatening disease (e.g., end-stage malignancy), police custody, involuntary psychiatric hold, non-English speaker (because multiple outcome measures were administered and/or normed only in English), and GCS score < 13, because this was a study focused on mTBI. Participants were mostly White (74%) and right-handed (94%). All study protocols were approved by the University of California at San Francisco Institutional Review Board, and all patients and control subjects or their legal representatives gave written informed consent.

### Cognitive Assessment

Demographic information, clinical measures, and scores of psychological distress subgroups were collected for each participant. GCS is used to rate consciousness levels following TBI. The total score is the result of three components (verbal, motor, and eye responses), which determine the severity of the traumatic event. It is usually used in the field upon emergency department arrival and at the time of discharge (18). In this study, GCS scores between 13 and 15 were used to determine the level of improvement for each participant, calculated via the difference in discharge and emergency department GCS scores.

#### Brief Symptom Inventory 18

The Brief Symptom Inventory 18 (BSI-18) quantifies psychological distress and comorbidities in patients with mental and somatic illnesses (19). The BSI-18 is the shortened version of the BSI (20). It is reduced to three subscales (anxiety, depression, and somatization) formed by six items each and the Global Severity Index, to improve dimensionality structure and be more accessible to patients (21). Scores for each item are summed up across participants to obtain the total scores for anxiety, depression, and somatization and then converted to T scores. The item list for each subscale is found in Table S1. The Global Severity Index total score ranges between 0 and 72, and the three scale scores range between 0 and 24. The BSI-18 manual suggests respondents with a total Global Severity Index T score > 63 as having significant psychological distress (22). BSI scores considered in this study were administered 6 months after the traumatic injury.

#### Life Satisfaction Measurement

Life satisfaction is defined as “a global assessment of a person’s quality of life according to his chosen criteria” (23), based on a cognitive and judgmental process rather than emotional evaluation (24). The Satisfaction With Life Scale is a 5-item questionnaire where participants are asked to rate the level of satisfaction and conditions of their lives as whole. Outcomes are categorized as “slightly dissatisfied/satisfied,” “dissatisfied/satisfied,” and “extremely dissatisfied/satisfied.” A score of 20 represents the neutral point on the scale (25).

### MRI Data Acquisition

3T MRI sequences were obtained in a SIGNA EXCITE scanner with an eight-channel coil. Axial three-dimensional inversion recovery fast spoiled gradient recalled echo T1-weighted images were acquired with echo time = 1.5 ms; repetition time = 6.3 ms; inversion time = 400 m; flip angle, 15°; 230-mm field of view; 156 contiguous partitions of 1 mm; and matrix size 256 × 256 (26).

Diffusion-weighted imaging (DWI) data were acquired with a multislice single-shot spin echo echo-planar pulse sequence (echo time = 63 ms; repetition time = 14 s) using 55 diffusion-encoding directions, acquired at *b* = 1000 s/mm^2^, 7 acquisitions at *b* = 0 s/mm^2^, 72 interleaved slices of 1.8-mm thickness and no gap between slices, a 128 × 128 matrix, and a field of view of 230 × 230 mm.

We implemented a multimodal image analysis pipeline that included components for WM analysis of fiber bundles, gray matter analysis of cortical thickness and subcortical volume, and PVS analysis to detect abnormal PVS enlargement in the brain vasculature. The steps of the pipeline are described as follows.

### Diffusion MRI Preprocessing

Standard DWI processing was performed using a combination of the FSL Diffusion Toolbox (27), the Advanced Normalization Tools (https://stnava.github.io/ANTs/) (28), and the Quantitative Imaging Toolkit (QIT) (http://cabeen.io/qitwiki) (29). We first applied a nonlocal means filter to the DWI data using the VolumeFilterNLM module in QIT (30), and then *FSL-eddy* was used to correct for eddy current-induced distortions (31). An automated quality control step was performed to quantify the DWI data quality using *FSL-eddy_quad* (32) at a subject level. Brain segmentation was performed using FSL BET (33), and diffusion tensor imaging (DTI) parameters were estimated using weighted-linear least squares estimation (34) implemented in the VolumeTensorFit module of QIT. Parameter maps for DTI parameters were extracted and retained for quantitative analysis (35), including fractional anisotropy (FA), mean diffusivity (MD), radial diffusivity, and axial diffusivity. For tractography analysis, ball-and-sticks modeling was performed using the Monte Carlo Markov chain approach in FSL BEDPOSTX (36).

### WM Analysis

We performed quantitative tractography analysis to characterize the microstructure of 33 fiber bundles of interest (including association, commissural, projection, and cerebellar pathways), with separate models for the left and right hemispheres. A list of the bundles of interest considered is found in Supplemental Methods. We extracted fiber bundle models from each individual case using an atlas-based bundle-specific approach. We followed a similar approach to past work (37) to create a priori bundle definitions with group-averaged multifiber models (38) in the IIT ICBM diffusion MRI template space (39). We manually delineated bundle seed, inclusion, exclusion, and stopping volumetric masks for each bundle of interest based on anatomical references (40,41). We performed diffeomorphic registration of the subject FA maps to the IIT template FA maps using Advanced Normalization Tools (42), and the resulting deformations were applied to transform each bundle delineation mask into subject native space. We then applied a hybrid reinforcement tractography approach (43) to obtain subject-specific fiber bundles using the ball-and-sticks voxelwise models with the following parameters: a maximum turning angle of 75°, a minimum volume fraction of 0.05, trilinear interpolation, a step size of 1 mm, and a minimum length of 10 mm. We measured bundle-specific DTI parameters by sampling the voxelwise parameter values at each curve vertex and computing the average across each entire bundle to obtain DTI metrics for statistical analysis. Figure S1 shows an example of the segmentation performance of two WM tracts on two individuals. We also summarized each fiber bundle with its morphometric properties, e.g., mean tract density, volume, mean length, and mean bundle thickness. For each fiber bundle parameter, the average of the left and right were computed; this was considered primarily, and lateralization was investigated secondarily.

### Gray Matter Analysis

Cortical surface analysis was conducted using FreeSurfer version 5.3.0 (44). Regional averages of cortical thickness were computed using the T1-weighted MRI of each subject using the Desikan-Killiany cortical atlas (45). Subcortical volumes were computed using the *aparc* output of the FreeSurfer pipeline, which included the caudate, putamen, amygdala, thalamus, and hippocampus.

### Perivascular Space Analysis

PVS was segmented using a previously published technique developed in our institute (46), based on an enhanced PVS contrast. After data acquisition and preprocessing, T1- and T2-weighted images were filtered by using nonlocal mean filtering. Nonlocal mean technique measures the image intensity similarities by taking into account the neighboring voxels in a blockwise fashion, where filtered image is ∑_xi∈Viω_*(xi,xj)u*(*xj*). For each voxel (*x*_*j*_), the weight (*ω*) is measured using the Euclidean distance between three-dimensional patches. The adaptive nonlocal mean filtering technique adds a regularization term to the above formulation to remove bias intensity of the Rician noise observed in MRI and estimate the filter bandwidth in each voxel. This allows for removal of high-frequency spatial noise at a single-voxel level and preservation of signal intensities of PVS voxels. Enhanced PVS contrast was obtained by dividing filtered T1w and T2w images.

MRI images were parcellated using Advanced Normalization Tools (28) to obtain masks of WM and basal ganglia. Parcellated WM was used as a mask for PVS analysis. For PVS segmentation, a Frangi filter was applied using QIT (29), which extracts the likelihood of a voxel belonging to a PVS. Frangi filter estimated vesselness measures at different scales and provided the maximum likeliness. The scale was set to a large range of 0.1 to 5 voxels to maximize the vessel inclusion. The output of this step was a quantitative maximum likelihood map of vessels in regions of interest. The outputs across voxels comprise vesselness measured across a range of filter scales. A threshold was applied to the vesselness map to obtain a binary mask of PVS regions. We chose a previously optimized scaled threshold of 1.5 (equal to raw threshold of 1 × 10^−6^), which was required to calculate PVS volumetric measurements and spatial distribution.

### Statistical Analysis

Statistical analysis was performed in R version 4.0.5. Multivariable linear regressions were performed to investigate the MRI-related parameters predicting development of psychological distress 6 months following TBI. A DTI-based white matter FA map from the JHU atlas (47, 48, 49), gray matter subcortical areas maps, whole-brain WM volume and cortical thickness maps from FreeSurfer, and global and region-based PVS fractions were considered in the analysis. Diffusivity measures (FA, MD, radial diffusivity, and axial diffusivity) and geometric tract properties (tract density, volume, length, and thickness, calculated both for the entire bundle and for each tract divided into head, middle, and tail) were also included. The complete list of all the variables used for the analysis can be found in section 3 of the Supplement.

MRI parameters presenting more than 60% of missing data across participants were removed from the analysis; in our case, the anterior commissure and the first branch of the superior longitudinal fasciculus were excluded. Outliers were removed using Tukey’s method, considering twice the interquartile range. MRI parameters were converted into *z* scores to ensure regression coefficients to be reported as standardized effect sizes.

BSI outcome scores for anxiety, depression, and somatization were used as independent variables, controlled for age, biological sex, educational attainment (in years), total intracranial volume, and improvement levels. Sex was converted into a categorical variable. Covariate missing values treatment was based on mean imputation procedure. Each imaging parameter was considered as dependent variable in the regression model, with an α level of 0.05 (614 variables in total; a complete list may be found in Table S6).

Uncorrected significant *p* values underwent correction for multiple comparison, using Benjamini-Hochberg false discovery rate (FDR) approach (50), to obtain *q* values. FDR correction was performed across imaging modalities for each symptom (depression, anxiety, somatization). Adjusted *R*^2^ and *β* regression coefficient values were also computed and reported in the Supplement.

## Results

Demographic summaries are shown in Table 1. The population was formed by 60 males and 29 females, with a mean age of 37.03 ± 15.06 years, and a median value of 34. Distributions of the age across the entire population and divided into two subgroups (based on median value) are represented in Figure S2. There were 47 individuals (52.8%) between 16 and 34 years old, and 42 participants who were 35 years old or older (47.2%). Demographic information based on sex is shown in Table 2. In this section, results are reported considering both hemispheres together. To test lateralization effect, analyses were also run on each hemisphere separately (see the Supplement for results).

**Table 1:** Demographic Information and Summary Statistics of Psychological Distress Variables and TBI-Related Scores in the Population Considered in This Study

Variable	*N*	Mean (SD)	Min	Q1	Median	Q3	Max
Age	89	37.03 (15.06)	16.00	25.00	34.00	50.00	80.00
GCS Field	89	14.26 (1.68)	4.00	14.00	15.00	15.00	15.00
GCS Discharge	89	14.89 (0.38)	13.00	15.00	15.00	15.00	15.00
GCS ED	89	14.62 (0.67)	11.00	14.00	15.00	15.00	15.00
Rivermead^a^	89	15.78 (15.54)	0.00	2.00	12.00	26.00	59.00
Education	89	14.67 (2.71)	6.00	13.00	14.67	16.00	22.00
Depression	89	56.52 (11.81)	40.00	45.00	58.00	66.00	81.00
Anxiety	89	56.22 (12.19)	38.00	47.00	59.00	67.00	81.00
GSI	89	57.45 (12.09)	33.00	48.00	59.00	67.00	81.00
Somatization	89	55.97 (11.40)	41.00	48.00	56.00	64.00	81.00
SWLS	89	19.59 (7.86)	5.00	14.00	20.00	25.00	35.00

Depression, anxiety, GSI, and somatization are considered in T score.

GCS, Glasgow Coma Scale; ED, emergency department; GSI, Global Severity Index; Max, maximum; Min, minimum; Q1, quartile 1; Q3, quartile 3; SWLS, Satisfaction With Life Scale; TBI, traumatic brain injury.

a Rivermead is a questionnaire to monitor post-TBI symptoms, and the total score indicates the severity of the TBI symptoms.

**Table 2:** Demographic Information of the Population Based on Biological Sex After Dummy Coding

Variable	Sex	*n*	Mean (SD)	Min	Q1	Median	Q3	Max
Age	1	60	38.10 (14.73)	16.00	25.00	37.00	50.00	80.00
	2	29	34.83 (15.76)	16.00	23.00	31.00	48.00	73.00
GCS Field	1	60	14.10 (1.96)	4.00	14.00	15.00	15.00	15.00
	2	29	14.59 (0.73)	12.00	14.00	15.00	15.00	15.00
GCS Discharge	1	60	14.92 (0.33)	13.00	15.00	15.00	15.00	15.00
	2	29	14.83 (0.47)	13.00	15.00	15.00	15.00	15.00
GCS ED	1	60	14.57 (0.70)	11.00	14.00	15.00	15.00	15.00
	2	29	14.72 (0.59)	13.00	15.00	15.00	15.00	15.00
Rivermead^a^	1	60	16.18 (15.71)	0.00	2.00	12.50	26.50	58.00
	2	29	14.97 (15.44)	0.00	2.00	9.00	25.00	59.00
Education	1	60	14.70 (2.65)	8.00	13.00	14.67	16.00	22.00
	2	29	14.61 (2.87)	6.00	13.00	15.00	16.00	20.00
Depression	1	60	58.52 (12.10)	42.00	48.00	60.00	67.00	81.00
	2	29	52.38 (10.16)	40.00	45.00	50.00	62.00	70.00
Anxiety	1	60	56.72 (12.55)	39.00	47.50	60.00	68.00	81.00
	2	29	55.21 (11.57)	38.00	45.00	57.00	65.00	74.00
GSI	1	60	59.10 (11.79)	36.00	50.00	61.00	68.50	81.00
	2	29	54.03 (12.20)	33.00	45.00	57.00	61.00	79.00
Somatization	1	60	56.88 (11.53)	41.00	48.00	56.00	65.00	81.00
	2	29	54.07 (11.10)	41.00	42.00	50.00	63.00	77.00
SWLS	1	60	19.28 (8.18)	5.00	12.00	21.50	25.00	33.00
	2	29	20.23 (7.23)	5.00	18.00	19.00	26.00	35.00

Dummy coding: 1 = male and 2 = female. Depression, anxiety, GSI, and somatization are considered in T the score.

GCS, Glasgow Coma Scale; ED, emergency department; GSI, Global Severity Index; Max, maximum; Min, minimum; Q1, quartile 1; Q3, quartile 3; SWLS, Satisfaction With Life Scale.

a Rivermead is a questionnaire to monitor post-TBI symptoms, and the total score indicates the severity of the TBI symptoms.

### Brief Symptom Inventory-18

#### Anxiety

**Figure 1:**
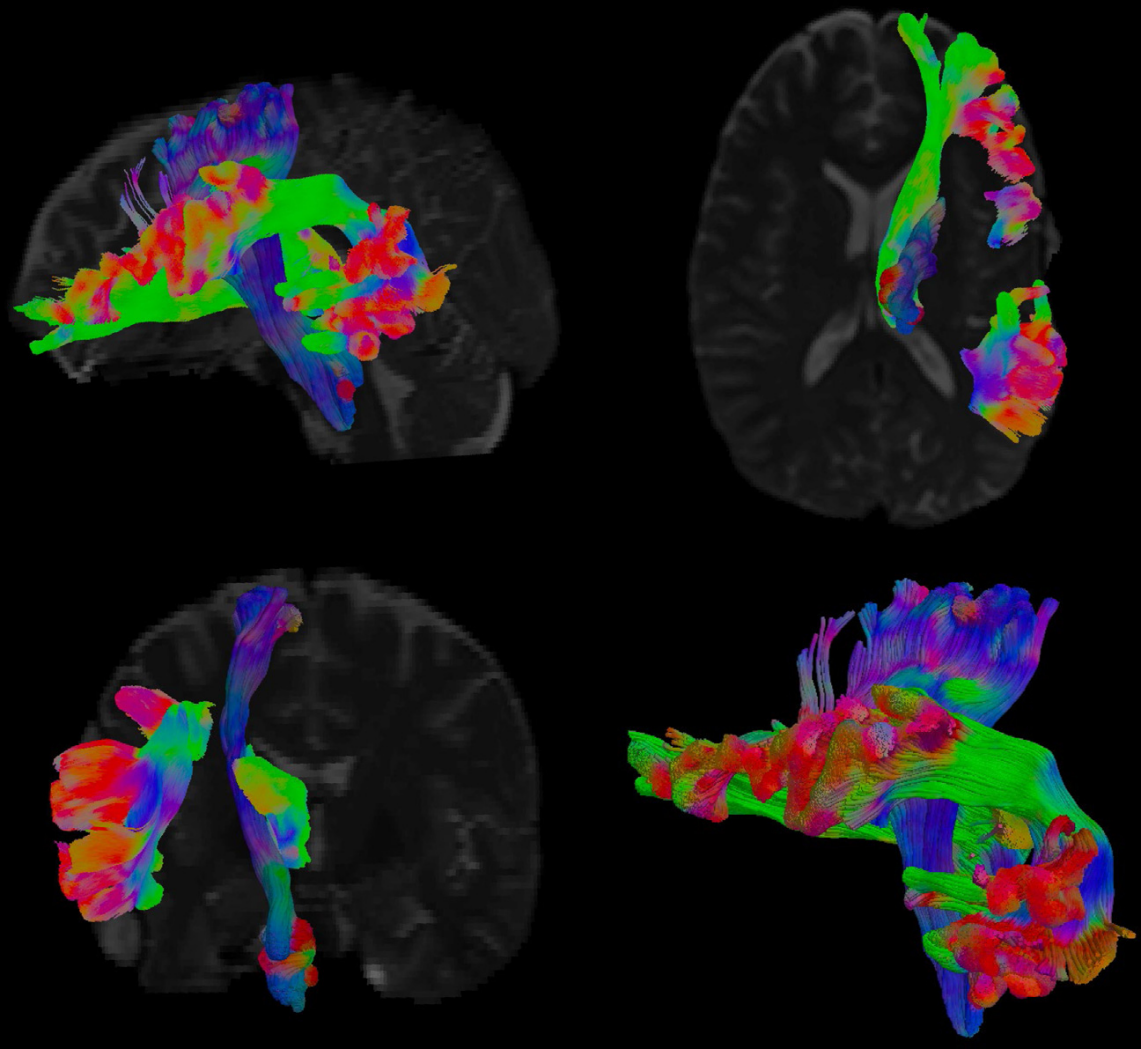
Graphical representation of white matter tracts that showed geometric features (thickness and length) significantly associated to somatization symptoms. The tract in blue (the anterior thalamic radiation) was found to be associated to both somatization and anxiety traits.

**Figure 2:**
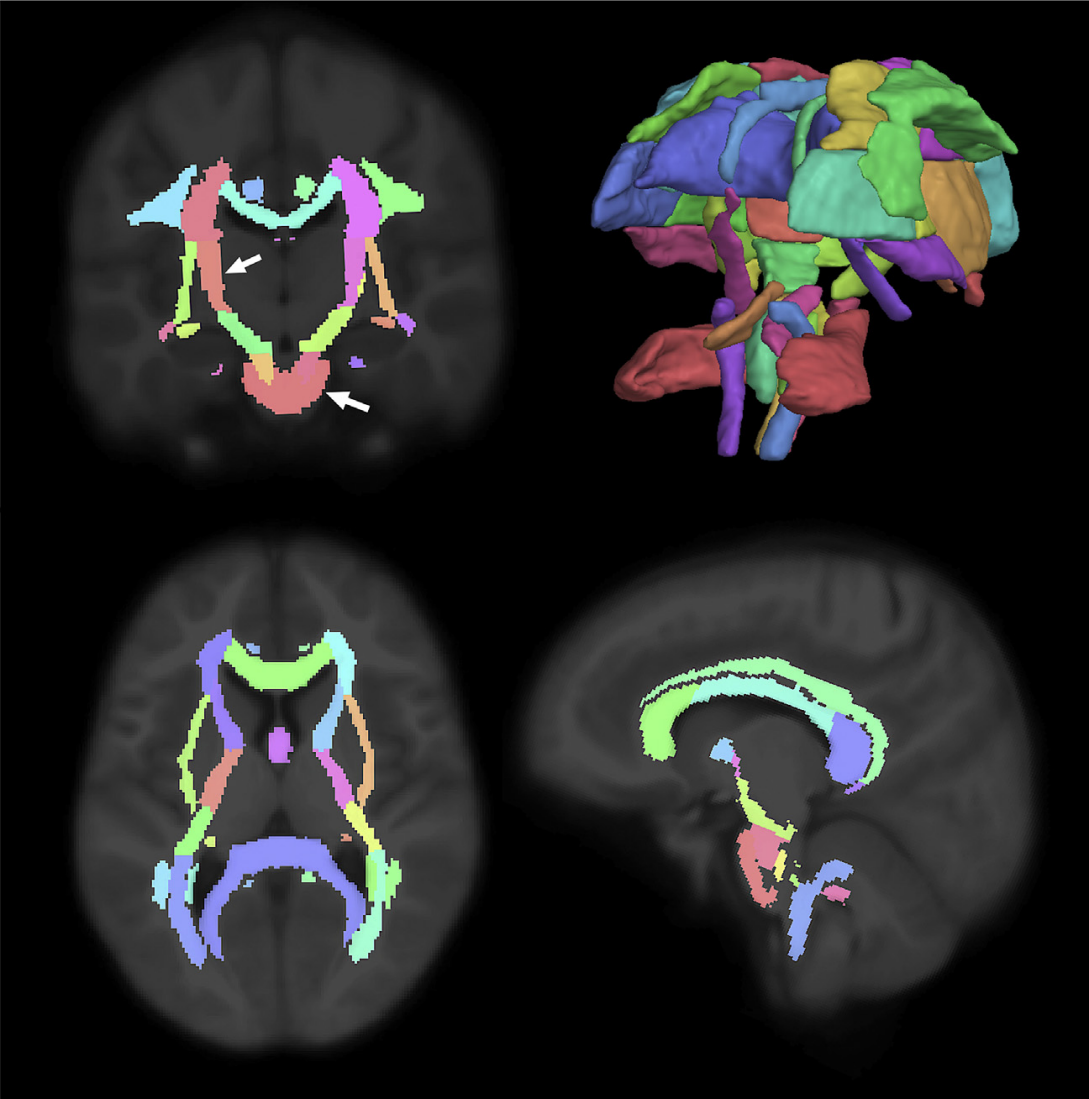
John Hopkins atlas (41) of white matter regions. The cerebellar peduncle and internal capsule (both in salmon color, see arrows on the coronal view) were the common regions that showed a significant relationship between fractional anisotropy and the three domains of psychological distress (depression, anxiety, and somatization; *p* < .05 false discovery rate–corrected).

After FDR correction, a significant negative relationship was seen between anxiety levels across the follow-up period and the cortical thickness of the inferior-opercular frontal gyrus and occipital pole, and the putamen subcortically. A significant positive relationship was seen with tract thickness of the anterior thalamic radiation (Figure 1, shown in blue), as well as with the FA of the cerebral peduncle, posterior limb of internal capsule and external capsule, and superior longitudinal fasciculus from the JHU WM atlas (regions of interest are indicated in Figure 2). Table S2 lists the brain regions significantly associated with anxiety symptoms, and Figure S2 shows the correspondent scatterplots.

#### Depression

**Figure 3:**
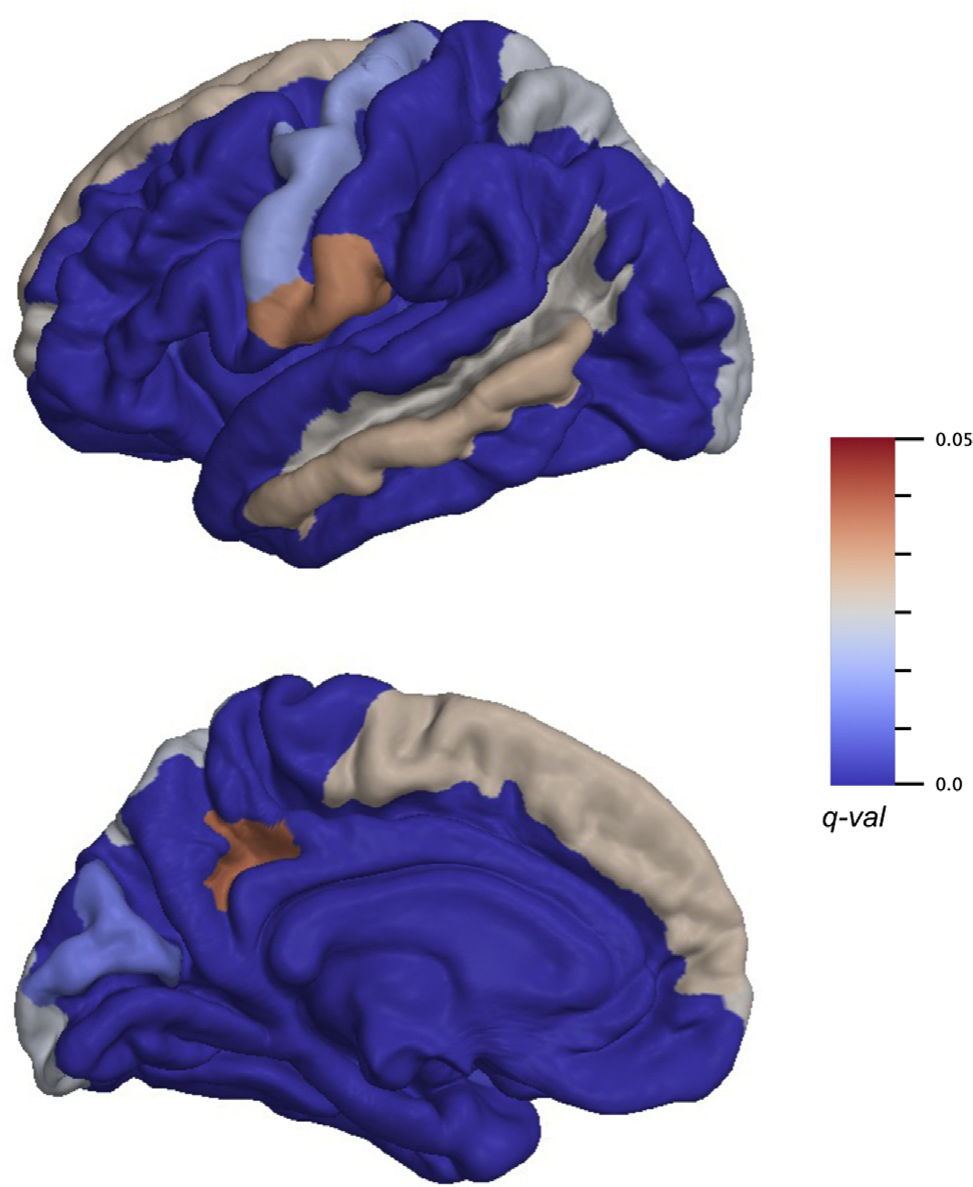
Brain regions in which cortical thickness is significantly associated to depression symptoms. Color bar indicates *q* values. Because results consider the two hemispheres together, only the left hemisphere is shown for visualization purposes.

Depression was negatively associated to widely distributed cortical thickness changes, specifically in the subcentral, frontopolar sulci and gyri, dorsal posterior cingulate, cuneus, inferior frontal opercular, frontosuperior, insular, inferior supramarginal and superior parietal gyrus, precentral gyrus, middle temporal gyrus, occipital pole, circular insular sulci, medial orbito-olfactory sulcus, and superior temporal sulcus (Figure 3). FA was a significant predictor of psychological distress for the cerebral peduncle (*q* = .03) and posterior limb of the internal capsule (*q* < .001). The complete list of the significant predictors can be found in Table S3 with the respective FDR-corrected *p* values, and Figure S3 shows the correspondent scatterplots.

#### Somatization

**Figure 4:**
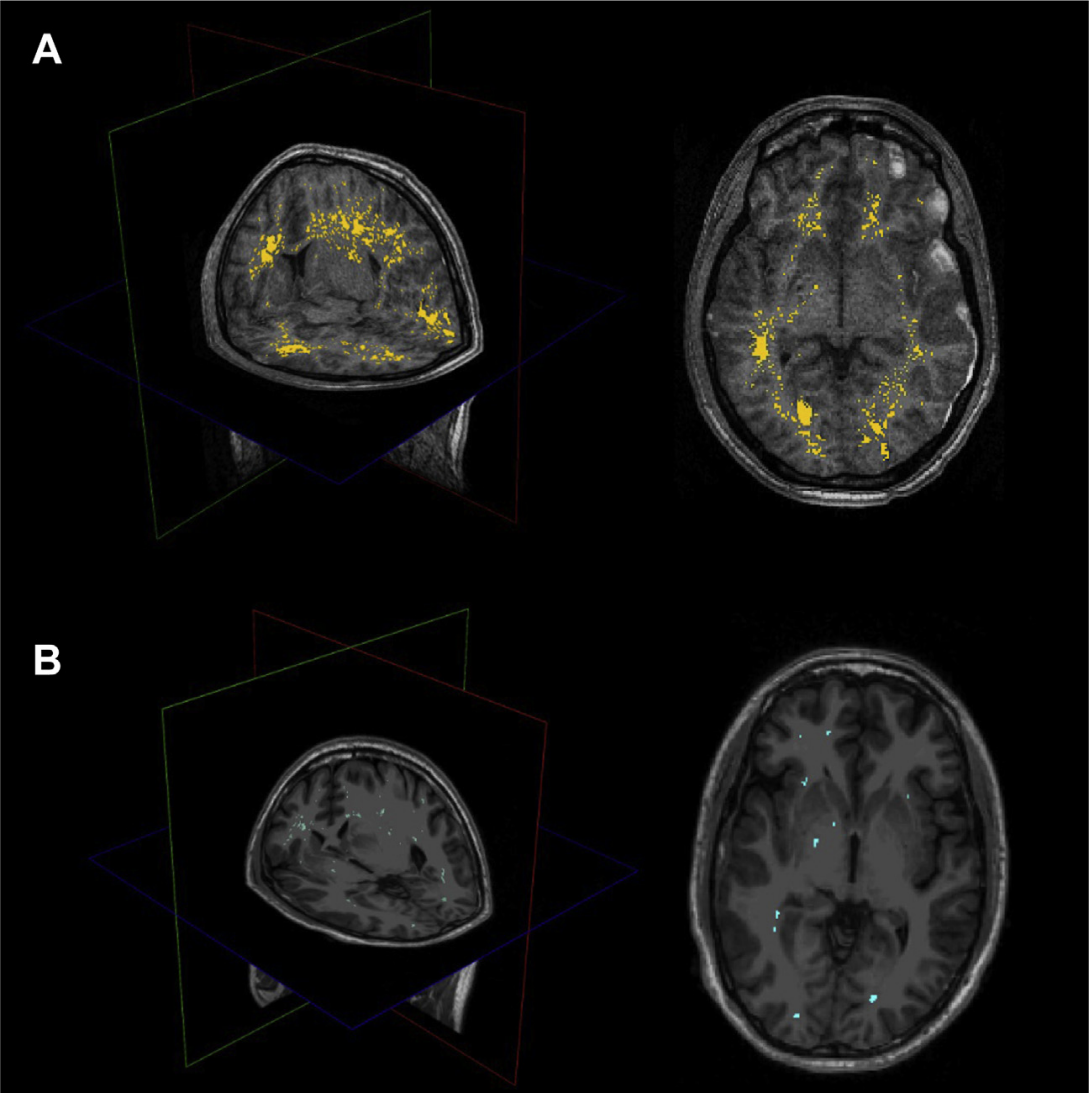
Perivascular space map on two different subjects with high **(A)** and low **(B)** perivascular spaces count, shown on three-dimensional plane and axial view. Somatization symptoms were found to be significantly associated with global perivascular space volume in white matter, as well as locally in the posterior cingulate, fusiform area, and postcentral areas.

**Figure 5:**
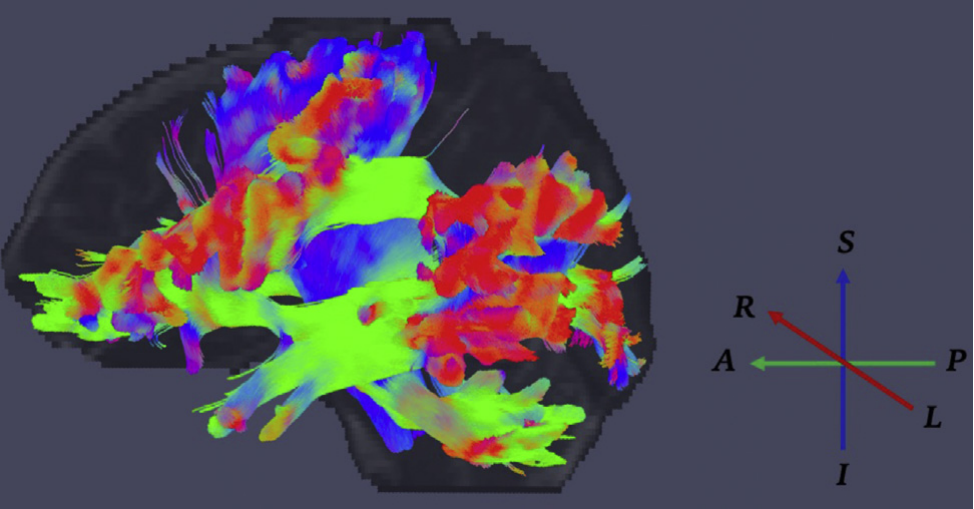
Visual representation of white matter tracts that were significantly different in diffusivity metrics (mean and radial diffusivity) when considering somatization symptoms. Arrows indicate the RGB color system of diffusion directions. A, anterior; I, inferior; L, left; P, posterior; R, right; S, superior.

**Figure 6:**
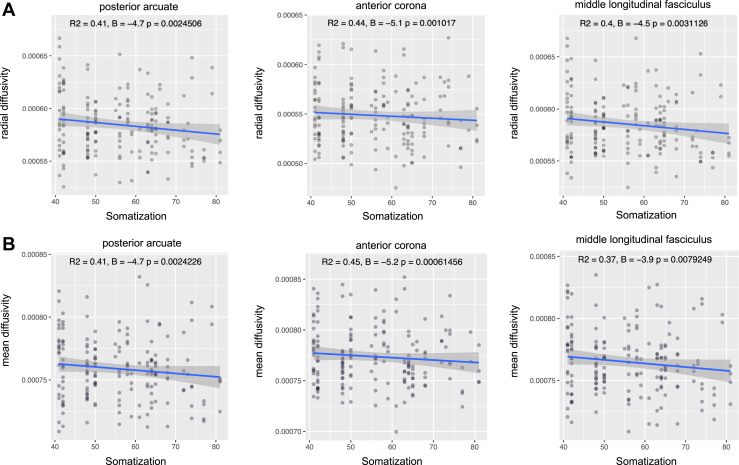
Scatterplots indicating a significant negative relationship of radial diffusivity **(A)** and mean diffusivity **(B)** with somatization symptoms in the posterior arcuate, anterior corona, and middle longitudinal fasciculus. *R*^2^, *β*, and *p* values are also indicated.

Putamen (*q* = .02), amygdala, hippocampus, and thalamus volumes were associated with the somatization trait in patients with mTBI (all *q* < .005). As for the WM volume, significant associations were seen in the fusiform, enthorinal, inferiortemporal, parahippocampal, precentral, paracentral, postcentral, posterior cingulate, supramarginal and superior-frontal areas (*q* < .05). A significant association was found with the perivascular component in the fusiform, postcentral, and posterior cingulate areas, and overall, WM volume (Figure 4). As for the tract morphological properties, the volume and length of the corticospinal tract and the anterior thalamic radiation were predictors of somatization (*q* < .05) (Figures 1 and 5). A significant negative relationship was found with the MD of the uncinate fasciculus, inferior-fronto-orbital fasciculus, corticospinal tract, posterior arcuate, superior cerebellar tract, second branch of superior longitudinal fasciculus, frontoanterior thalamic tract, superior thalamic radiation, middle longitudinal fasciculus, arcuate fasciculus, and anterior corona. For the last three tracts, a negative association was also seen with radial diffusivity (Figures 5 and 6). Scatterplots of all statistically significant associations are shown in the Supplement (Figure S4).

### Life Satisfaction

No significant associations were found after correction for multiple comparisons. Uncorrected *p* values, *R*^2^, and *β* values of all the brain regions predicting life satisfactory scores are reported in the Supplement (Table S4 shows the two hemispheres together; in Table S5 results are reported for the two hemispheres separately).

## Discussion

This study analyzed the relationship between structural imaging biomarkers and the presence of depression, anxiety and somatization symptoms 6 months after mild TBI events. Findings showed that the cortical thickness, diffusivity properties, and perivascular components of brain regions predicting psychological distress are mostly involved in negative affective bias, emotional recall of memories, and sensory-motor functions.

### Somatization

A relationship between PVSs and somatization was found in the fusiform, postcentral, and posterior cingulate areas. This is the first time that PVS abnormalities have been reported to be predictor of somatization symptoms in patients with mTBI. At a physiological level, the cognitive problems seen in patients with mTBI, especially mnemonic and emotional domain, are to be associated to alteration of the glymphatic system functionality (51); we would expect accompanying changes in the PVSs, because they play a crucial role in the brain clearance activity. PVS enlargements result in a decreased flow of soluble waste, promoting the accumulation of neurotoxic molecules, such as amyloid-β. Somatization is defined as the expression of mental distress as physical symptoms (52). Lifestyle choices and physiological factors were previously found to trigger PVS structural alteration: for instance, higher body mass index potentially causes an increase of somatization symptoms severity (53) and PVS enlargement in WM (54). Likewise, poor quality of sleep and insomnia are important factors mediating such relationships, because just one night of deprived sleep has been shown to affect significantly PVS structure and function (55,56). Future studies will help understand the deeper relationship between the alteration of PVS anatomy and the onset of psychological distress symptoms.

The GM and WM regions found to be significant predictors were previously shown to be structurally altered in first episode, drug-naïve patients with somatization disorder (57), reflecting changes in resting-state connectivity in the corticolimbic-cerebellar circuit in patients with somatization disorder. The fusiform gyrus is part of the fusiform-amygdala circuit, and it is involved in social anxiety, avoidance, and sensitivity to punishment (58). The posterior cingulate and postcentral areas are key regions involved in basic emotion regulation processing, being part of the frontolimbic and social networks (59). Several studies have reported structural volume decreases in these areas in patients with somatization symptoms, related to higher scores of somatic symptoms (60,61).

Results showed an association between increased amygdala thickness and psychological distress symptoms after TBI. This might suggest a link between amygdala structural changes and behavioral alterations related to the emotional domain in patients with TBI, as seen in previous findings (62).

### Depression

Depressive symptoms are among the most frequent psychiatric conditions after mTBI (63), with a range between 10% and 77% among patients. Depression after mild TBI contributes to the exacerbation of postconcussive symptoms, such as dizziness, headache, sleep disorders, and increases in aggressive behavior and suicidal thoughts (63). Neurochemical disruptions underlie post-TBI depressive symptoms, with chronic cholinergic deficits and dysfunction in dopaminergic, noradrenergic, and serotonergic systems all contributing (64). Neurophysiological dysfunction and the severity of symptoms varies among patients and is influenced by the patient’s medical history (such as mood disorders, alcohol, or drug abuse), as well as by postinjury factors (for instance, lack of social support or frustration in dealing with physical complications).

The internal capsule was found to be a significant predictor of both anxiety and depression symptoms in people with mTBI. The first study about diffuse axonal injury on patients with mTBI (65) showed FA changes in the internal capsule, external capsule, and corpus callosum immediately after the traumatic event; our results suggest that such changes can persist overtime. The diffusivity metric alterations underlie diffuse axonal injury, a form of TBI characterized by axon shearing at the junction of GM and WM, leading to microstructural damage of fiber tract (66) and changes in water molecule diffusion along the axons. In a group of patients with mTBI following motor vehicle accident, diffusivity alterations were associated with worse processing speed and working memory indices (67), giving more insight on the neurobiological disruptions that lead to cognitive impairment caused by brain injuries. The internal capsule is one of the more commonly reported predictors of postinjury depression, together with the corpus callosum, anterior and posterior corona radiata, anterior and posterior thalamic radiations, and corticospinal tract (68). In our study, FA decreases and MD increases of these WM tracts were associated with anxiety, depression, and somatization symptoms altogether. Such regions are part of circuits involved in sleep-wake regulation, information processing, attention, executive function, and emotion regulation, all of which are shown to be impaired after mTBI.

### Anxiety

Post-TBI anxiety disorder has a prevalence rate of up to 70% (69), and it can manifest in different forms across patients (i.e., anxiety symptoms, posttraumatic stress disorder, obsessive-compulsive disorder, panic, and social anxiety disorder). Previous studies have found associations between anxiety and brain areas of the frontolimbic and corticostriatal pathways, such as the hippocampus, dorsolateral prefrontal and orbital frontal cortices, amygdala, and basal ganglia (70, 71, 72).

### Postinjury Recovery

In this study, we considered the relationship between MRI-based biomarkers and the onset of psychological distress after 6 months from injury. In this time frame, the brain is subject to high plasticity levels that influence recovery stages. Depending on the injury severity and postaccident therapy, the brain can recover its functionality, thereby avoiding permanent impairment of cognitive abilities. A few studies showed a cognitive recovery within the first 6 months (5,73,74), whereas another study suggested that full or partial recovery will not always occur within such a period of time (75). Recovery time varies highly among people, influenced by demographic and lifestyle factors such as age, educational level, and previous medical issues (75, 76, 77). This highlights how structural alterations at 6 months following TBI need to be considered as a candidate biomarker and target for therapeutic interventions.

The findings of this study provide useful information regarding the anatomical areas targeted in mTBI. To localize the effects on psychological symptoms, we considered bilateral measures (obtained by combining the two hemispheres together) as well as hemispherical lateralization. The lateralized brain measures that were significantly associated with psychological distress symptoms confirm findings of a previous study looking at changes of brain connectivity in patients with TBI (78). They showed alterations in structural connectivity in the left thalamus, where we found significant relationships with somatization, and in the right postcentral gyrus, significantly associated with somatization symptoms when considering PVS fraction. This suggests that changes in the different structural compartments and network reorganization following TBI may exacerbate somatization symptoms, because these brain areas are involved in sensation and limbic functions.

To our knowledge, no prior study has yet investigated the association of PVS and psychological distress in patients with mTBI. An alteration in the PVS structure can lead to disruption in the blood-brain barrier (BBB) and alteration in the cerebrovascular physiological flow, promoting deposits of neurotoxic molecules.

Anatomically, PVS enlargements can lead to failure in the BBB and the vascular components supplying that region, namely the anterior and middle cerebral arteries (79). A recent paper quantified BBB permeability increase in animal models with repeated mild head injuries by using dynamic contrast enhanced MRI. They found that at first head impact, BBB permeability increased particularly in the somatosensory and frontal cortices (80). The alteration in BBB integrity caused by brain injury can lead to long-term inflammatory states and culminate in permanent changes in brain homeostasis (81).

### Limitations

In this study, we investigated the relationship of psychological distress symptoms and structural imaging biomarkers cross-sectionally. We were able to collect BSI information at 6 months only and not further time points. A longitudinal component beyond 6 months could be helpful in monitoring symptom severity after the acute and semiacute postinjury stages and infer on the recovery speed. The cross-sectional approach to the analysis also explains the relatively small sample size, represented by participants who had completed the demographic information and self-reported psychological measures in the TRACK-TBI cohort and considering only MRI and DWI with the best quality data. Another potential limitation is considering only structural MRI and no other neuroimaging techniques. Even if we present an exhaustive analysis on the diffusion, volumetric, and perivascular components that predict psychological consequences in patients with TBI, a multimodal approach can help in further understanding the variability in the severity of psychological and cognitive outcome among patients with TBI. It is worth pointing out that the diffusion measures and the tract geometric features computed for the analysis have limited reproducibility across fiber bundle segmentation approaches from different research groups, as shown by a recent study (82). The high variability in WM tract reconstruction influences metrics quantification and, therefore, is an important factor to consider with the interpretation of microstructural changes in clinical populations in conditions when data are pooled across studies. Finally, FreeSurfer version 5.3 was chosen over a newer version to be consistent with that used in the Human Connectome Project pipeline, because the analyses focused on more stable measures of FreeSurfer, rather than more recent segmentation functions.

### Conclusions

Our findings not only confirm previous results on the volumetric and diffusion-based associations to depressive and anxiety symptoms but reveal a further significant relationship between PVS anatomy and somatization disorder. This possibly reflects abnormalities in the brain glymphatic system and BBB integrity, which play a crucial role in brain waste clearance. Alterations of these physiological processes can trigger a cascade of events responsible for neurologic disorder development and cognitive decline (83). Results confirm that the brain is affected by mTBI episodes on different structural compartments, leading to accelerated neurodegenerative and neuroinflammatory processes, if not monitored over time. The role of efficient therapeutic interventions is therefore crucial to prevent the exacerbation of the psychological symptoms and cognitive decline seen already at mild stages of brain injuries, and the effects reported here may provide a possible avenue for treatment evaluation in future studies.

## Supplementary Material

Supplementary Material

Key Resources Table
