# Supplementary material for: Life After Mild Traumatic Brain Injury: Widespread Structural Brain Changes Associated With Psychological Distress Revealed With Multimodal Magnetic Resonance Imaging
Source: Biol Psychiatry Glob Open Sci. 2022 Mar 16;3(3):374–85. doi: 10.1016/j.bpsgos.2022.03.004 (PMC10382710; doi:10.1016/j.bpsgos.2022.03.004)
Supplement: Supplementary Material [file mmc1.pdf]

Supplementary Information

Life After Mild Traumatic Brain Injury: Widespread Structural Brain Changes Associated With Psychological Distress Revealed With Multimodal Magnetic Resonance Imaging

1. Supplementary Methods

BSI-18	Subscale	Item
Global Severity Index (GSI)	Anxiety	Fearful scale
		Restless scale
		Tense scale
		Suddenly scared
		Terror or panic scale
		Nervousness scale
	Depression	Feeling blue scale
		No interest scale
		Thoughts of ending life
		Feeling worthless
		Hopeless scale
		Felling lonely
	Somatization	Chest pain
		Faintness scale
		Feeling weak scale
		Trouble getting breath
		Nausea scale
		Numbness or tingling

Table S1: List of the items composing the three subscales of BSI-18 used for the analysis.

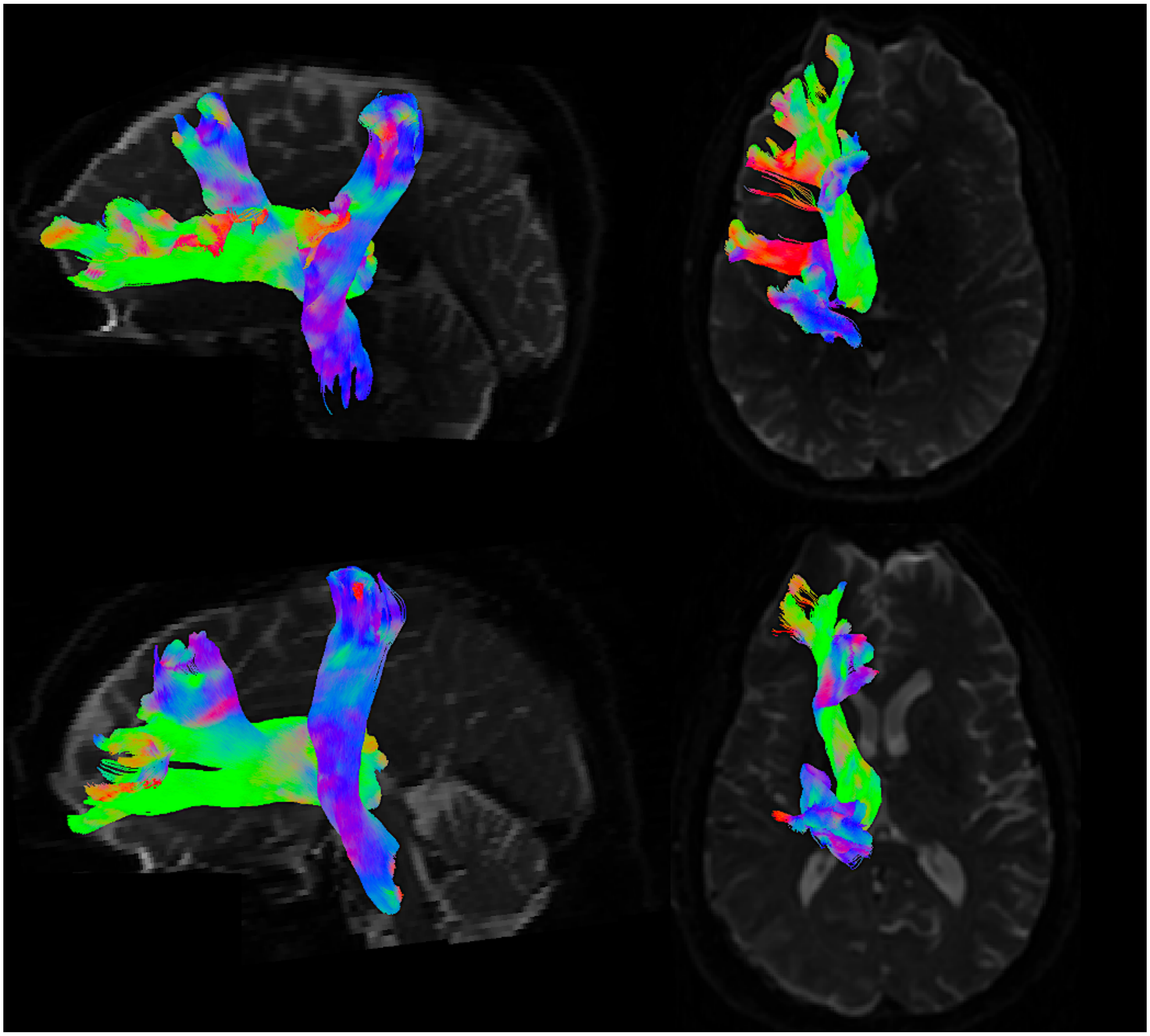

Figure S1: Visual representation of the segmentation performance on the anterior thalamic radiation and corticospinal tract in a young subject (19 years old, top) and an older subject (59 years old, bottom).

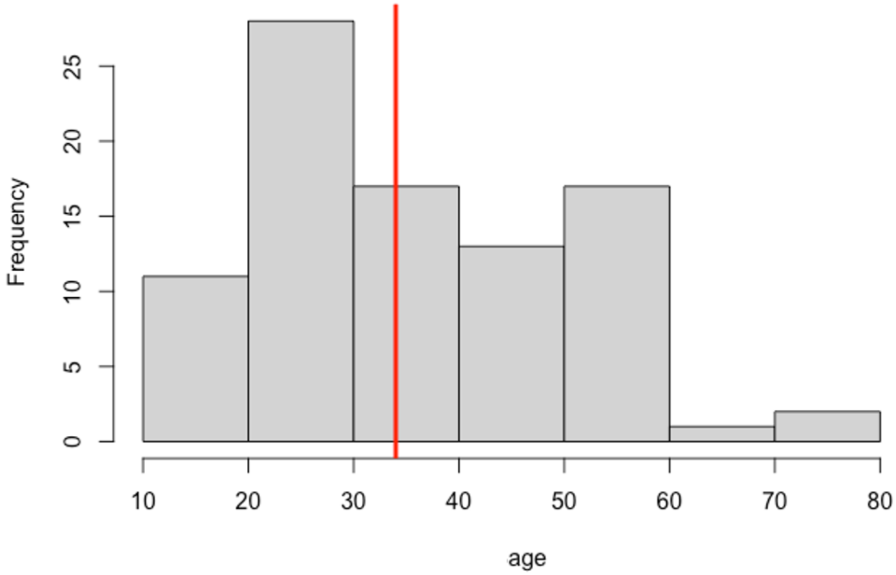
Frequency
0
5
10
15
20
25
10
20
30
40
50
60
70
80
age

a)

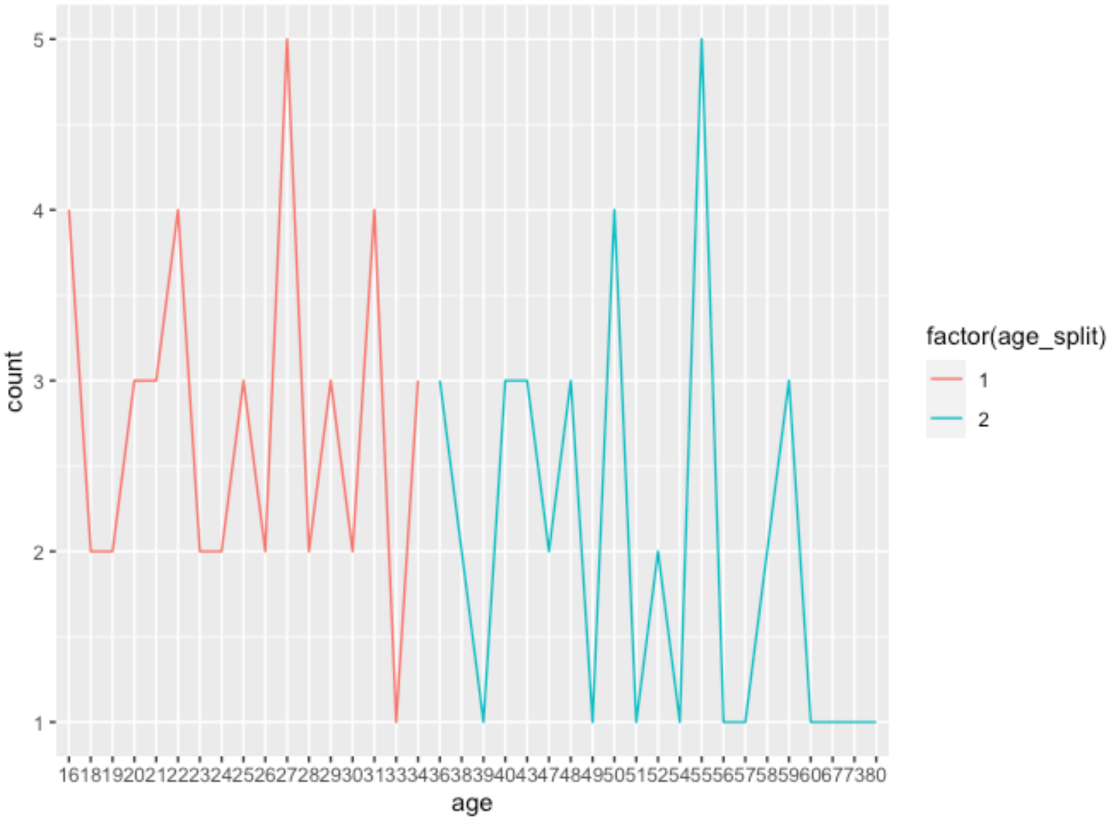
count
1
2
3
4
5
age
factor(age_split)
1
2

b)

Figure S2: a) histogram reporting the distribution of age in the whole sample of this study. The red line indicates the median value = 34. b) line graph representing the age distribution of the population with median split. In the legend, factor1: age<=34; factor2: age>34.

1.1. Bundles of interest

The bundles-of-interest (BOI) included the thalamic radiations and the corpus callosum (both subdivided by cortical target), the anterior commissure, the cingulum (subdivided into dorsal and ventral parts), the fornix, the inferior longitudinal fasciculus, middle longitudinal fasciculus, the pyramidal tract, the superior longitudinal fasciculus (I, II, and III), the arcuate fasciculus, the uncinate fasciculus, and the cerebellar peduncles (middle, inferior, and superior subdivisions).

2. Supplementary Results

2.1. Hemispheres considered together

2.1.1. Anxiety

Brain region	beta	pval	improvebeta	improvepval	qval
jhu_fa_Cerebral_peduncle	-5.8809142	0.00174461	5.491830448	0.289199872	0.01563193
jhu_fa_Posterior_limb_of_internal_capsule	-5.3565965	0.00231584	6.236281581	0.236000814	0.01563193
jhu_fa_External_capsule	-5.1521844	0.00136952	1.939910683	0.703506263	0.01563193
jhu_fa_Superior_longitudinal_fasciculus	-5.734935	0.00083286	2.296742363	0.647989138	0.01563193
scgm_fa_putamen	-6.2131147	0.00208182	0.473571886	0.927465277	0.01457273
ctx_thick_G_front_inf-Opercular	-7.8816762	6.88E-05	1.456604319	0.734481218	0.0053626
ctx_thick_Pole_occipital	-6.1940632	0.00097042	2.087327951	0.650595317	0.03784649
tract_thickmid_atr	4.58477121	0.00144984	4.923175711	0.336417959	0.03914568

Table S2: List of all the brain regions significantly associated with anxiety symptoms

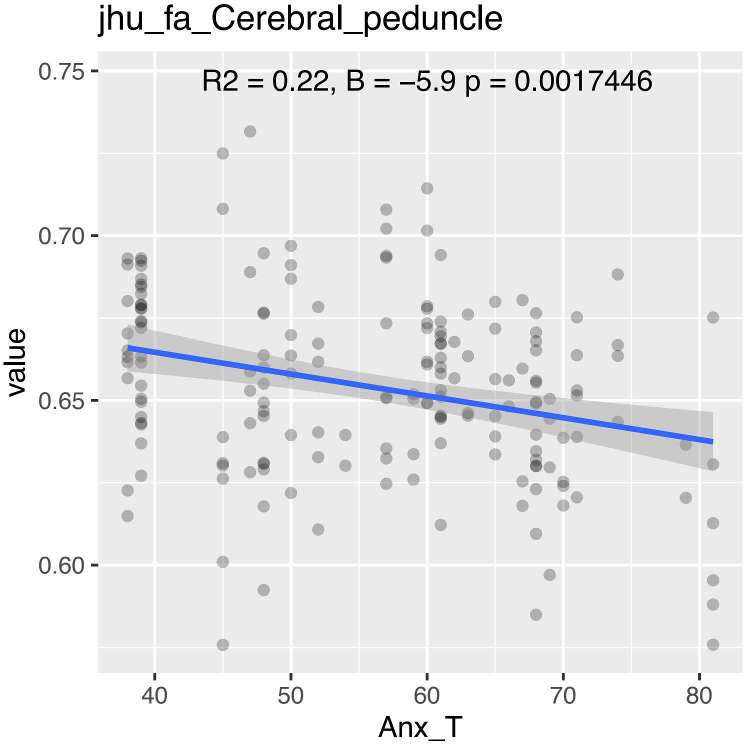
jhu_fa_Cerebral_peduncle
R2 = 0.22, B = −5.9 p = 0.0017446
value
0.75
0.70
0.65
0.60
40
50
60
70
80
Anx_T

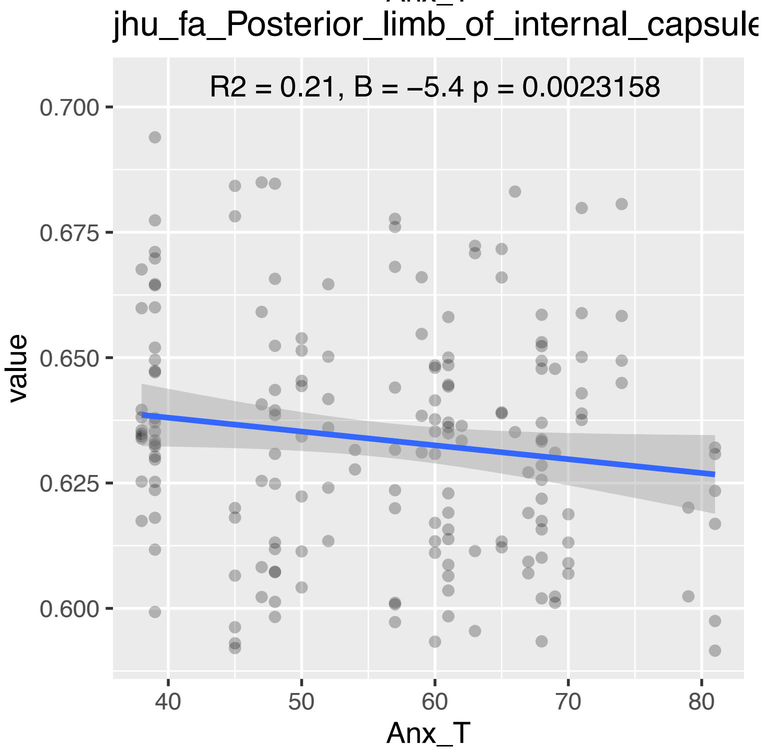
jhu_fa_Posterior_limb_of_internal_capsule
R2 = 0.21, B = −5.4 p = 0.0023158
value
0.700
0.675
0.650
0.625
0.600
40
50
60
70
80
Anx_T

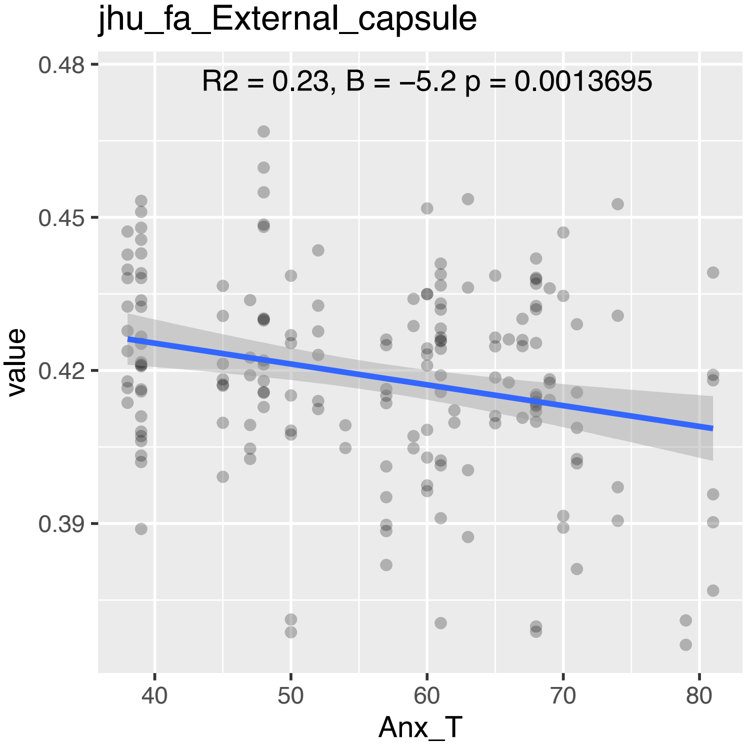
jhu_fa_External_capsule
R2 = 0.23, B = −5.2 p = 0.0013695
value
Anx_T
0.48
0.45
0.42
0.39
40
50
60
70
80

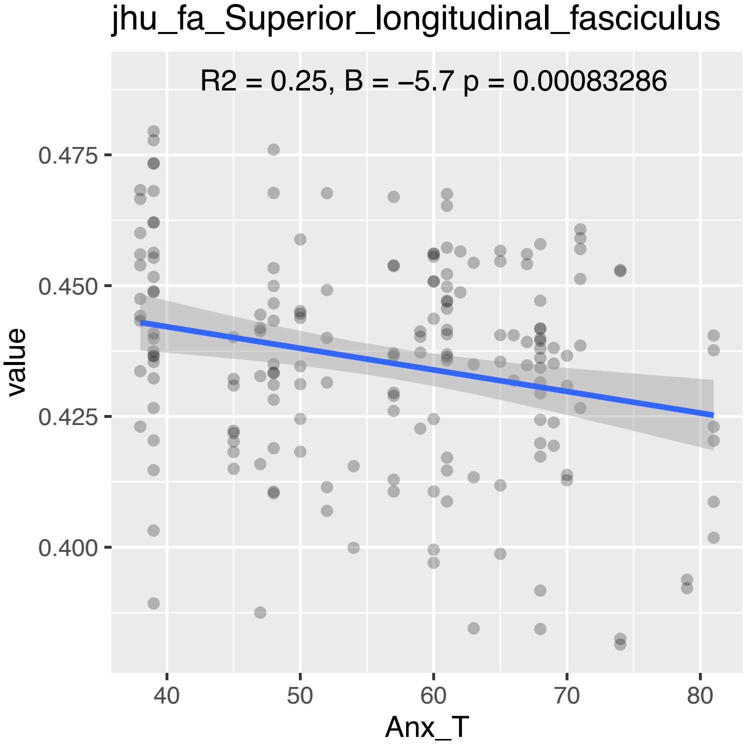
jhu_fa_Superior_longitudinal_fasciculus
R2 = 0.25, B = −5.7 p = 0.00083286
value
Anx_T
0.475
0.450
0.425
0.400
40
50
60
70
80

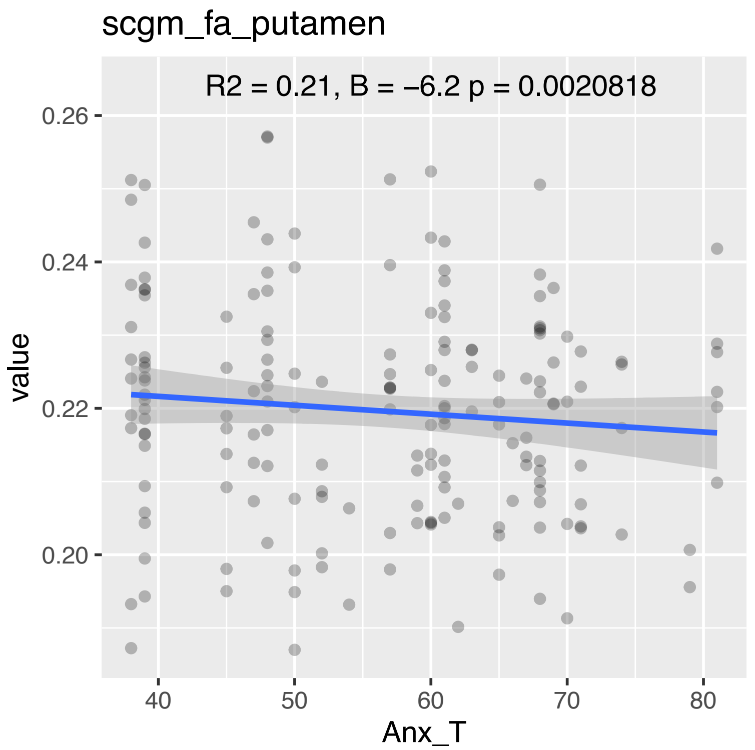
scgm_fa_putamen
R2 = 0.21, B = −6.2 p = 0.0020818
0.26
0.24
0.22
0.20
value
40
50
60
70
80
Anx_T

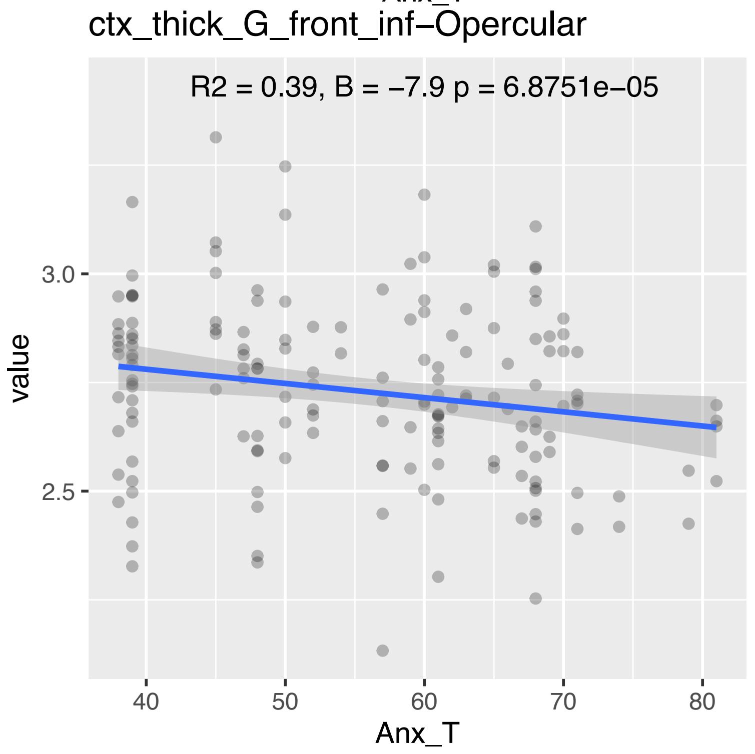
ctx_thick_G_front_inf−Opercular
R2 = 0.39, B = −7.9 p = 6.8751e−05
3.0
2.5
value
40
50
60
70
80
Anx_T

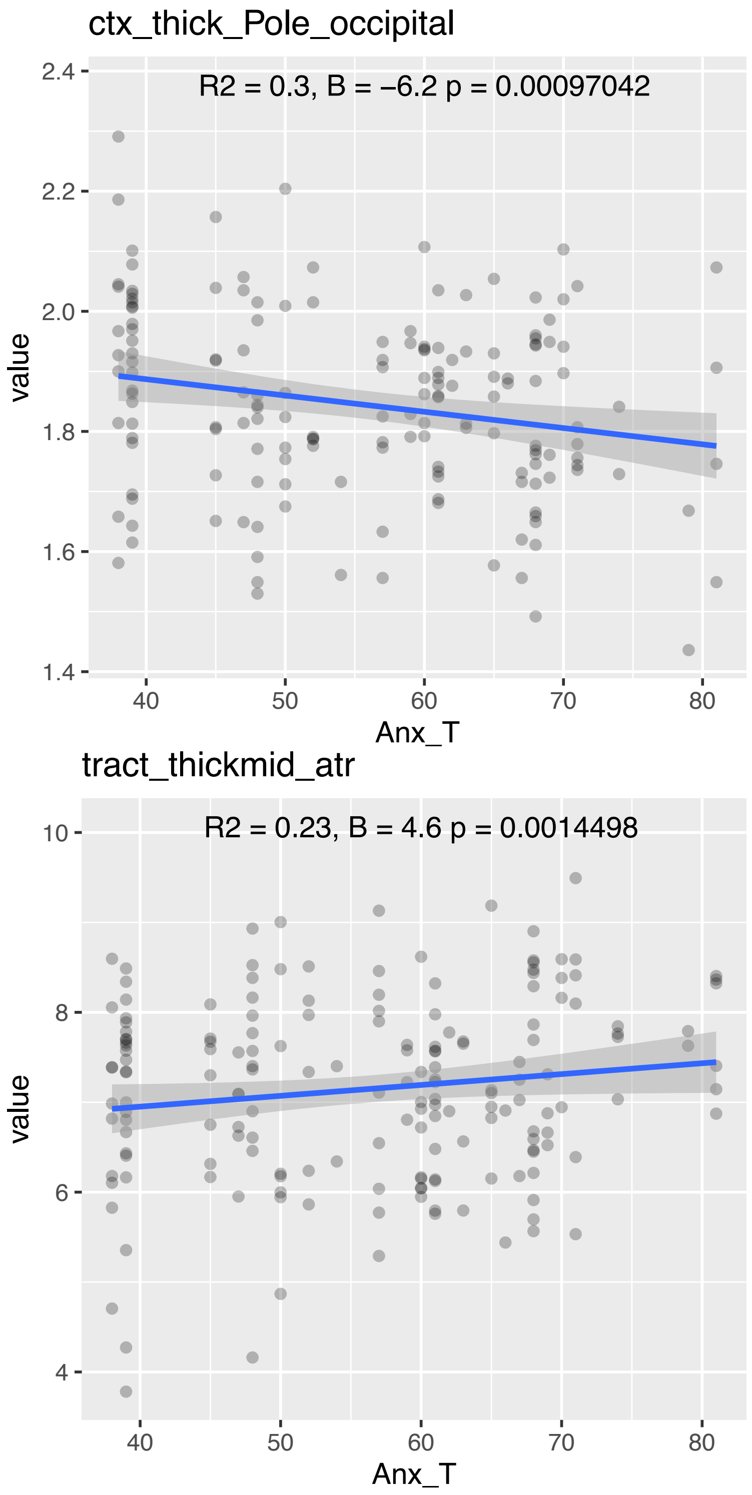

Figure S3: Scatterplots indicating brain regions significantly associated to anxiety symptoms (here hemispheres are analyzed together)

2.1.2. Depression

Brain region	beta	p-val	improvebeta	improvepval	q-val
jhu_fa_Cerebral_peduncle	-4.8240643	0.00236701	-6.93797223	0.117487403	0.03195457
jhu_fa_Posterior_limb_of_internal_capsule	-5.8494325	3.34E-05	-5.54891636	0.166454493	0.00090303
ctx_thick_G_and_S_subcentral	-4.6548315	0.00910932	-6.384911706	0.143049955	0.03808447
ctx_thick_G_and_S_transv_frontopol	-5.1814533	0.00466919	-8.020829414	0.05925998	0.0260537
ctx_thick_G_cingul-Post-dorsal	-4.7018592	0.00335759	-9.353599652	0.027944753	0.02325921
ctx_thick_G_cuneus	-4.8563135	0.00064811	-11.61536045	0.006068026	0.00842547
ctx_thick_G_front_inf-Opercular	-6.5262097	0.00016564	-10.31958317	0.009786511	0.00323006
ctx_thick_G_front_sup	-5.9518855	0.00551606	-12.17207364	0.007547338	0.02868349
ctx_thick_G_insular_short	-6.8825652	4.23E-06	-5.454077735	0.122594066	0.00033008
ctx_thick_G_pariet_inf-Supramar	-6.8452433	0.00120428	-6.888792	0.096748117	0.01314719
ctx_thick_G_parietal_sup	-4.9143634	0.00349794	-10.82818626	0.012953673	0.02325921
ctx_thick_G_precentral	-5.8110498	0.00181082	-6.60192398	0.119693671	0.01569375
ctx_thick_G_temporal_middle	-5.607517	0.00631855	-7.299932114	0.092212609	0.03080291
ctx_thick_Pole_occipital	-4.8125322	0.00357834	-9.728036413	0.023082096	0.02325921
ctx_thick_S_circular_insula_inf	-6.5966544	8.33E-05	-10.76703868	0.006454931	0.00246237
ctx_thick_S_circular_insula_sup	-6.1897324	9.47E-05	-11.50956848	0.004174687	0.00246237
ctx_thick_S_orbital_med-olfact	-3.2873395	0.00809041	-11.60238148	0.009245951	0.03712072
ctx_thick_S_precentral-inf-part	-5.2088956	0.00134843	-10.7955883	0.011027352	0.01314719
ctx_thick_S_subparietal	-5.3093257	0.01060803	-8.314410002	0.055594613	0.04137133
ctx_thick_S_temporal_sup	-5.8351261	0.0046763	-10.75958649	0.016348928	0.0260537
ctx_thick_MeanThickness	-7.572872	0.00040771	-13.19716411	0.002334135	0.00636028
ctx_thick_rhat_Fis-ant-Horizont	-6.2282883	0.00927699	-4.88974245	0.414698848	0.03808447

Table S3: list of all the brain regions that are significantly associated to depressive symptoms in mTBI patients.

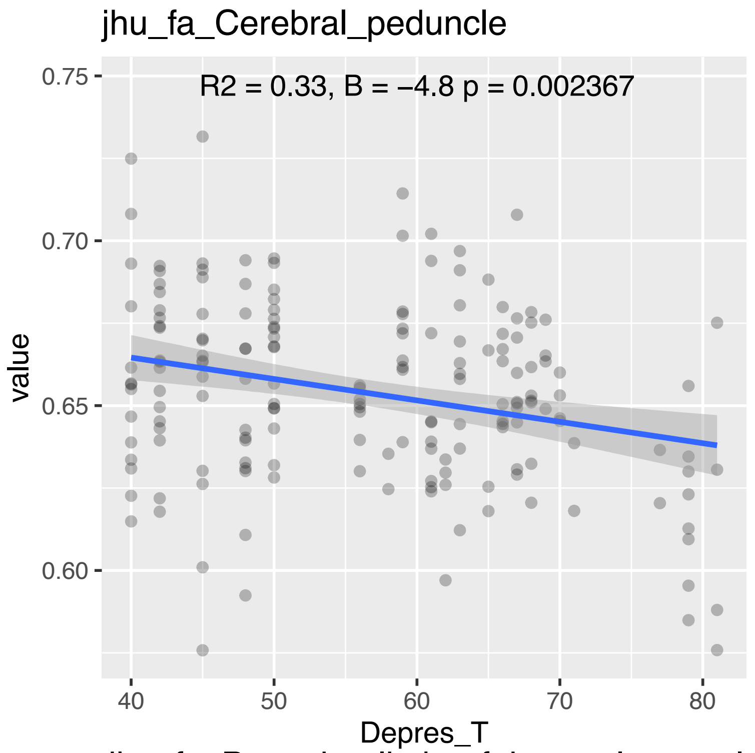
jhu_fa_Cerebral_peduncle
R2 = 0.33, B = −4.8 p = 0.002367
0.75
0.70
0.65
0.60
value
40
50
60
70
80
Depres_T

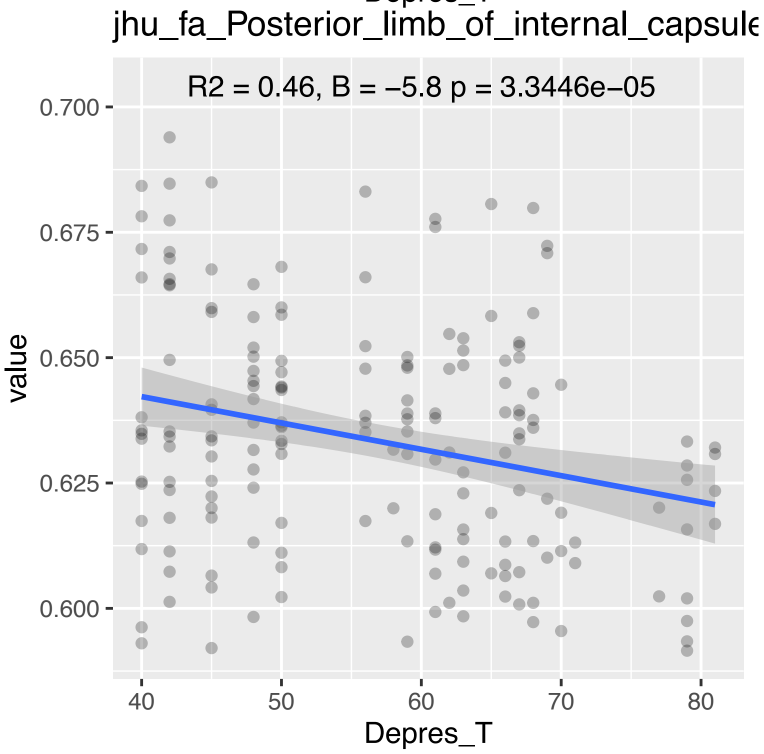
jhu_fa_Posterior_limb_of_internal_capsule
R2 = 0.46, B = −5.8 p = 3.3446e−05
0.700
0.675
0.650
0.625
0.600
value
40
50
60
70
80
Depres_T

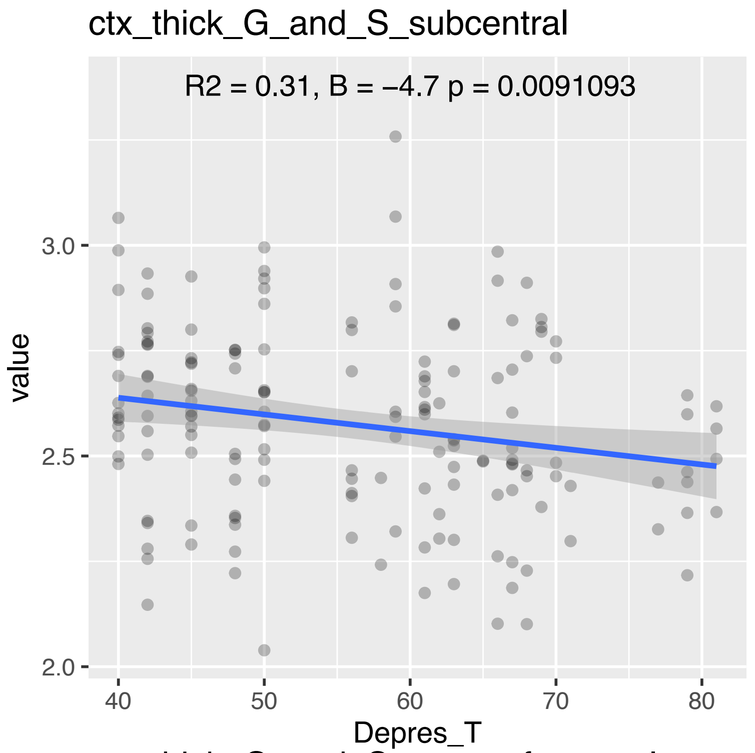
ctx_thick_G_and_S_subcentral
R2 = 0.31, B = −4.7 p = 0.0091093
value
3.0
2.5
2.0
40
50
60
70
80
Depres_T

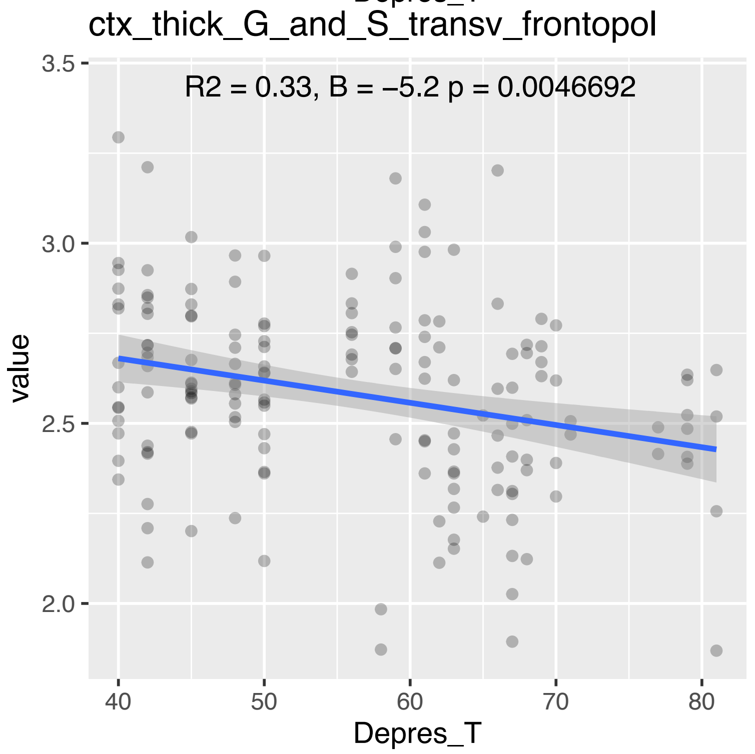
ctx_thick_G_and_S_transv_frontopol
R2 = 0.33, B = −5.2 p = 0.0046692
value
3.5
3.0
2.5
2.0
40
50
60
70
80
Depres_T

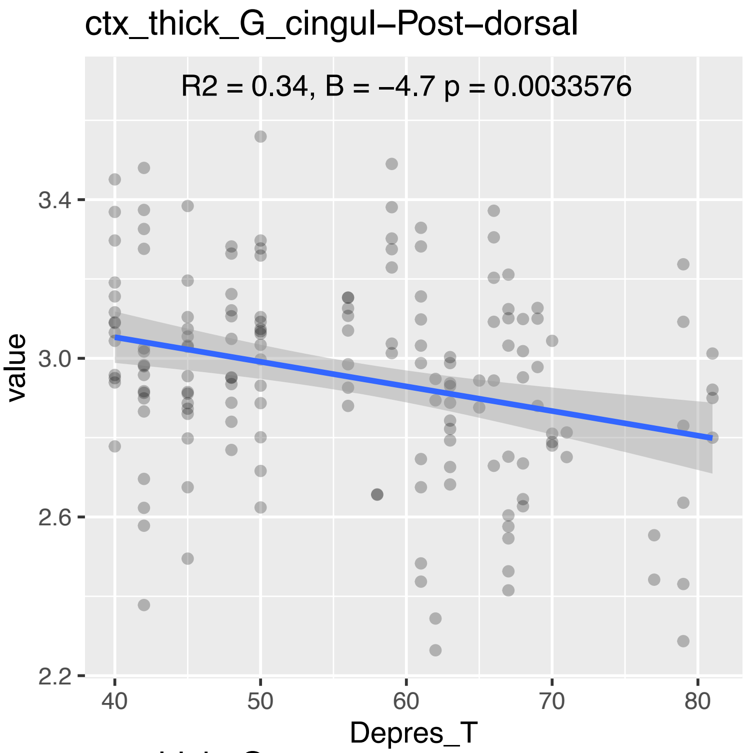
ctx_thick_G_cingul−Post−dorsal
R2 = 0.34, B = −4.7 p = 0.0033576
value
3.4
3.0
2.6
2.2
40
50
60
70
80
Depres_T

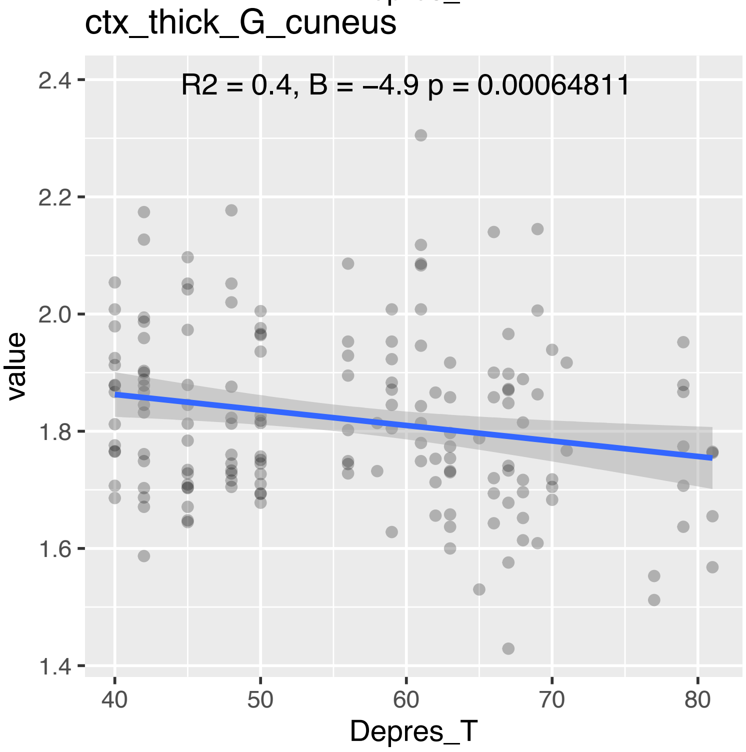
ctx_thick_G_cuneus
R2 = 0.4, B = −4.9 p = 0.00064811
value
2.4
2.2
2.0
1.8
1.6
1.4
40
50
60
70
80
Depres_T

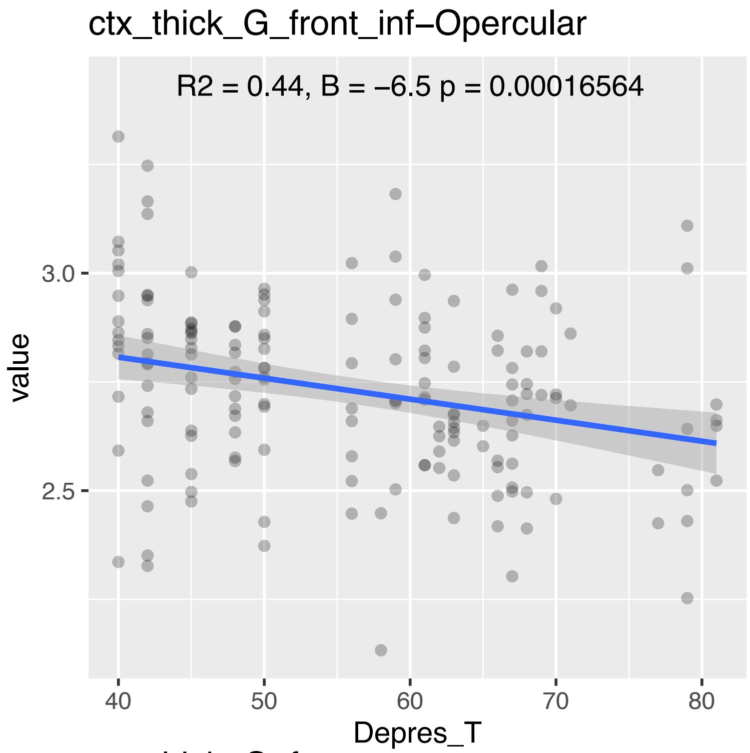
ctx_thick_G_front_inf−Opercular
R2 = 0.44, B = −6.5 p = 0.00016564
value
3.0
2.5
40
50
60
70
80
Depres_T

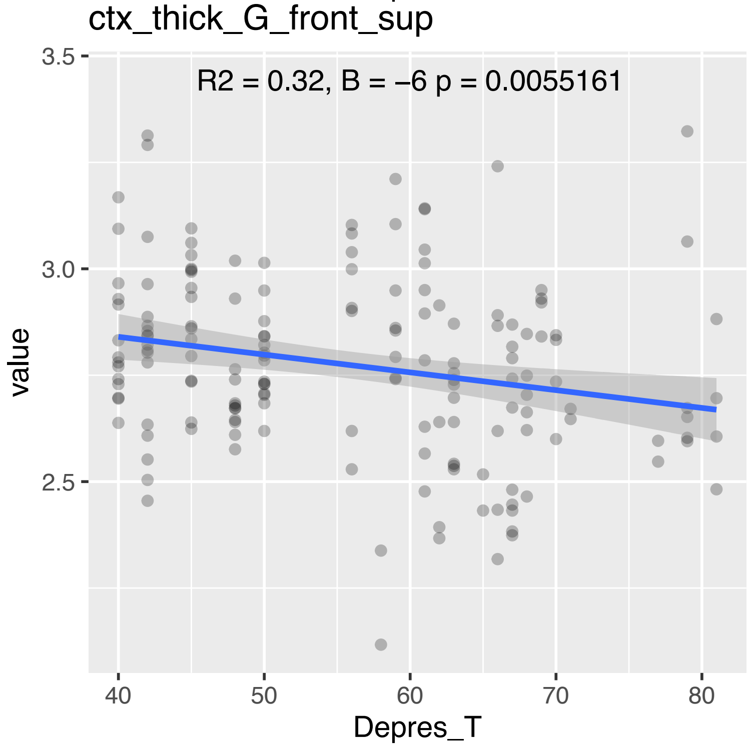
ctx_thick_G_front_sup
R2 = 0.32, B = −6 p = 0.0055161
value
3.5
3.0
2.5
40
50
60
70
80
Depres_T

ctx_thick_G_insular_short

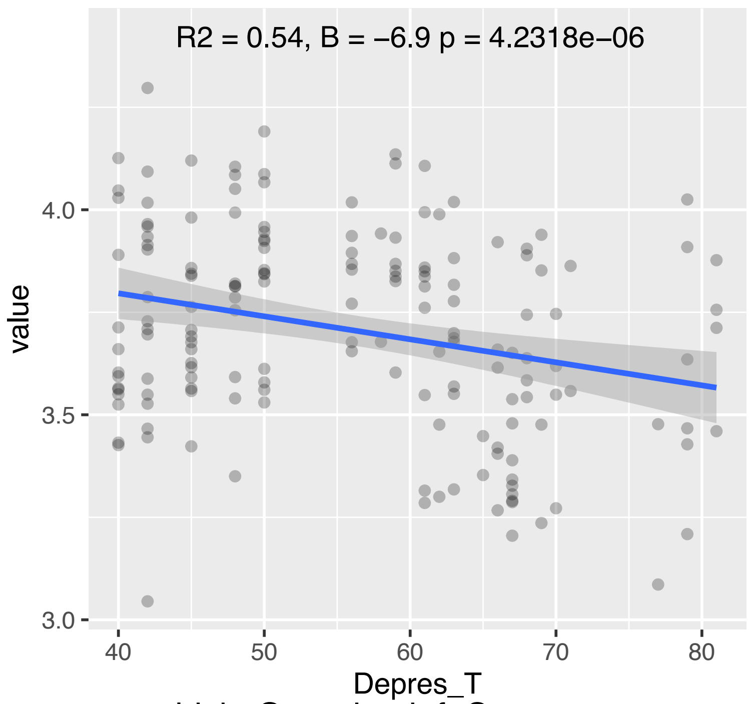

ctx_thick_G_pariet_inf−Supramar

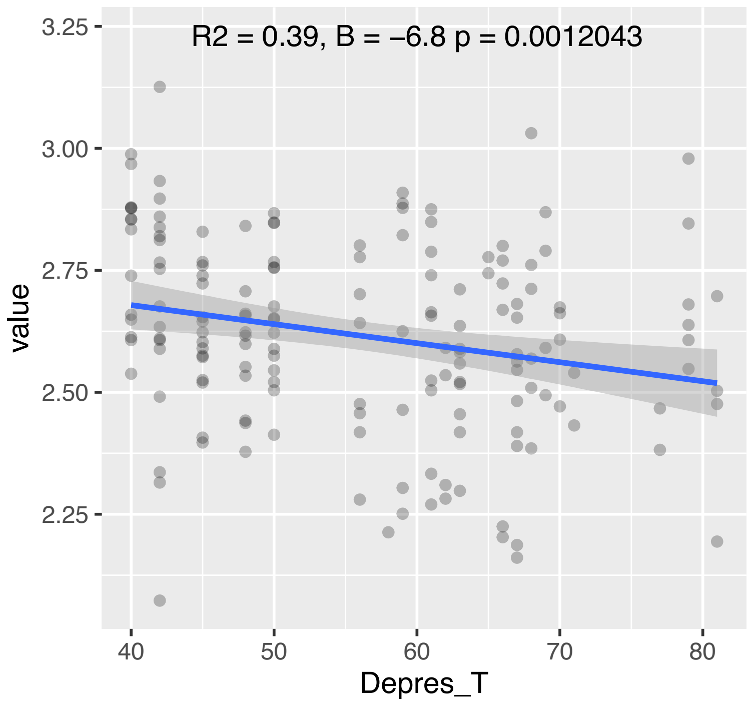

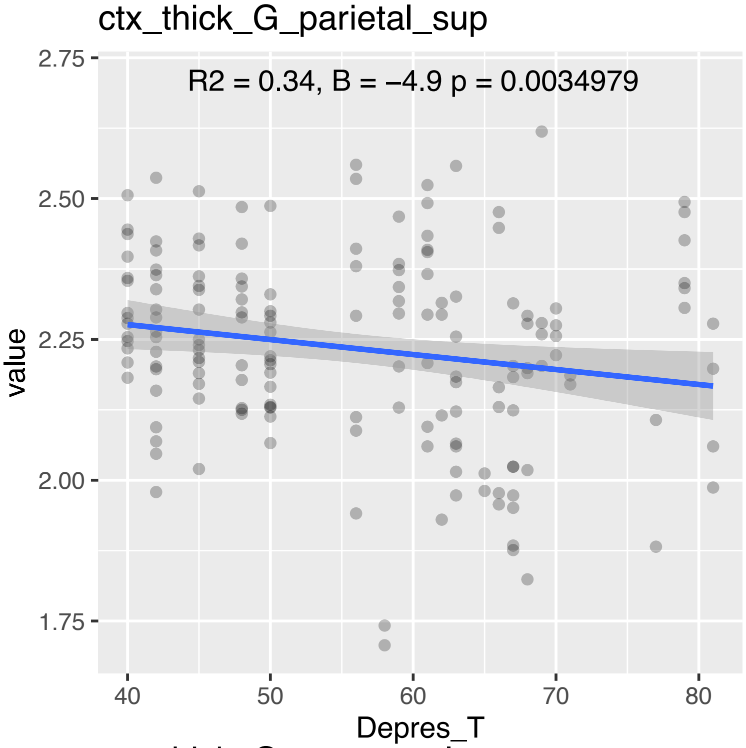
ctx_thick_G_parietal_sup
R2 = 0.34, B = −4.9 p = 0.0034979
2.75
2.50
2.25
2.00
1.75
value
40
50
60
70
80
Depres_T

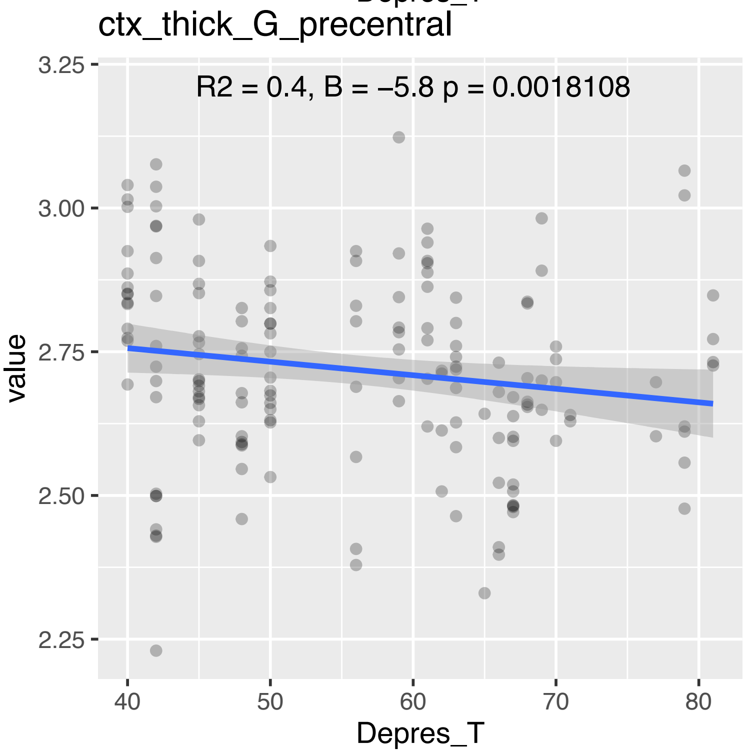
ctx_thick_G_precentral
R2 = 0.4, B = −5.8 p = 0.0018108
3.25
3.00
2.75
2.50
2.25
value
40
50
60
70
80
Depres_T

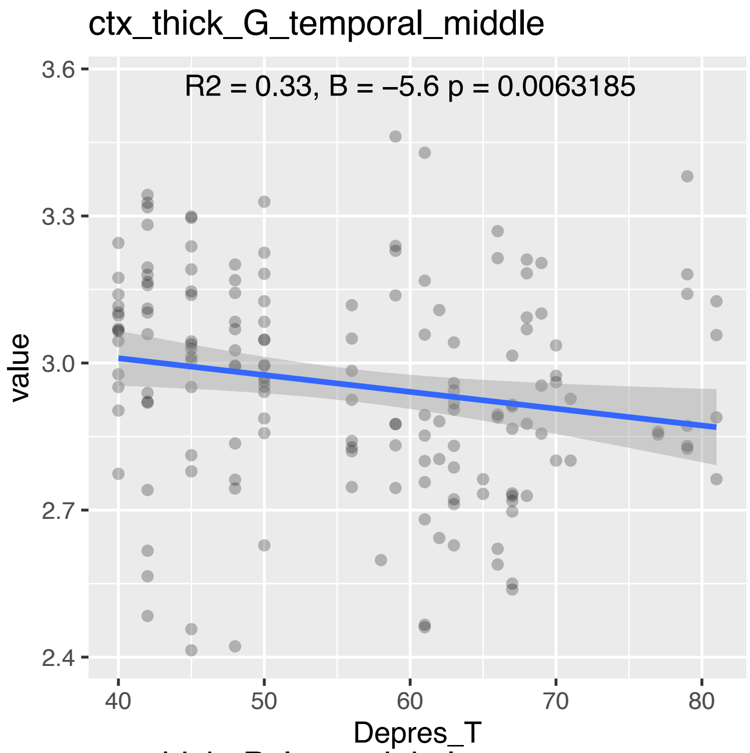
ctx_thick_G_temporal_middle
R2 = 0.33, B = −5.6 p = 0.0063185
value
3.6
3.3
3.0
2.7
2.4
40
50
60
70
80
Depres_T

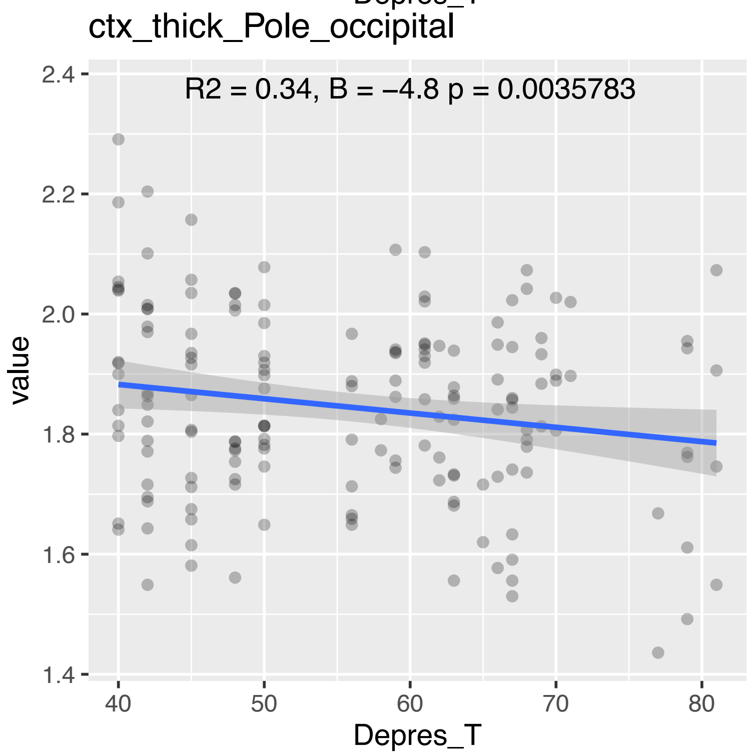
ctx_thick_Pole_occipital
R2 = 0.34, B = −4.8 p = 0.0035783
value
2.4
2.2
2.0
1.8
1.6
1.4
40
50
60
70
80
Depres_T

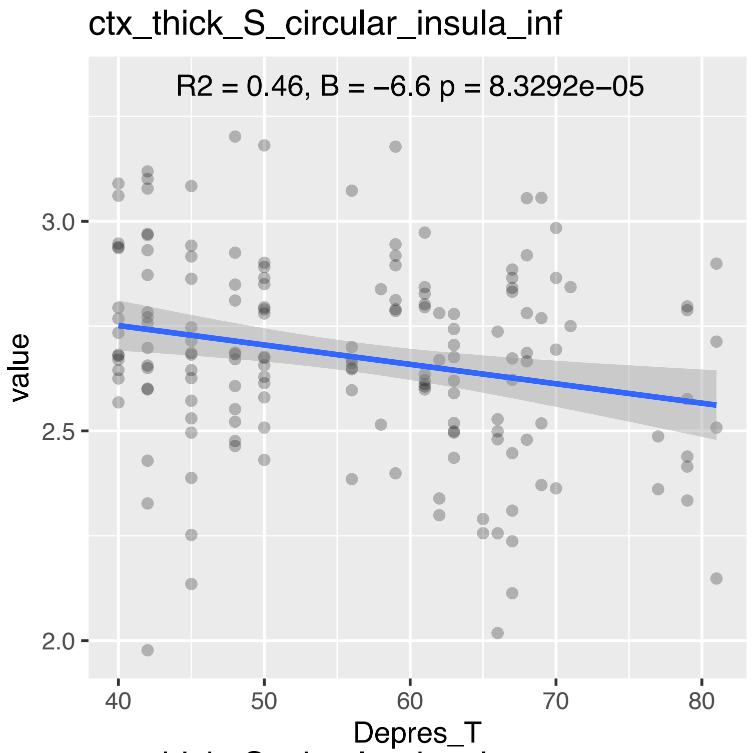
ctx_thick_S_circular_insula_inf
R2 = 0.46, B = −6.6 p = 8.3292e−05
value
3.0
2.5
2.0
40
50
60
70
80
Depres_T

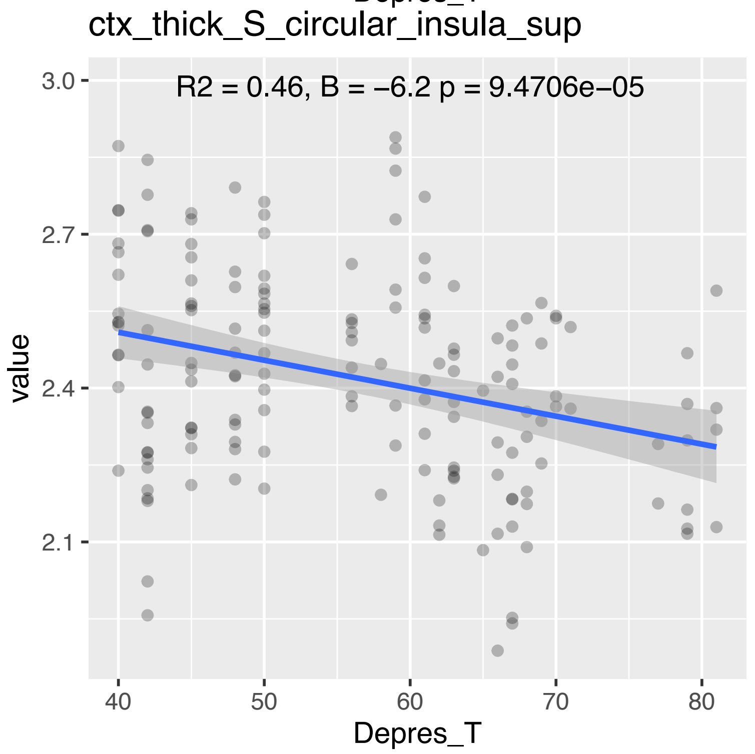
ctx_thick_S_circular_insula_sup
R2 = 0.46, B = −6.2 p = 9.4706e−05
value
3.0
2.7
2.4
2.1
40
50
60
70
80
Depres_T

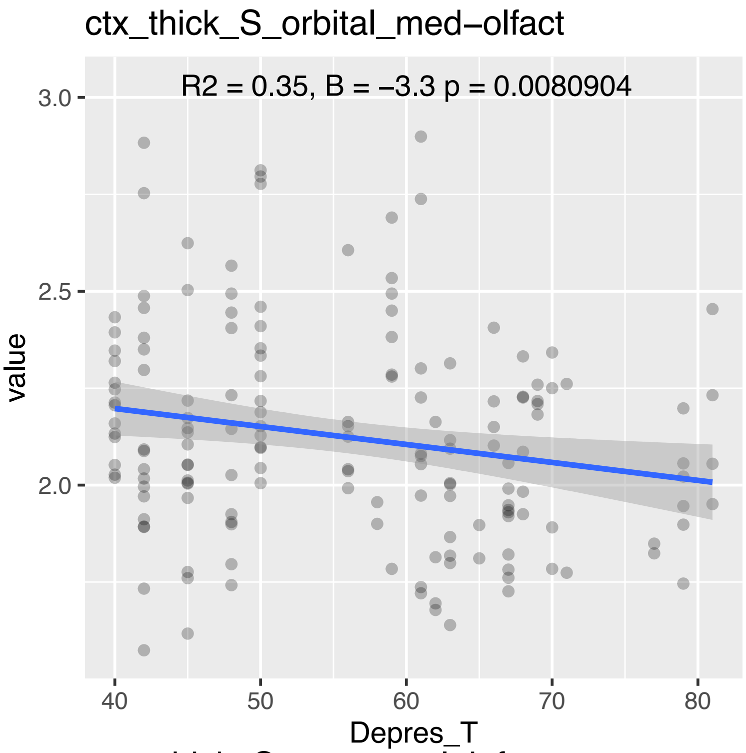
ctx_thick_S_orbital_med−olfact
R2 = 0.35, B = −3.3 p = 0.0080904
value
Depres_T
3.0
2.5
2.0
40
50
60
70
80

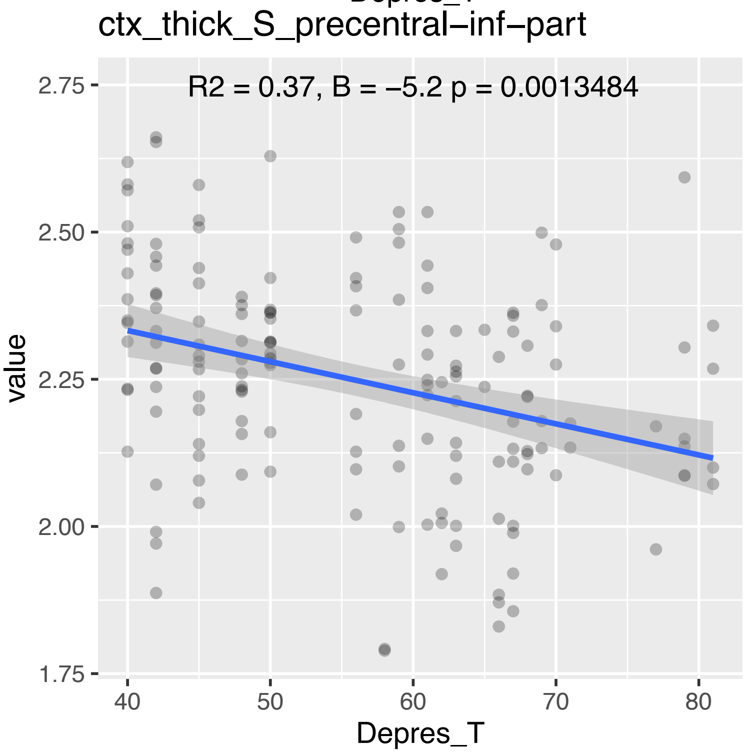
ctx_thick_S_precentral−inf−part
R2 = 0.37, B = −5.2 p = 0.0013484
value
Depres_T
2.75
2.50
2.25
2.00
1.75
40
50
60
70
80

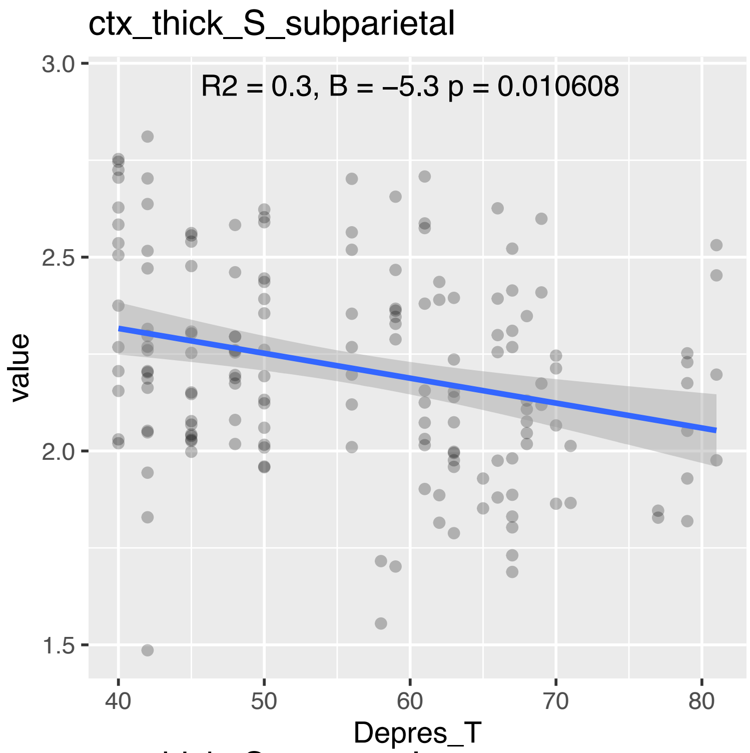

ctx_thick_S_subparietal
R2 = 0.3, B = −5.3 p = 0.010608
value
Depres_T
3.0
2.5
2.0
1.5
40
50
60
70
80

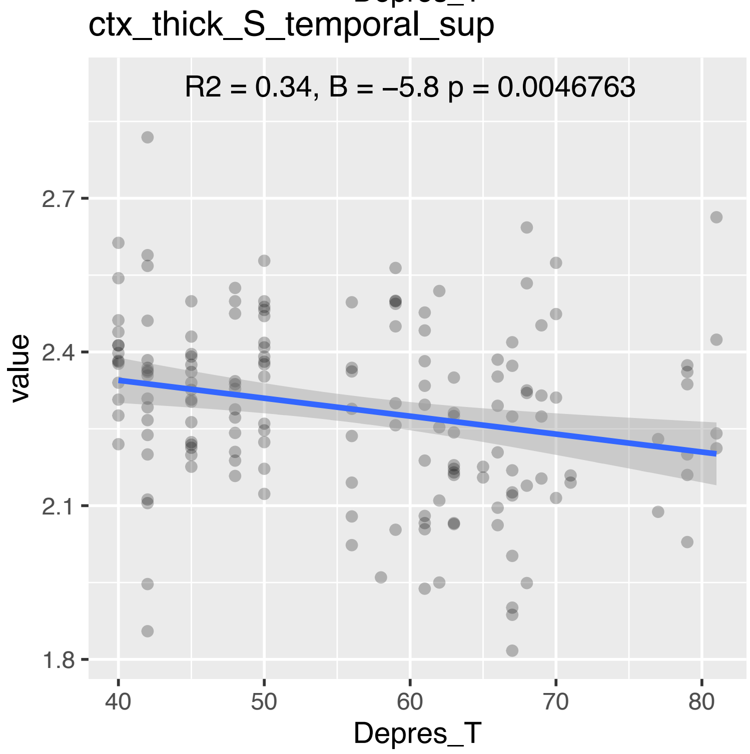

ctx_thick_S_temporal_sup
R2 = 0.34, B = −5.8 p = 0.0046763
value
Depres_T
2.7
2.4
2.1
1.8
40
50
60
70
80

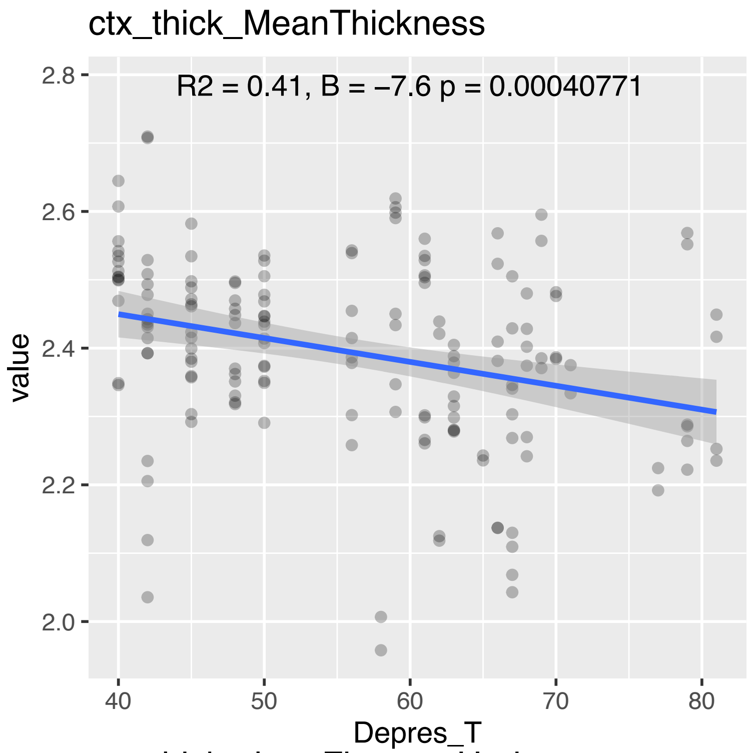
ctx_thick_MeanThickness
R2 = 0.41, B = −7.6 p = 0.00040771
2.8
2.6
2.4
2.2
2.0
value
40
50
60
70
80
Depres_T

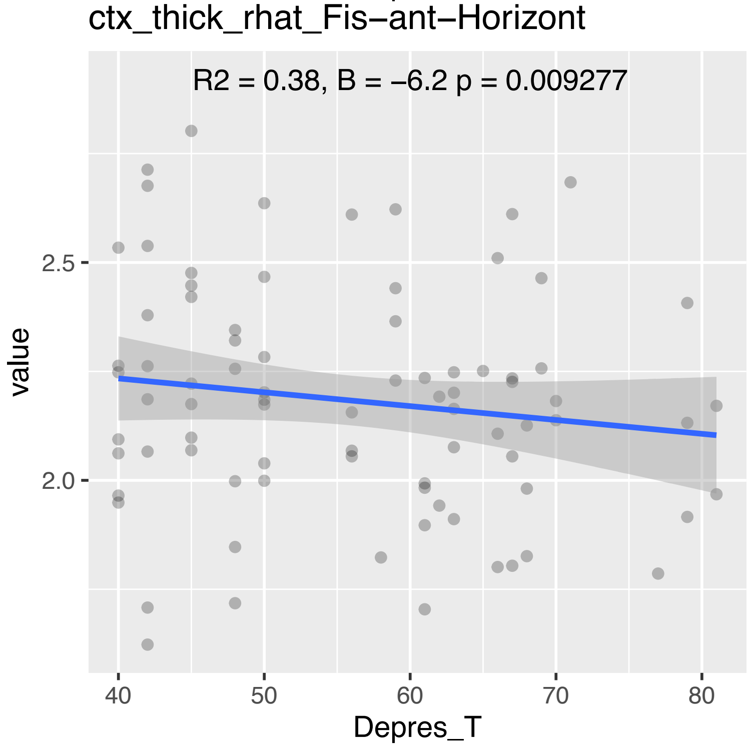
ctx_thick_rhat_Fis−ant−Horizont
R2 = 0.38, B = −6.2 p = 0.009277
2.5
2.0
value
40
50
60
70
80
Depres_T

Figure S4: Scatterplots indicating the significant relationships between depression and brain regions (here hemispheres are analyzed together)

2.1.3. Somatization

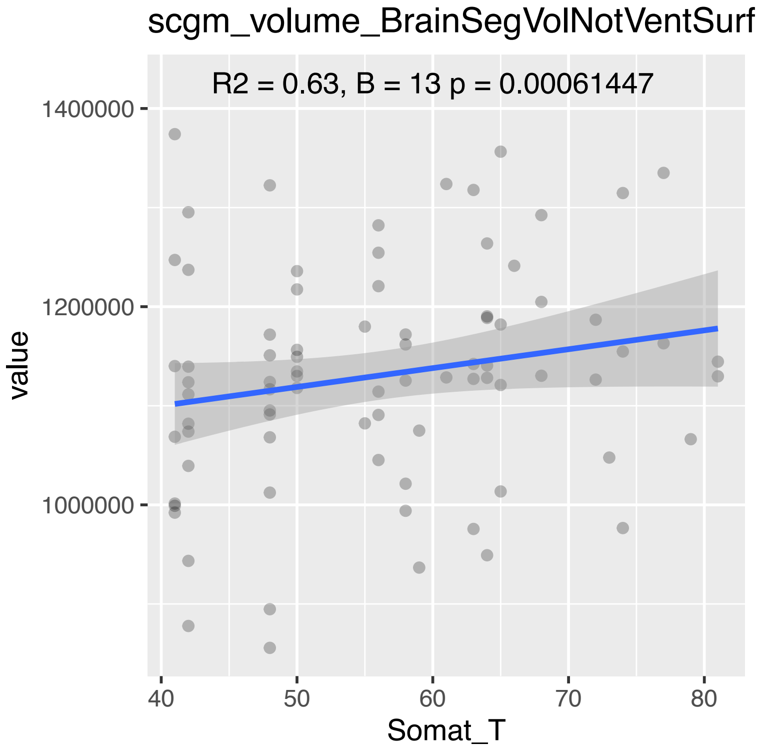

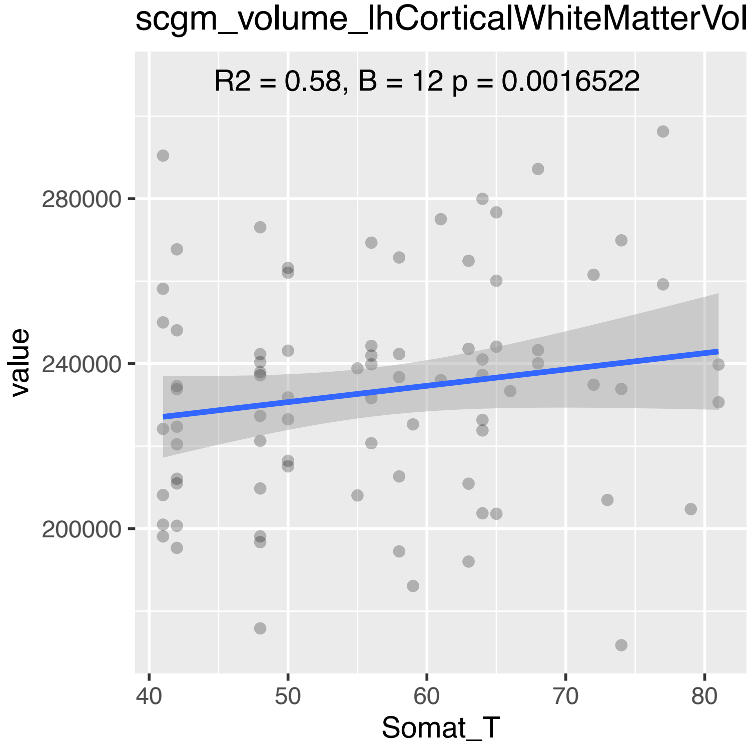

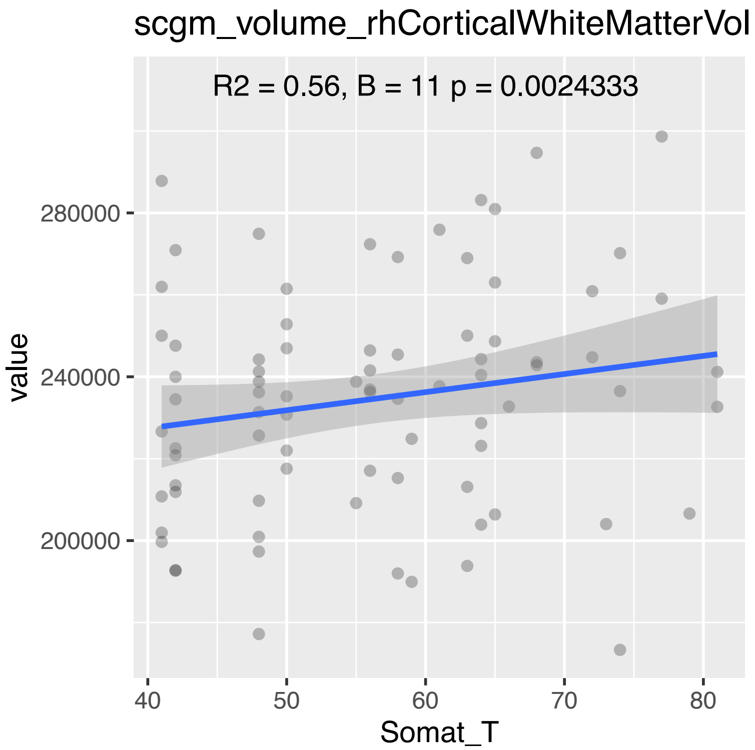
scgm_volume_rhCorticalWhiteMatterVol
R2 = 0.56, B = 11 p = 0.0024333
value
280000
240000
200000
40
50
60
70
80
Somat_T

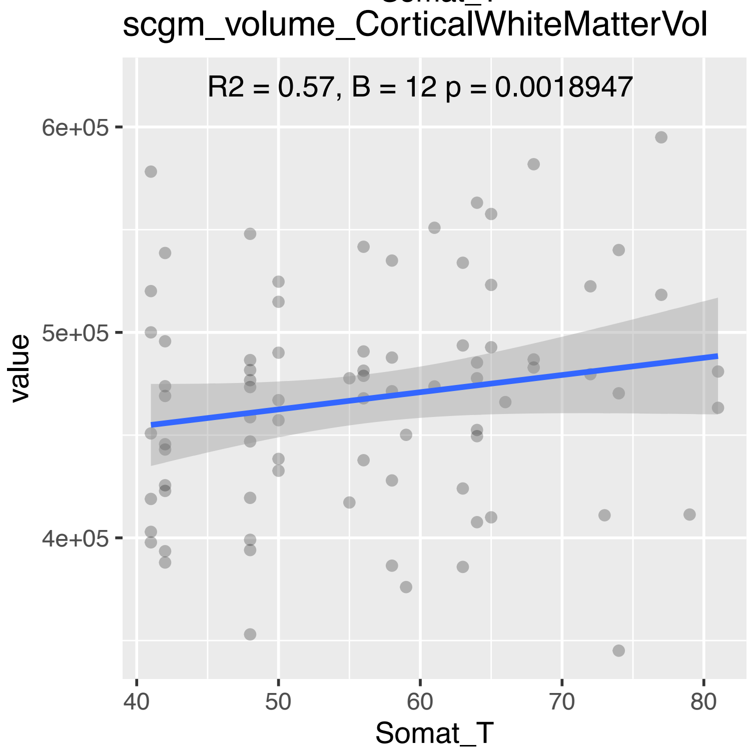
scgm_volume_CorticalWhiteMatterVol
R2 = 0.57, B = 12 p = 0.0018947
value
6e+05
5e+05
4e+05
40
50
60
70
80
Somat_T

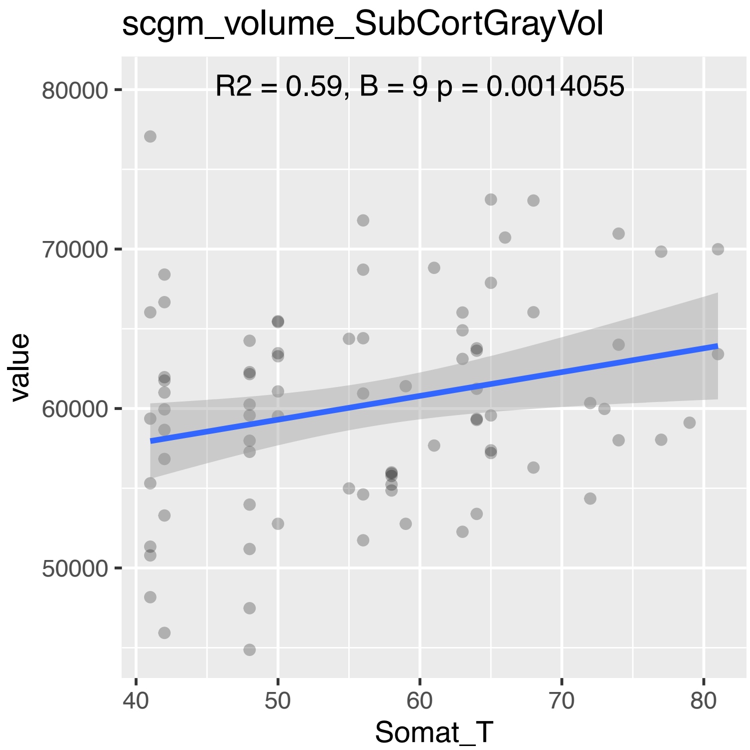

scgm_volume_SubCortGrayVol
R2 = 0.59, B = 9 p = 0.0014055
80000
70000
60000
50000
value
40
50
60
70
80
Somat_T

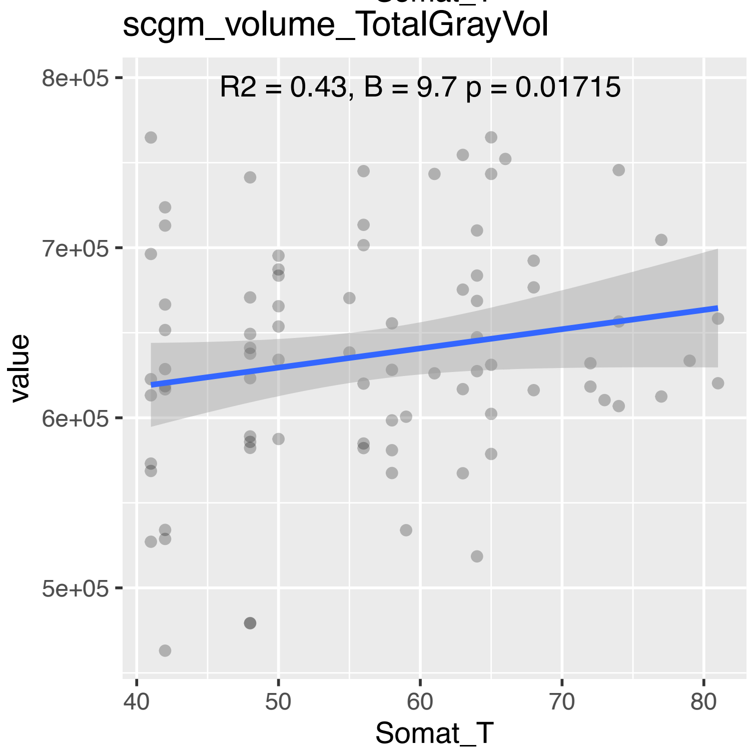

scgm_volume_TotalGrayVol
R2 = 0.43, B = 9.7 p = 0.01715
8e+05
7e+05
6e+05
5e+05
value
40
50
60
70
80
Somat_T

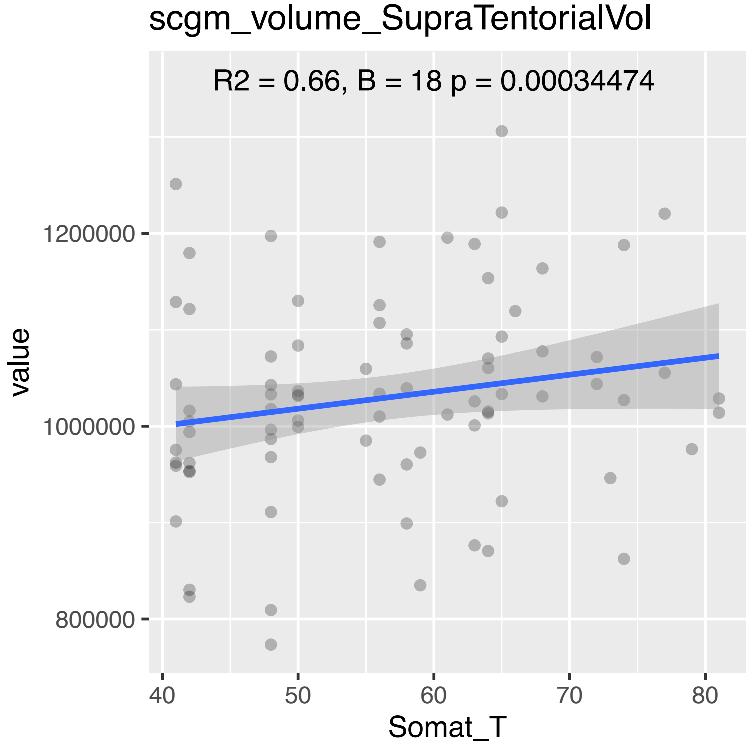
scgm_volume_SupraTentorialVol
R2 = 0.66, B = 18 p = 0.00034474
value
1200000
1000000
800000
40
50
60
70
80
Somat_T

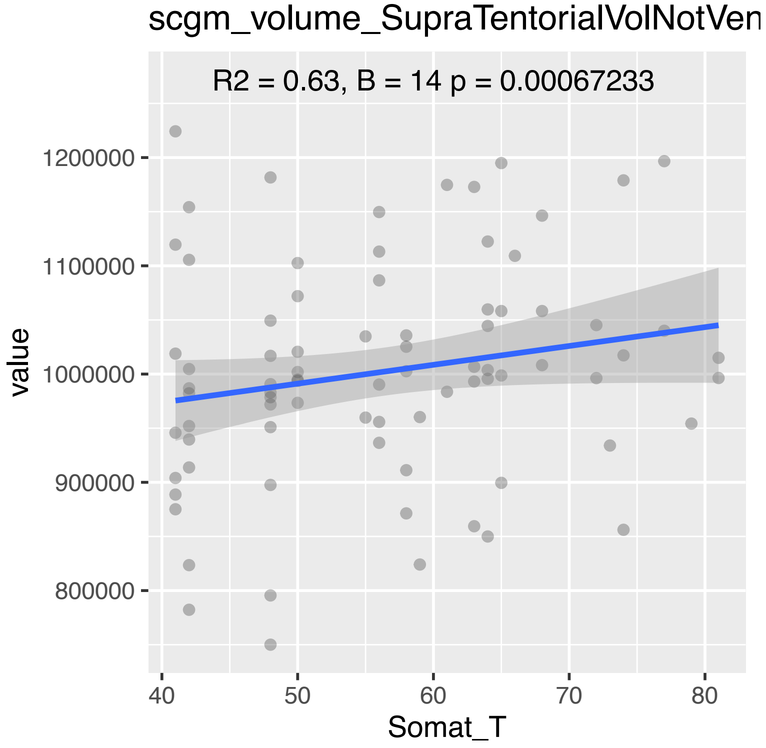
scgm_volume_SupraTentorialVolNotVen
R2 = 0.63, B = 14 p = 0.00067233
value
1200000
1100000
1000000
900000
800000
40
50
60
70
80
Somat_T

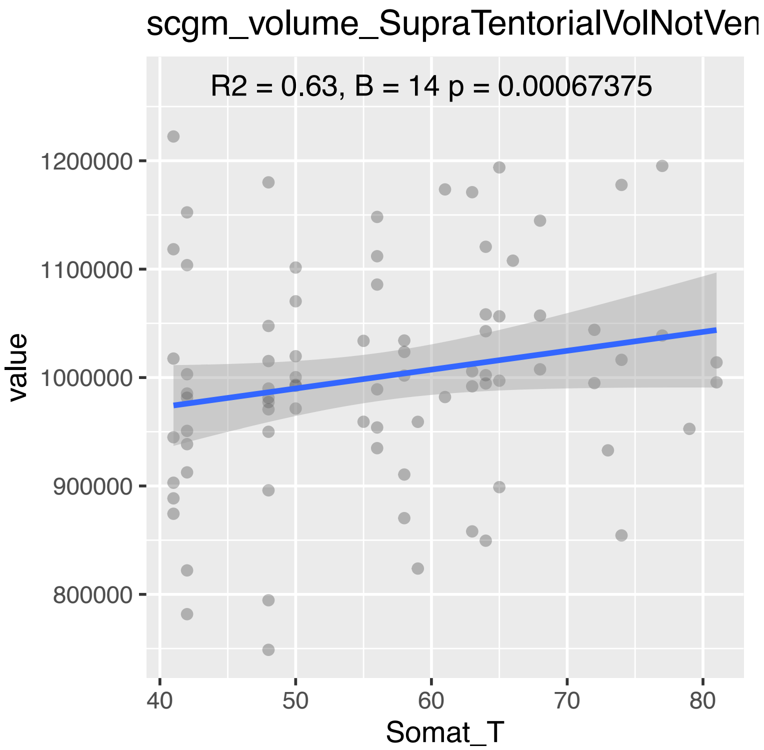
scgm_volume_SupraTentorialVolNotVen
R2 = 0.63, B = 14 p = 0.00067375
1200000
1100000
1000000
900000
800000
value
40
50
60
70
80
Somat_T

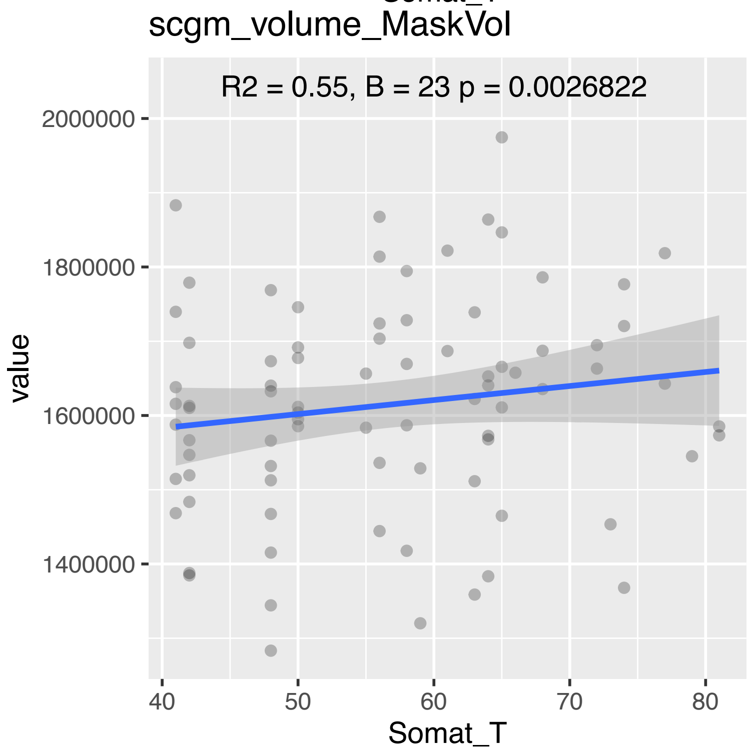
scgm_volume_MaskVol
R2 = 0.55, B = 23 p = 0.0026822
2000000
1800000
1600000
1400000
value
40
50
60
70
80
Somat_T

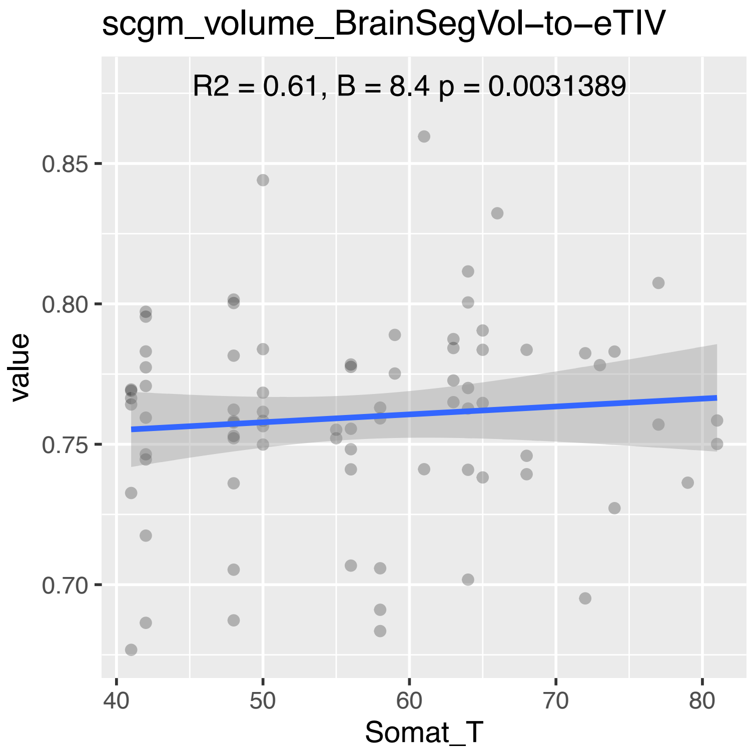
scgm_volume_BrainSegVol−to−eTIV
R2 = 0.61, B = 8.4 p = 0.0031389
0.85
0.80
0.75
0.70
value
40
50
60
70
80
Somat_T

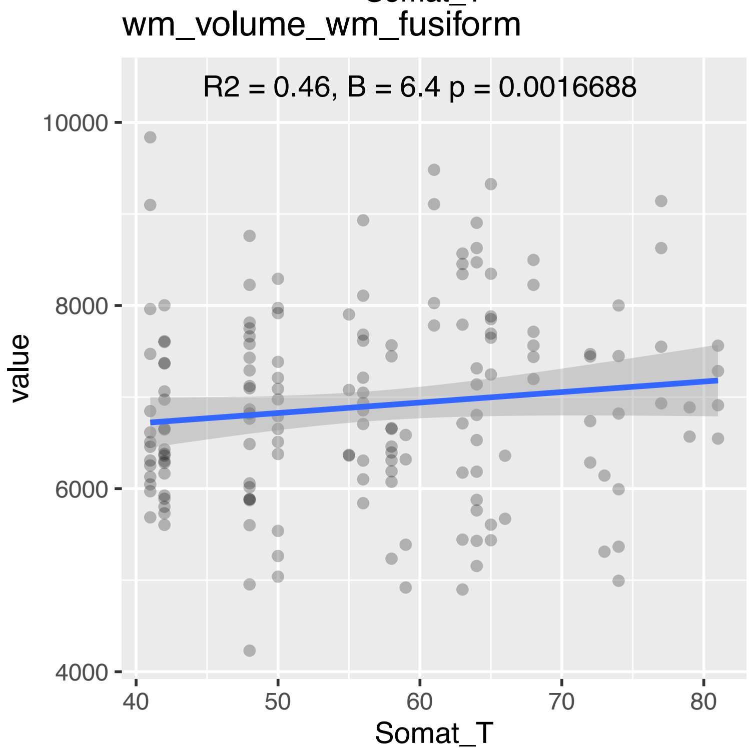
wm_volume_wm_fusiform
R2 = 0.46, B = 6.4 p = 0.0016688
10000
8000
6000
4000
value
40
50
60
70
80
Somat_T

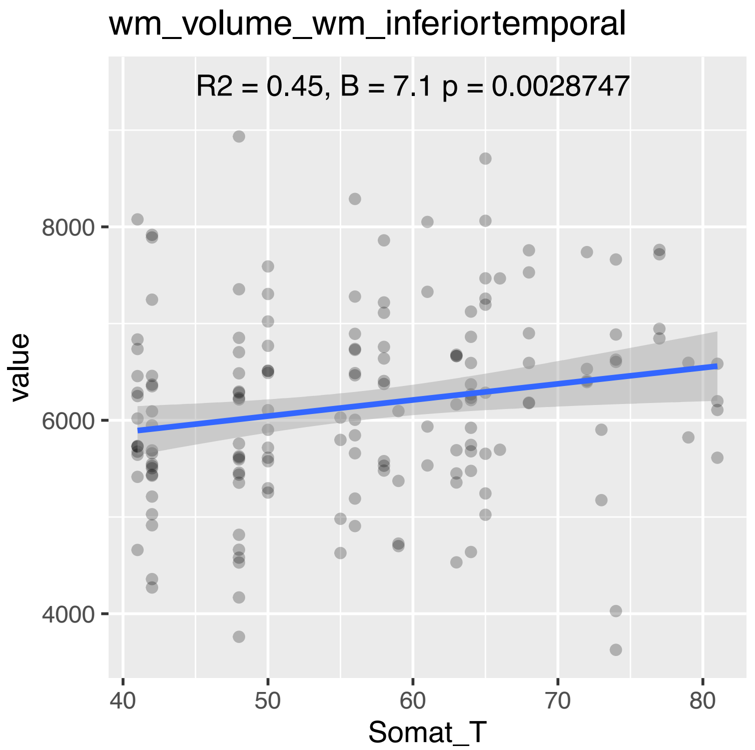
wm_volume_wm_inferiortemporal
R2 = 0.45, B = 7.1 p = 0.0028747
value
8000
6000
4000
40
50
60
70
80
Somat_T

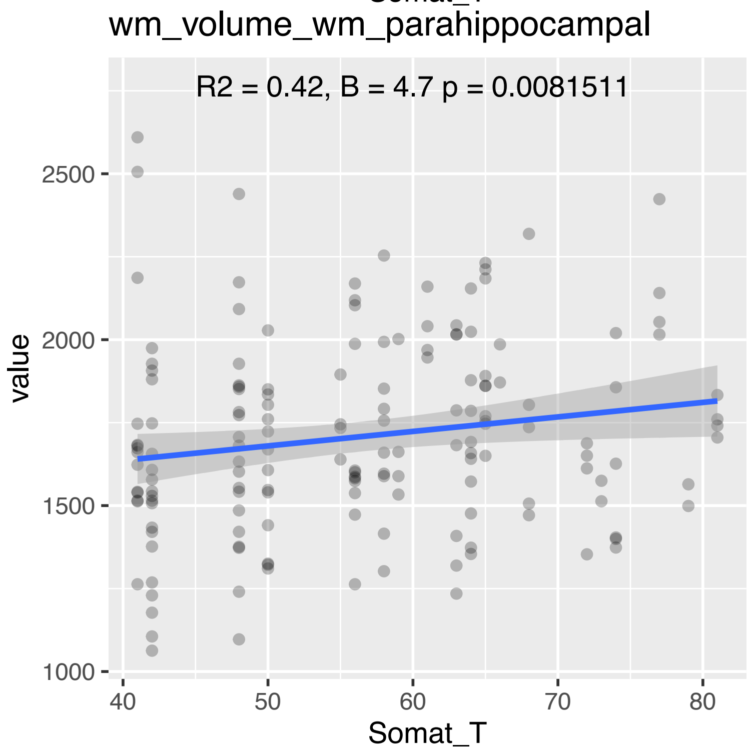
wm_volume_wm_parahippocampal
R2 = 0.42, B = 4.7 p = 0.0081511
value
2500
2000
1500
1000
40
50
60
70
80
Somat_T

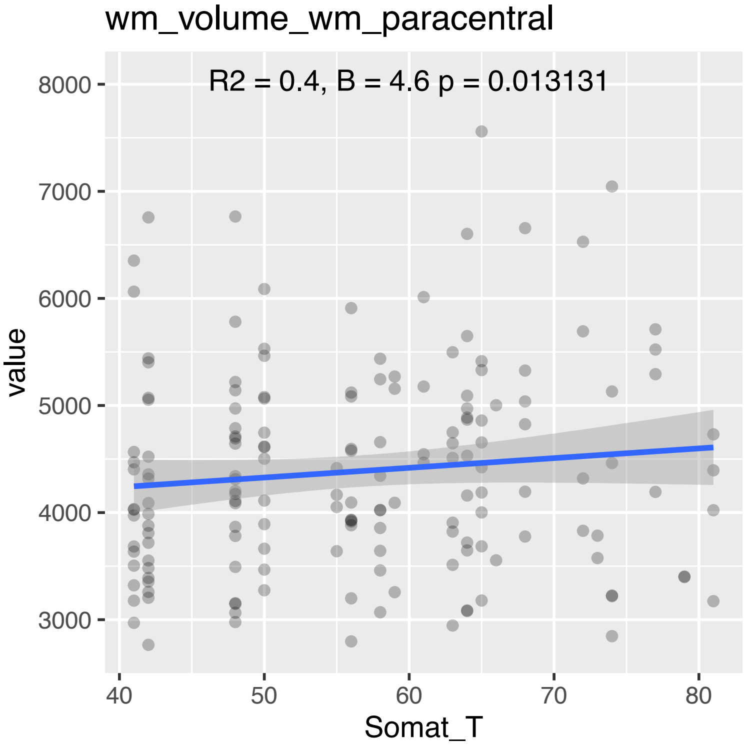
wm_volume_wm_paracentral
R2 = 0.4, B = 4.6 p = 0.013131
value
8000
7000
6000
5000
4000
3000
40
50
60
70
80
Somat_T

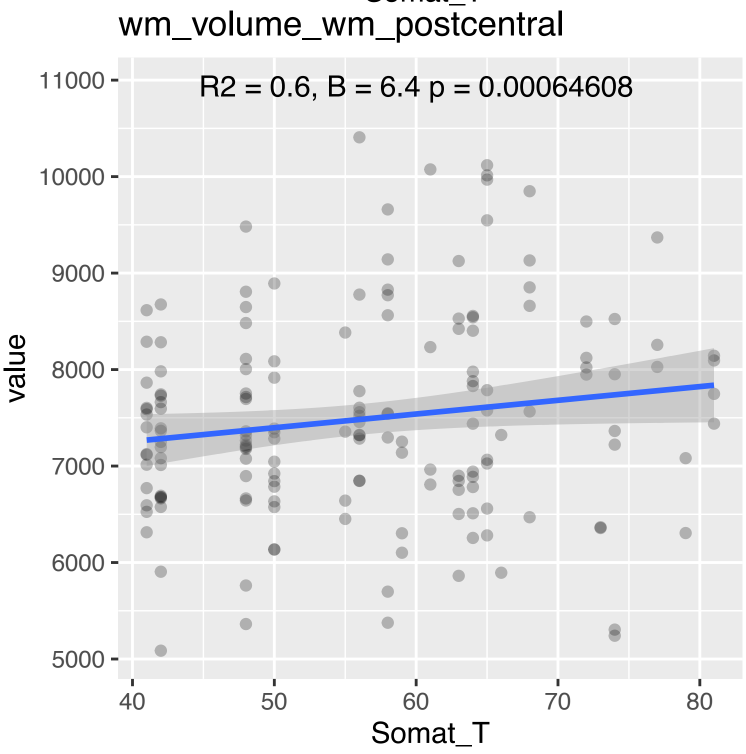
wm_volume_wm_postcentral
R2 = 0.6, B = 6.4 p = 0.00064608
value
11000
10000
9000
8000
7000
6000
5000
40
50
60
70
80
Somat_T

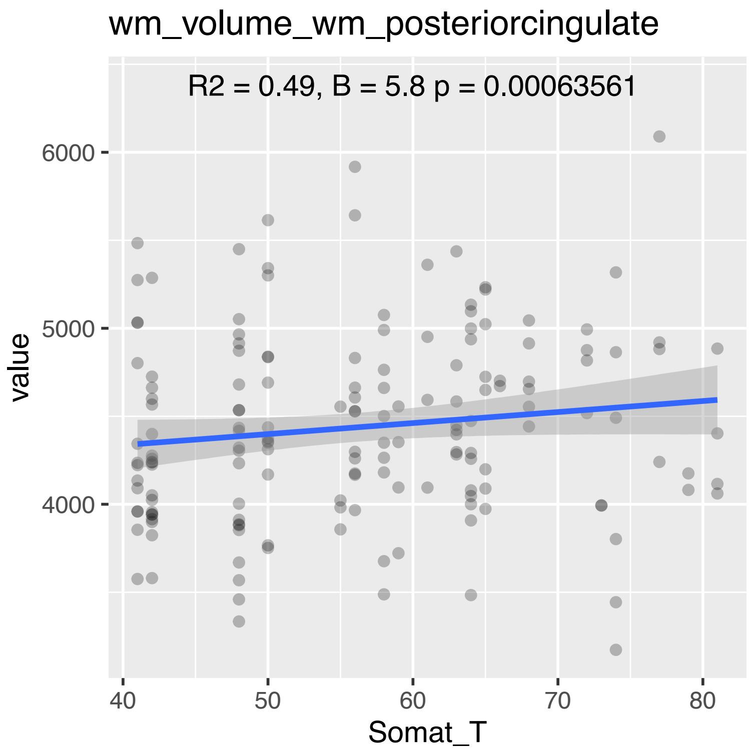
wm_volume_wm_posteriorcingulate
R2 = 0.49, B = 5.8 p = 0.00063561
6000
5000
4000
value
40
50
60
70
80
Somat_T

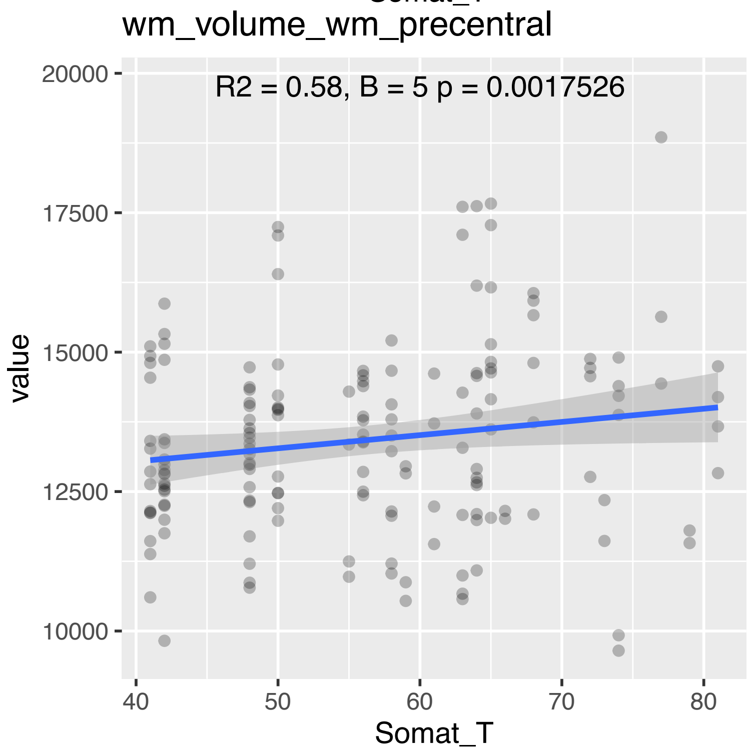
wm_volume_wm_precentral
R2 = 0.58, B = 5 p = 0.0017526
20000
17500
15000
12500
10000
value
40
50
60
70
80
Somat_T

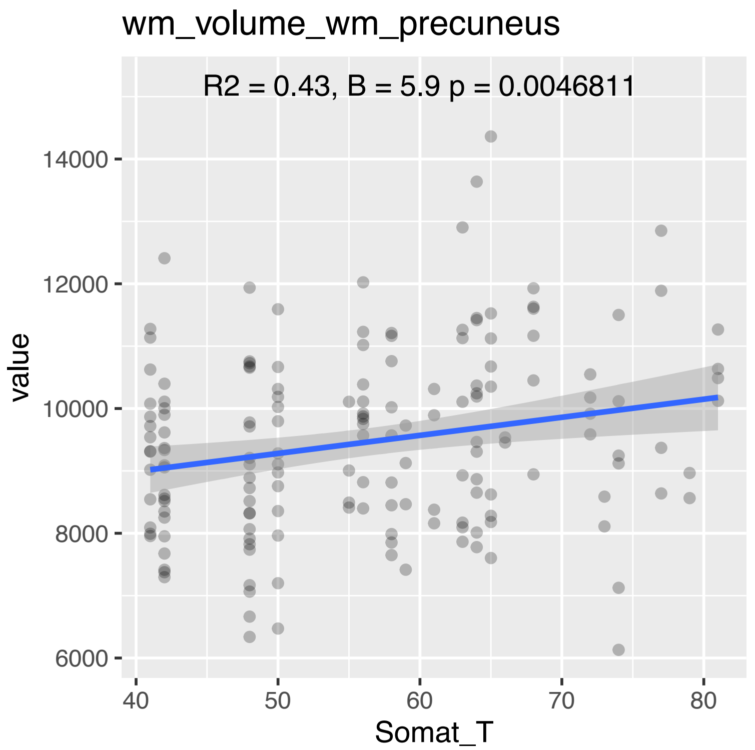
wm_volume_wm_precuneus
R2 = 0.43, B = 5.9 p = 0.0046811
value
14000
12000
10000
8000
6000
40
50
60
70
80
Somat_T

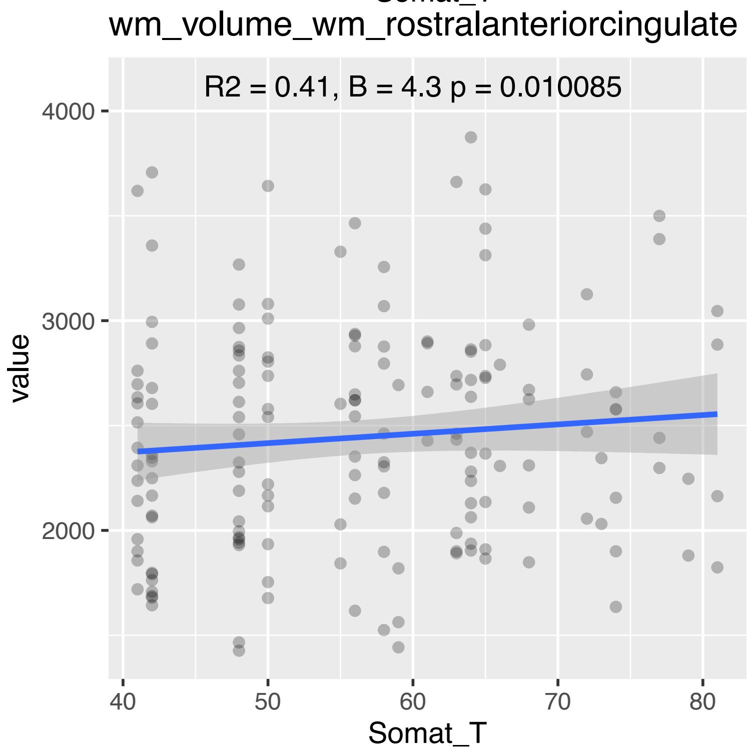
wm_volume_wm_rostralanteriorcingulate
R2 = 0.41, B = 4.3 p = 0.010085
value
4000
3000
2000
40
50
60
70
80
Somat_T

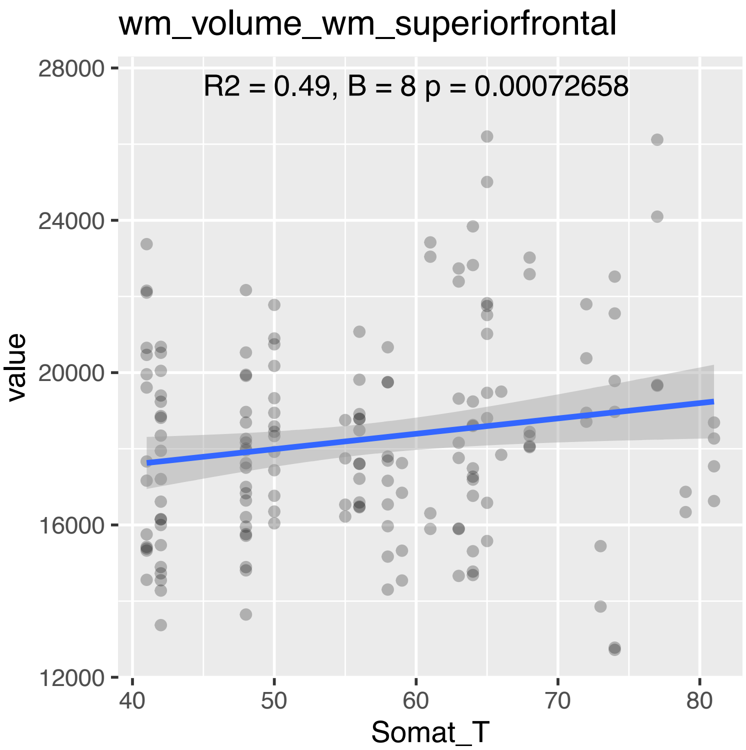
wm_volume_wm_superiorfrontal
R2 = 0.49, B = 8 p = 0.00072658
value
28000
24000
20000
16000
12000
40
50
60
70
80
Somat_T

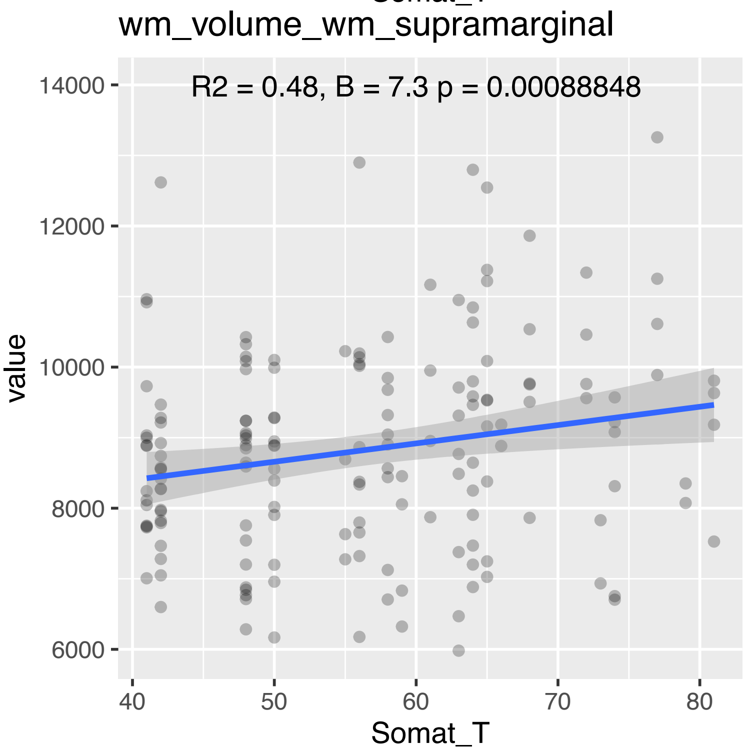
wm_volume_wm_supramarginal
R2 = 0.48, B = 7.3 p = 0.00088848
value
14000
12000
10000
8000
6000
40
50
60
70
80
Somat_T

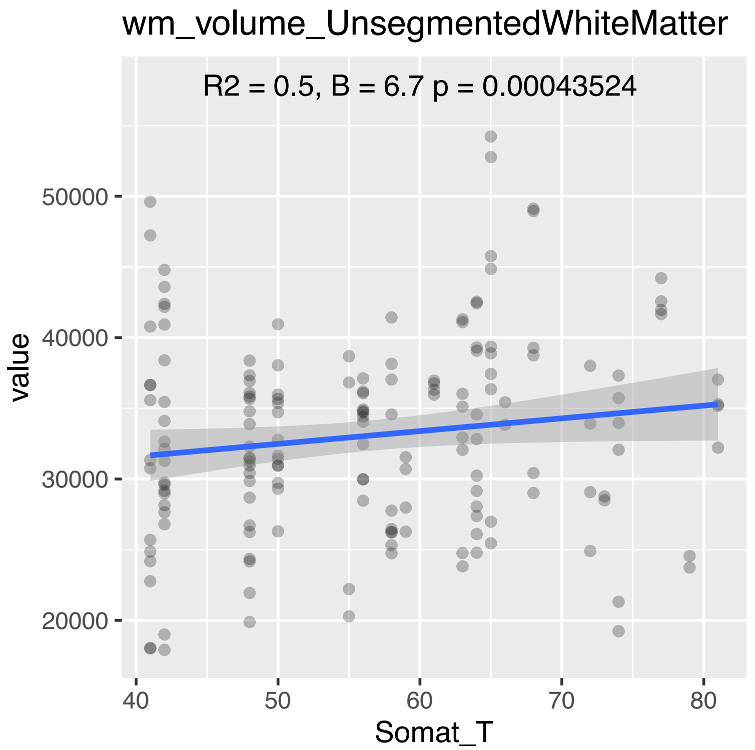
wm_volume_UnsegmentedWhiteMatter
R2 = 0.5, B = 6.7 p = 0.00043524
50000
40000
30000
20000
value
40
50
60
70
80
Somat_T

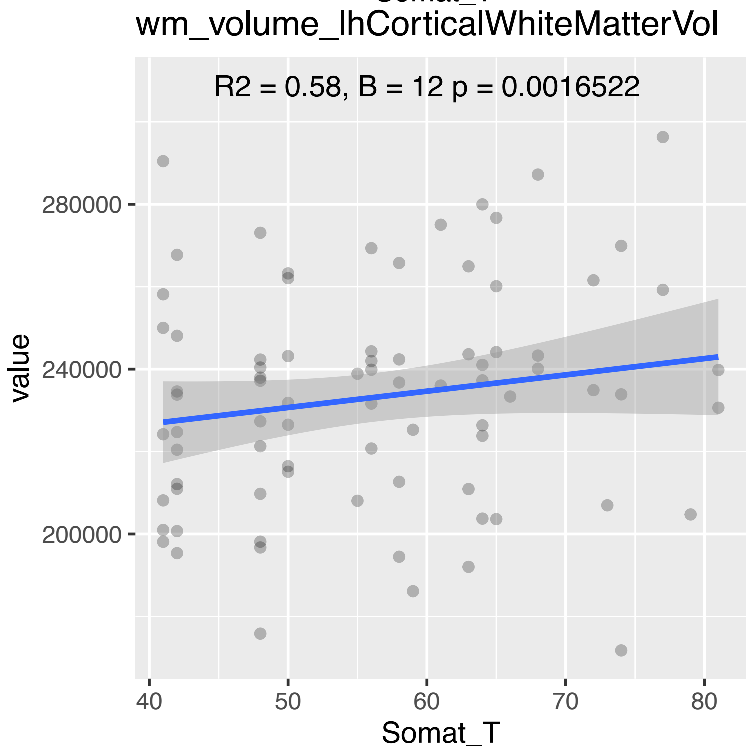
wm_volume_lhCorticalWhiteMatterVol
R2 = 0.58, B = 12 p = 0.0016522
280000
240000
200000
value
40
50
60
70
80
Somat_T

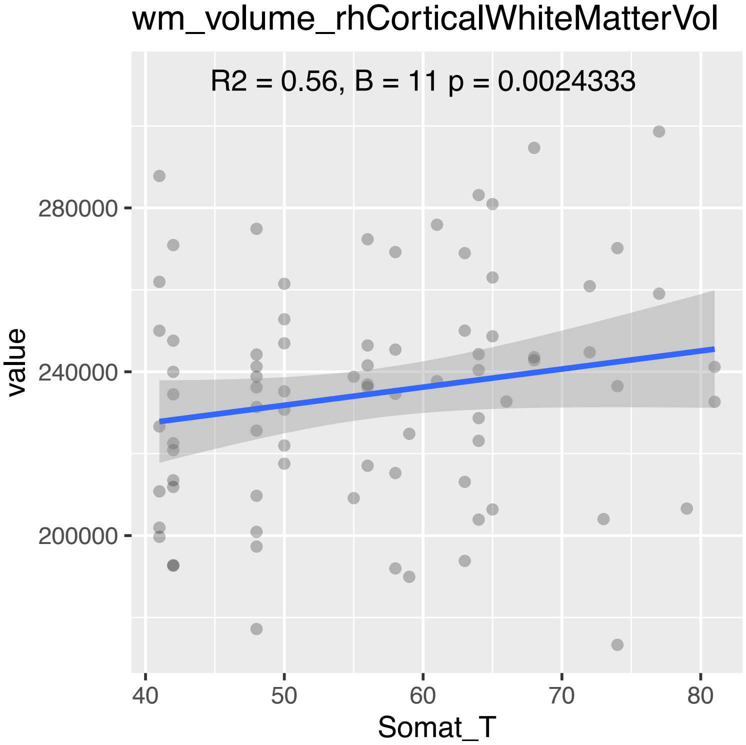
wm_volume_rhCorticalWhiteMatterVol
R2 = 0.56, B = 11 p = 0.0024333
280000
240000
200000
value
40
50
60
70
80
Somat_T

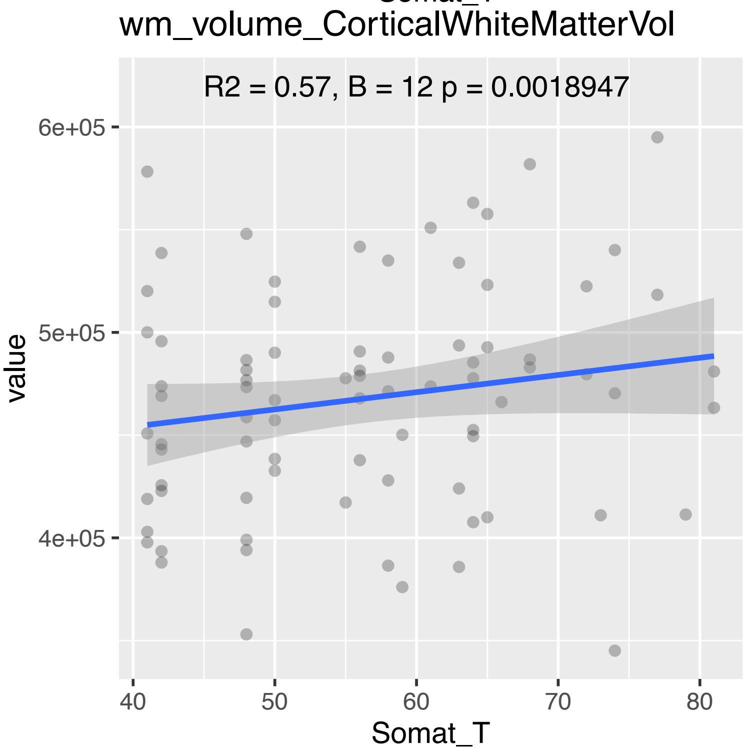
wm_volume_CorticalWhiteMatterVol
R2 = 0.57, B = 12 p = 0.0018947
6e+05
5e+05
4e+05
value
40
50
60
70
80
Somat_T

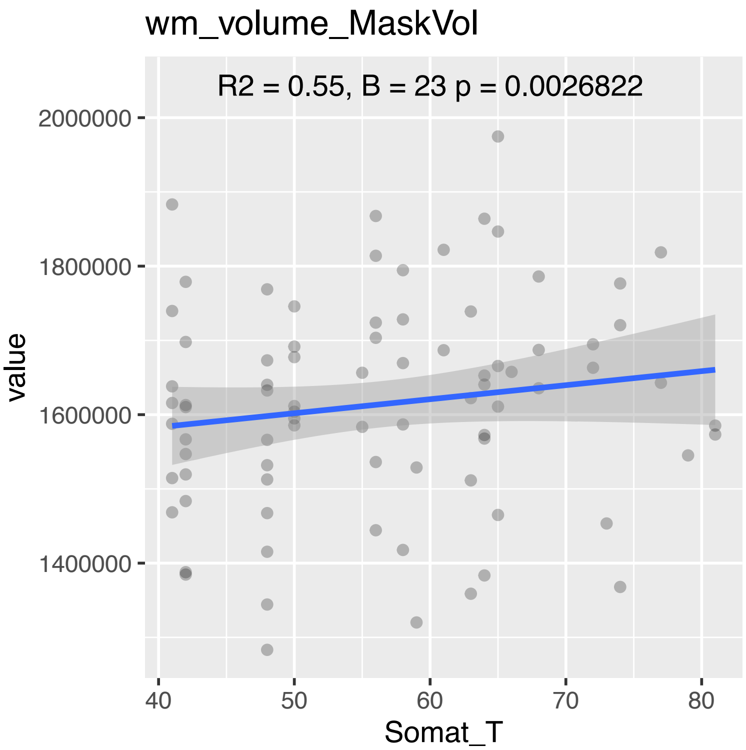
wm_volume_MaskVol
R2 = 0.55, B = 23 p = 0.0026822
value
2000000
1800000
1600000
1400000
40
50
60
70
80
Somat_T

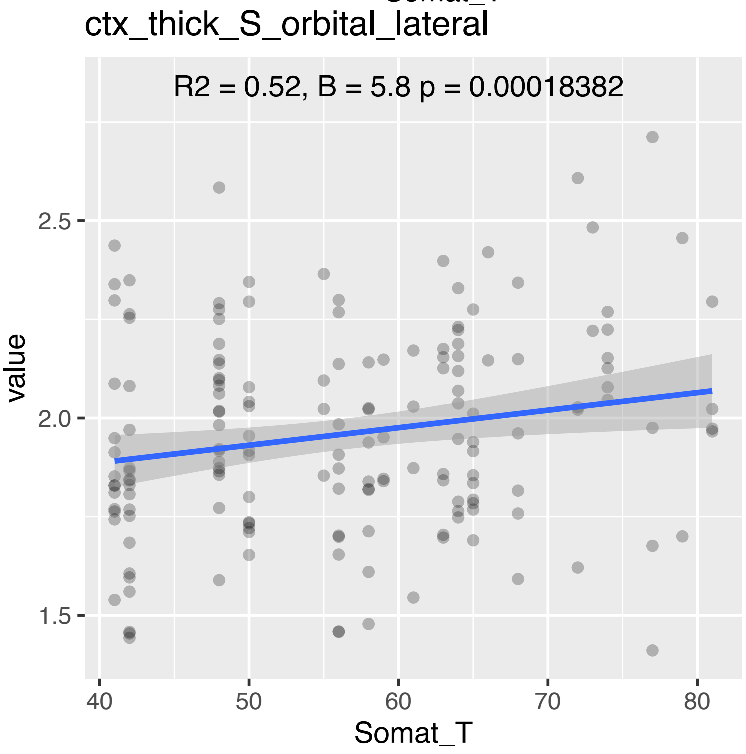
ctx_thick_S_orbital_lateral
R2 = 0.52, B = 5.8 p = 0.00018382
value
2.5
2.0
1.5
40
50
60
70
80
Somat_T

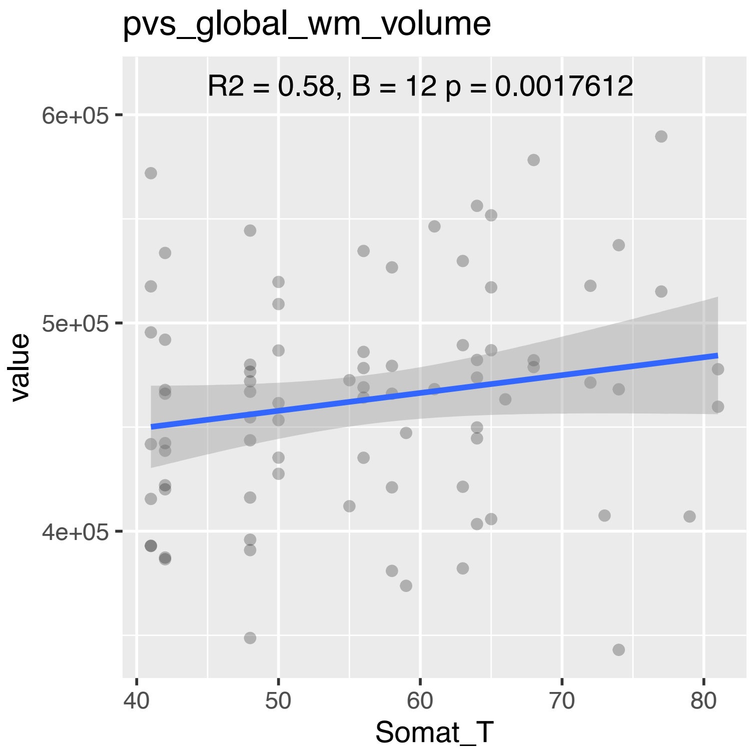
pvs_global_wm_volume
R2 = 0.58, B = 12 p = 0.0017612
6e+05
5e+05
4e+05
value
40
50
60
70
80
Somat_T

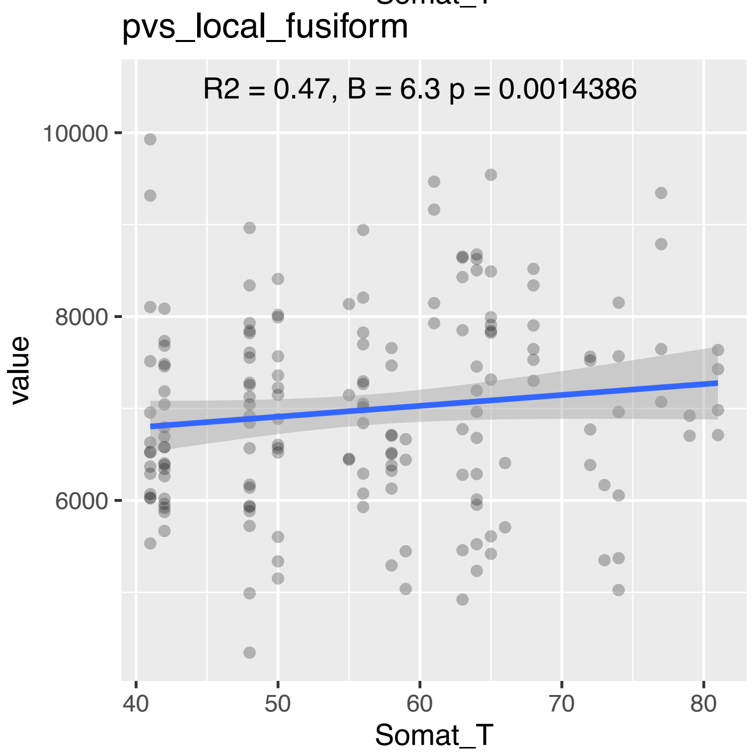
pvs_local_fusiform
R2 = 0.47, B = 6.3 p = 0.0014386
10000
8000
6000
value
40
50
60
70
80
Somat_T

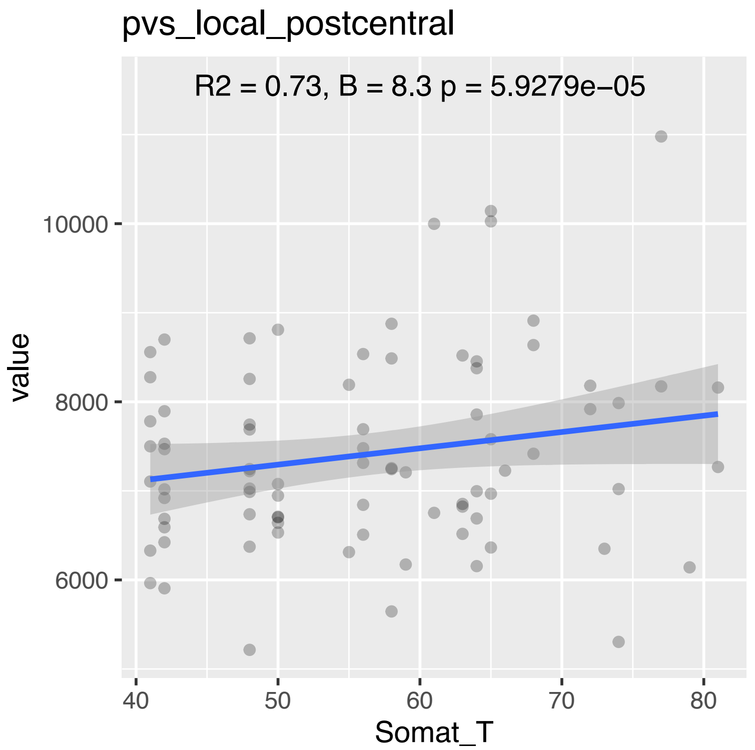
pvs_local_postcentral
R2 = 0.73, B = 8.3 p = 5.9279e−05
value
10000
8000
6000
40
50
60
70
80
Somat_T

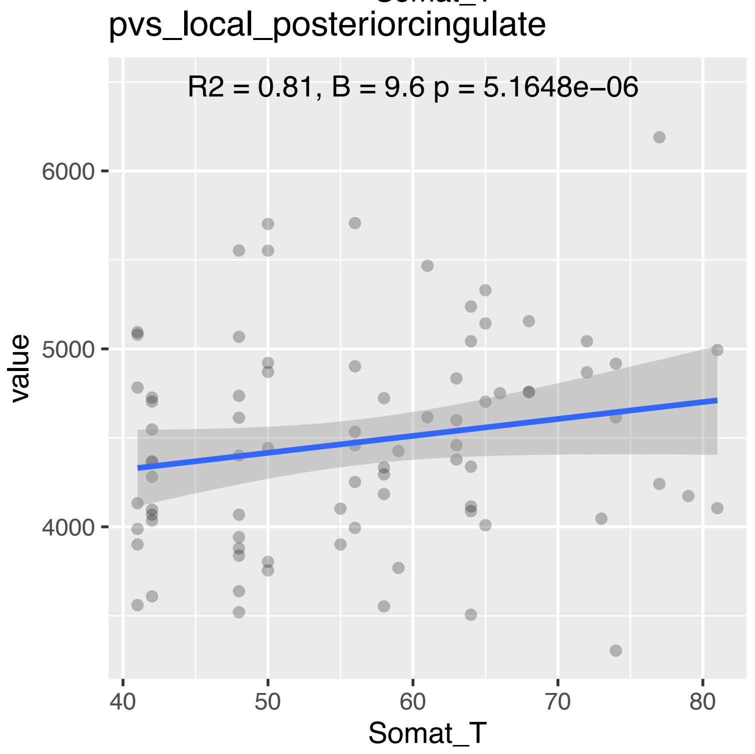
pvs_local_posteriorcingulate
R2 = 0.81, B = 9.6 p = 5.1648e−06
value
6000
5000
4000
40
50
60
70
80
Somat_T

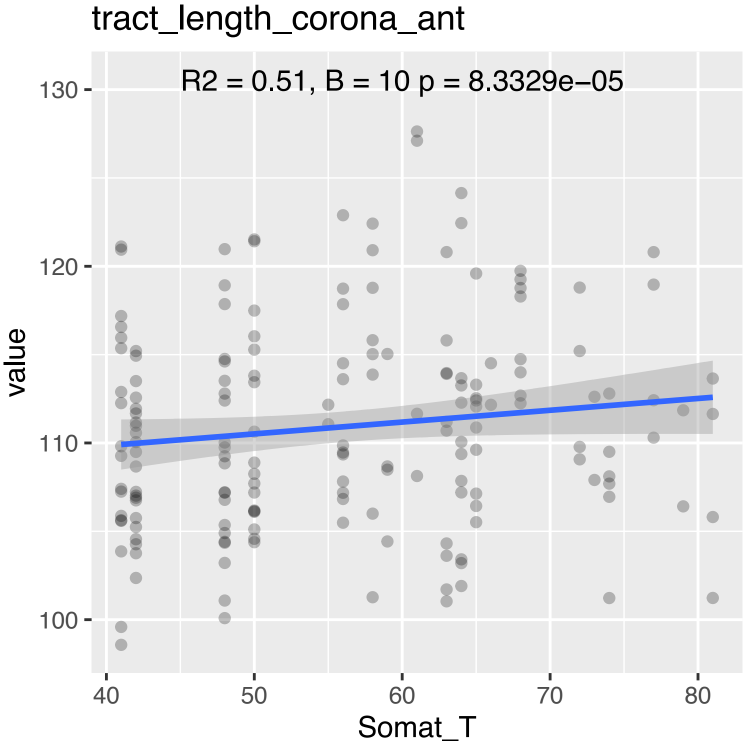
tract_length_corona_ant
R2 = 0.51, B = 10 p = 8.3329e−05
value
Somat_T
130
120
110
100
40
50
60
70
80

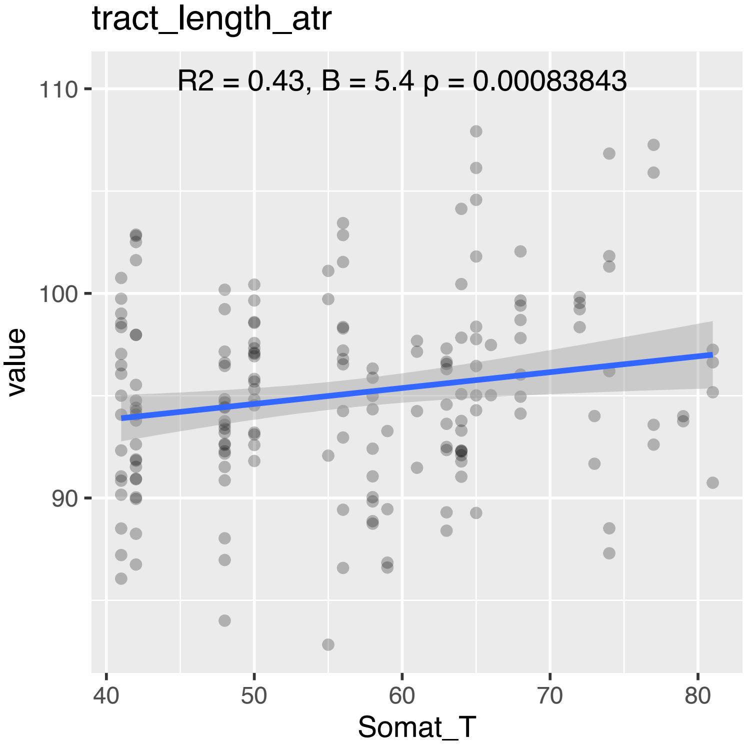
tract_length_atr
R2 = 0.43, B = 5.4 p = 0.00083843
value
Somat_T
110
100
90
40
50
60
70
80

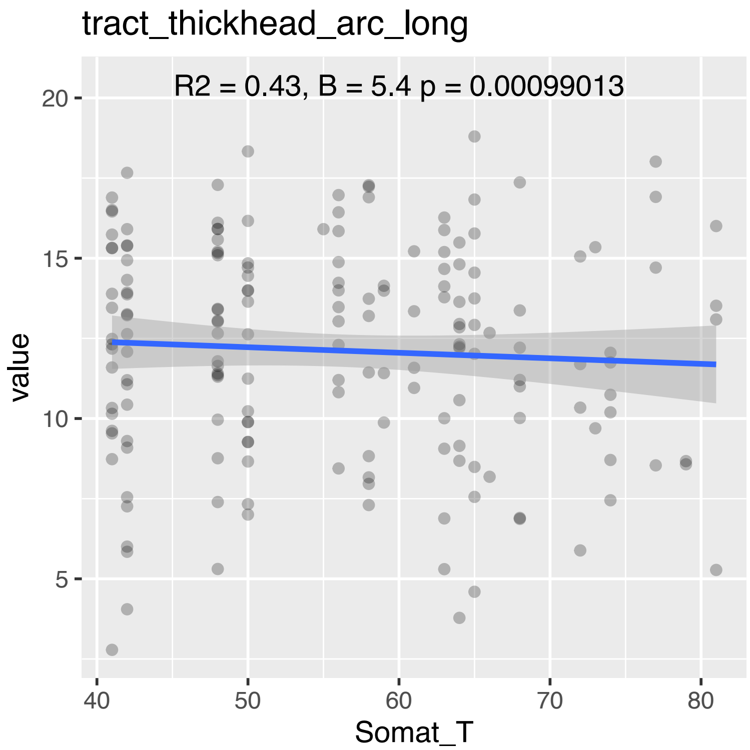
tract_thickhead_arc_long
R2 = 0.43, B = 5.4 p = 0.00099013
value
20
15
10
5
40
50
60
70
80
Somat_T

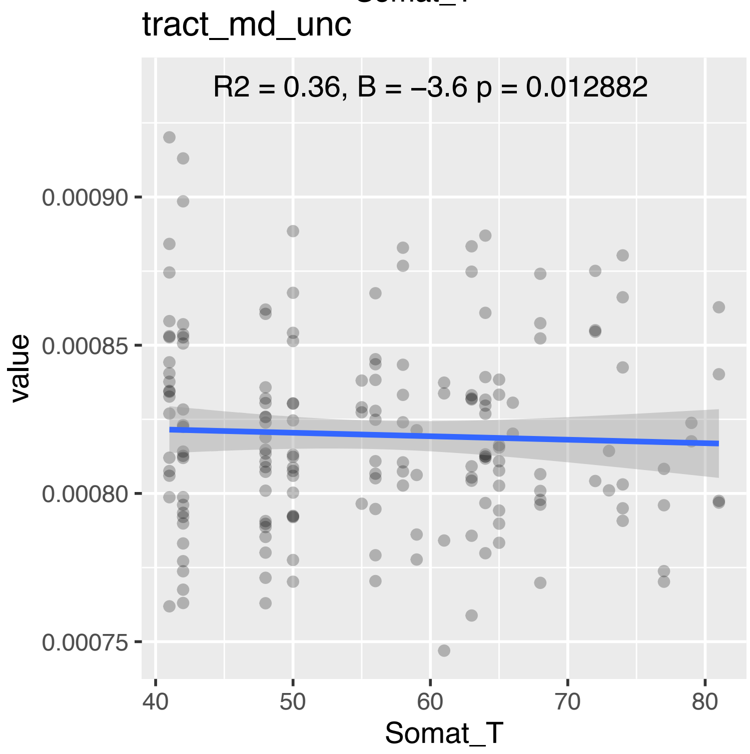
tract_md_unc
R2 = 0.36, B = −3.6 p = 0.012882
value
0.00090
0.00085
0.00080
0.00075
40
50
60
70
80
Somat_T

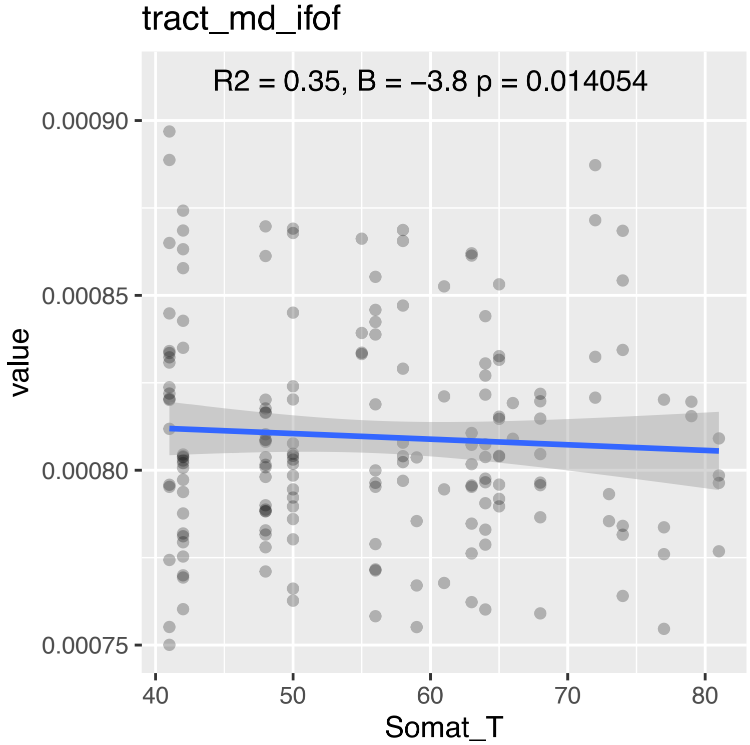
tract_md_ifof
R2 = 0.35, B = −3.8 p = 0.014054
value
0.00090
0.00085
0.00080
0.00075
40
50
60
70
80
Somat_T

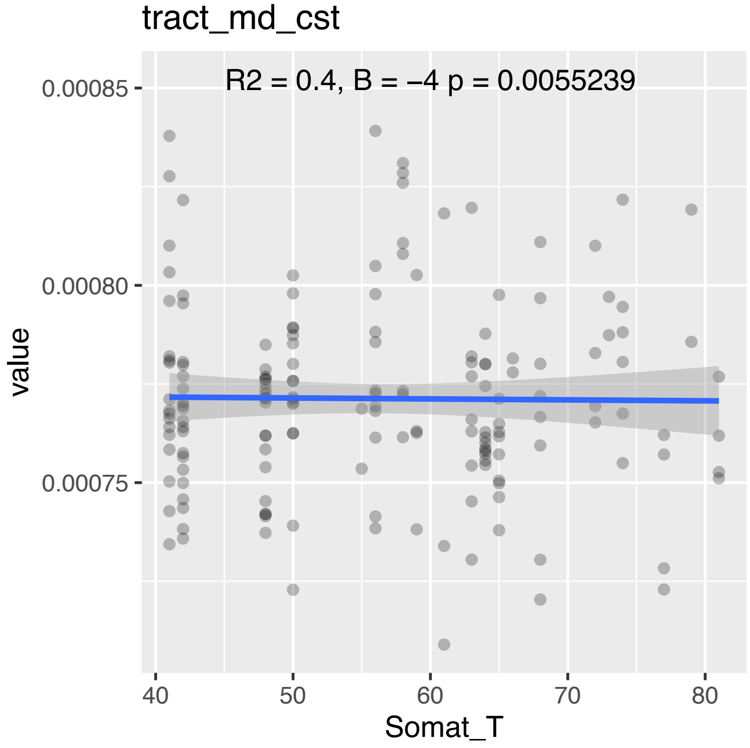
tract_md_cst
R2 = 0.4, B = −4 p = 0.0055239
value
0.00085
0.00080
0.00075
40
50
60
70
80
Somat_T

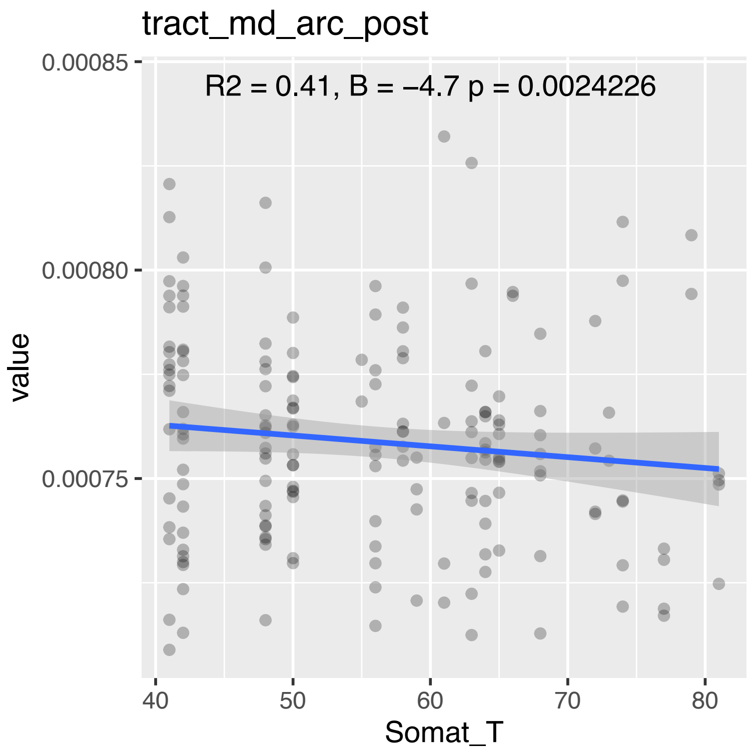
tract_md_arc_post
R2 = 0.41, B = −4.7 p = 0.0024226
0.00085
0.00080
0.00075
value
40
50
60
70
80
Somat_T

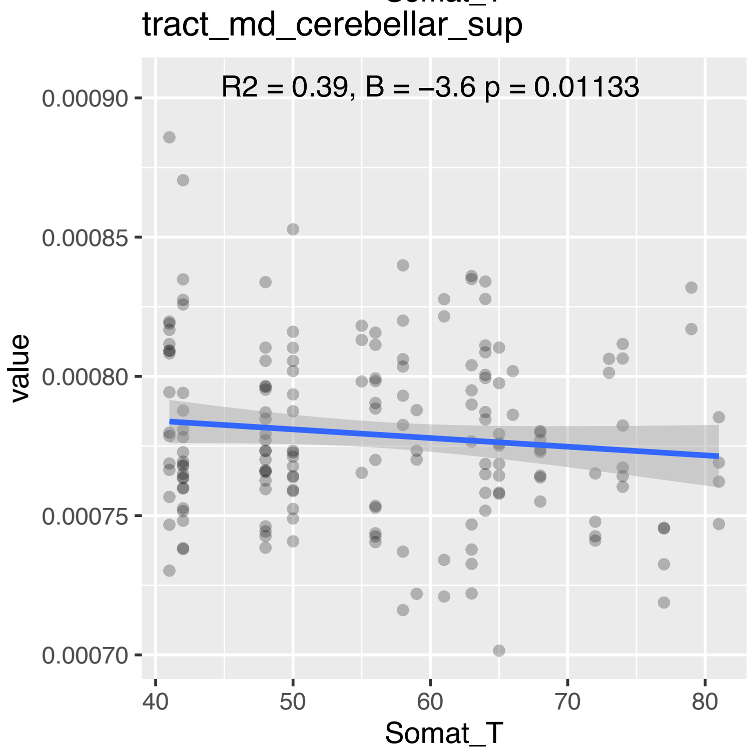
tract_md_cerebellar_sup
R2 = 0.39, B = −3.6 p = 0.01133
0.00090
0.00085
0.00080
0.00075
0.00070
value
40
50
60
70
80
Somat_T

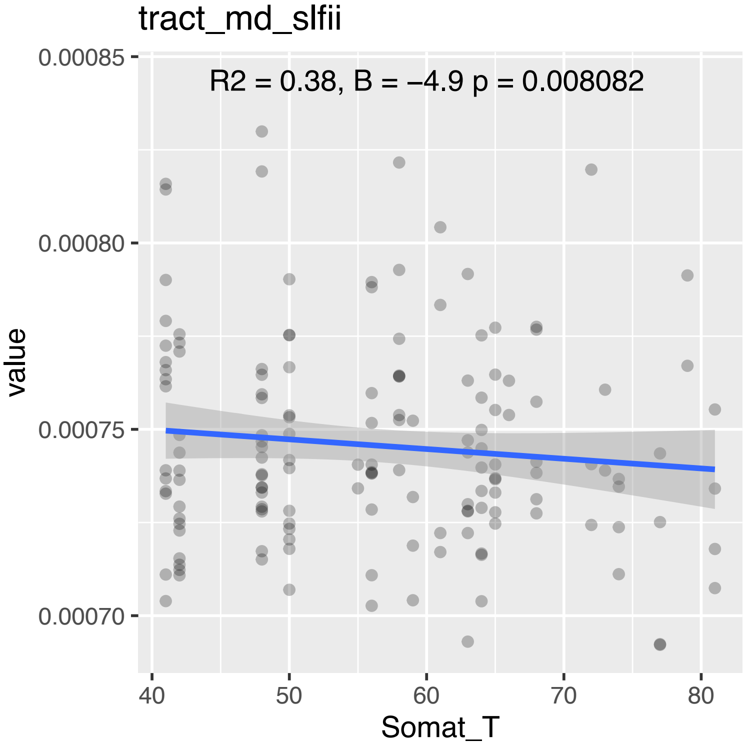
tract_md_slfii
R2 = 0.38, B = −4.9 p = 0.008082
0.00085
0.00080
0.00075
0.00070
value
40
50
60
70
80
Somat_T

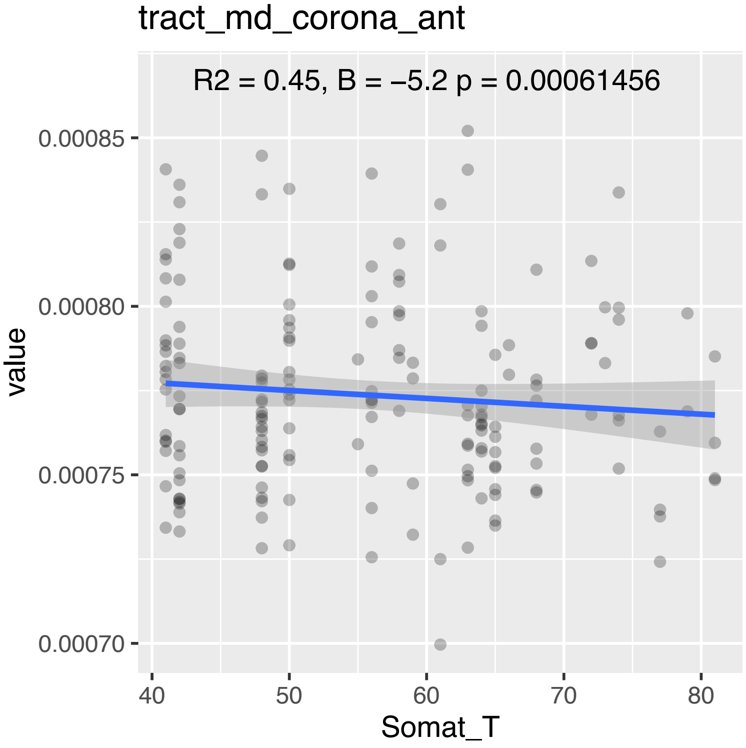
tract_md_corona_ant
R2 = 0.45, B = −5.2 p = 0.00061456
0.00085
0.00080
0.00075
0.00070
value
40
50
60
70
80
Somat_T

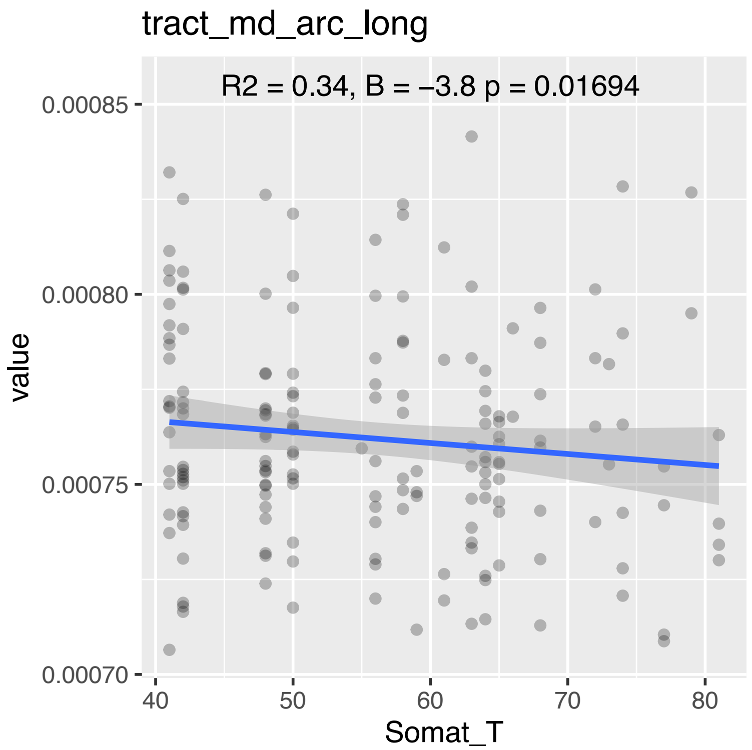
tract_md_arc_long
R2 = 0.34, B = −3.8 p = 0.01694
0.00085
0.00080
0.00075
0.00070
value
40
50
60
70
80
Somat_T

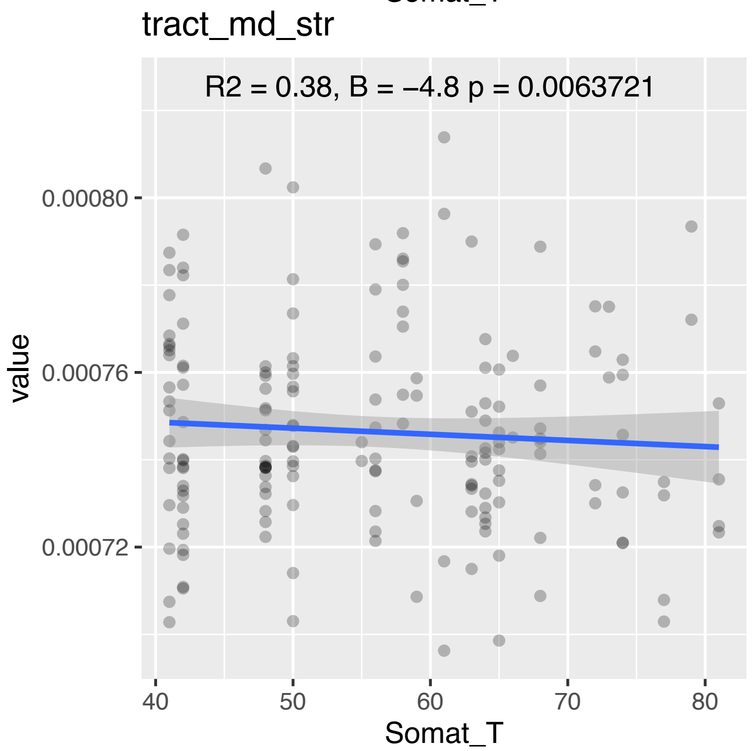
tract_md_str
R2 = 0.38, B = −4.8 p = 0.0063721
0.00080
0.00076
0.00072
value
40
50
60
70
80
Somat_T

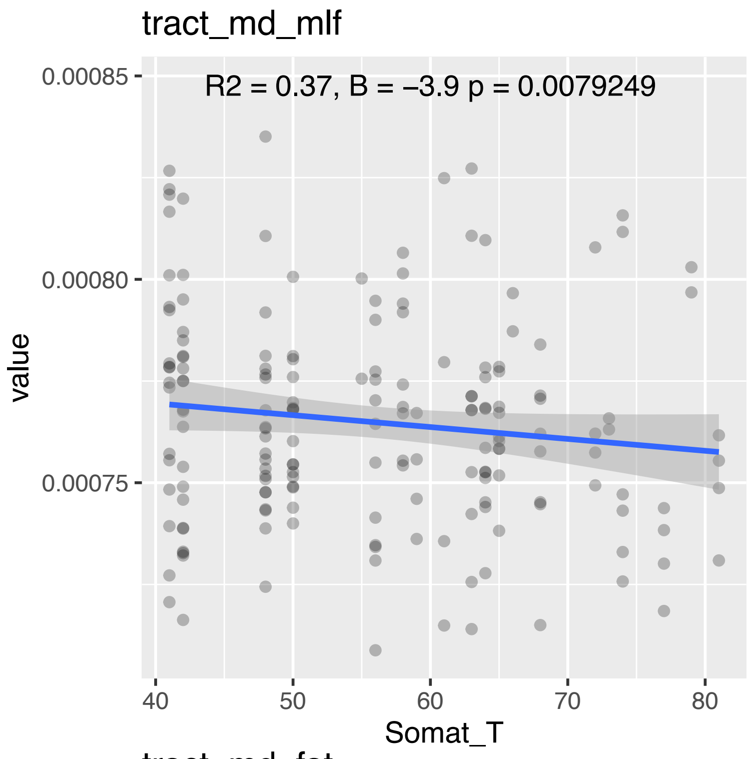
tract_md_mlf
R2 = 0.37, B = −3.9 p = 0.0079249
0.00085
0.00080
0.00075
value
40
50
60
70
80
Somat_T

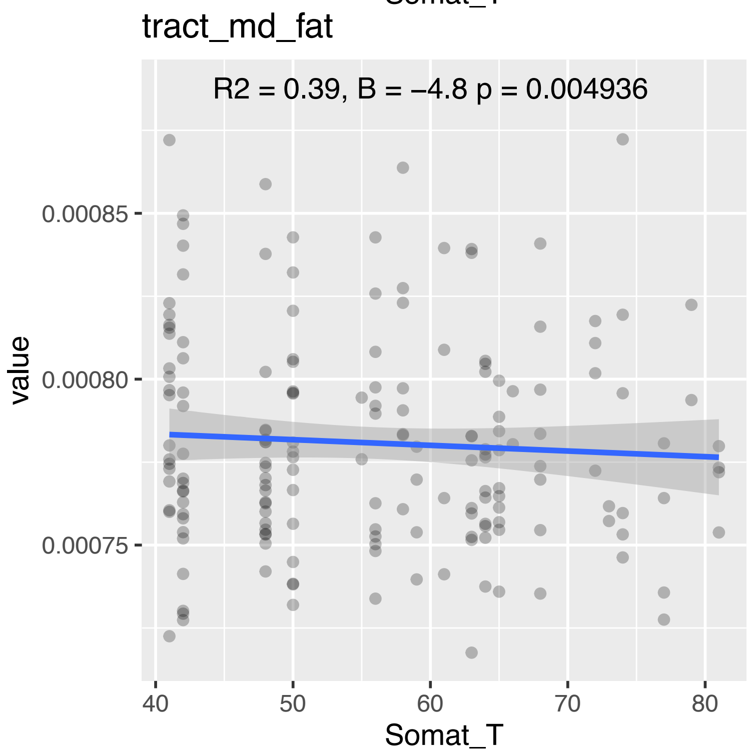
tract_md_fat
R2 = 0.39, B = −4.8 p = 0.004936
0.00085
0.00080
0.00075
value
40
50
60
70
80
Somat_T

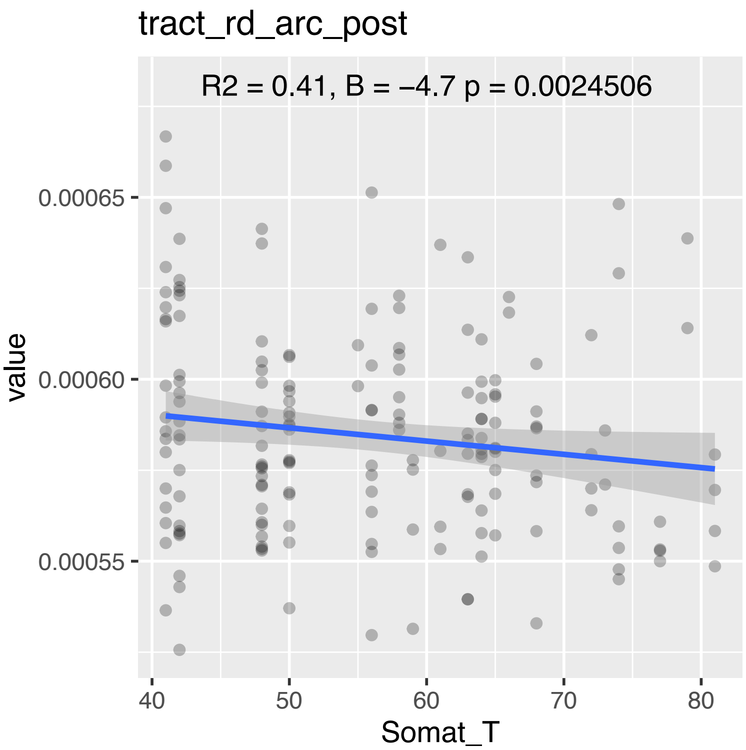
tract_rd_arc_post
R2 = 0.41, B = −4.7 p = 0.0024506
value
0.00065
0.00060
0.00055
40
50
60
70
80
Somat_T

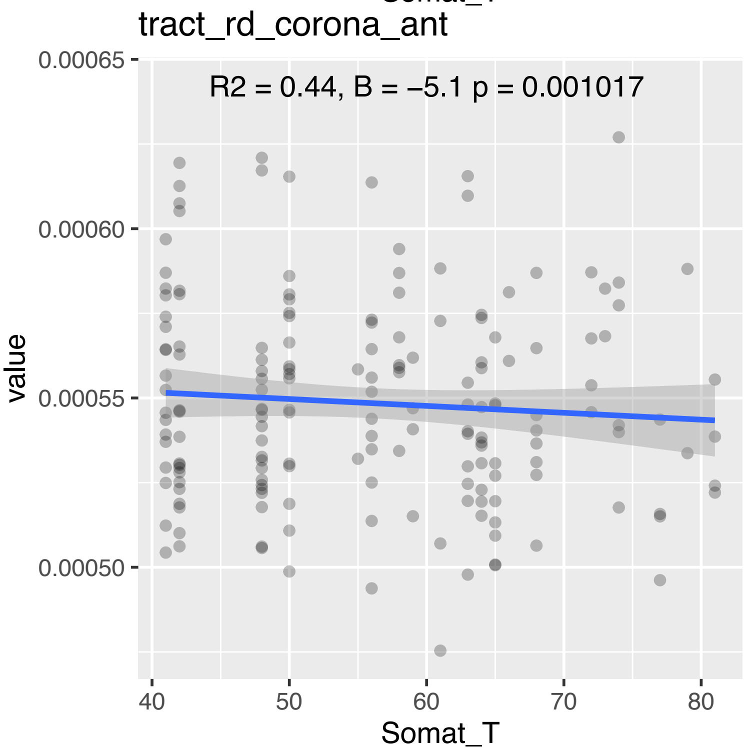
tract_rd_corona_ant
R2 = 0.44, B = −5.1 p = 0.001017
value
0.00065
0.00060
0.00055
0.00050
40
50
60
70
80
Somat_T

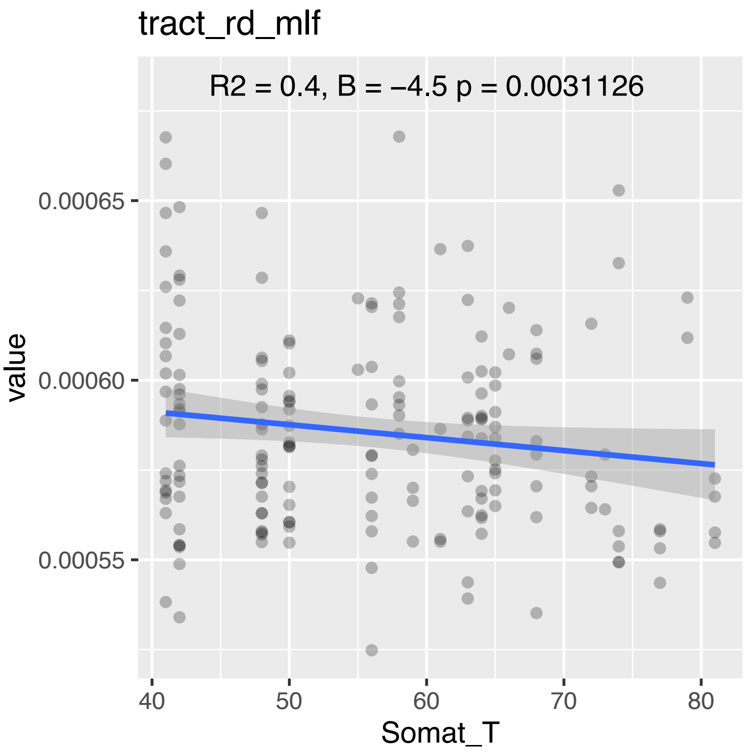

Figure S5: Scatterplots indicating statistically significant relationships between somatization symptoms and brain regions (here hemispheres are analyzed together)

2.2. Hemispheres separated

2.2.1. Depression and anxiety lateralization

For depression and anxiety symptoms, there were no statistically significant associations when considering the two hemispheres together, after FDR correction for multiple comparisons ($p>0.05$).

2.2.2. Somatization

Taking into account hemispheric lateralization, somatization symptoms were positively associated to the volume of the left thalamus, hippocampus, ventral diencephalon (formed by hypothalamus, mammillary body, subthalamic nuclei, substantia nigra, red nucleus, lateral geniculate nucleus, and medial geniculate nucleus). In the right hemisphere, significant positive relationships were seen with the volume of hypothalamus, amygdala, the white matter of posterior cingulate, and the volume of subcortical structures. Globally, volumes of total white matter and subcortical grey matter were affected by somatization.

For the PVS results, local significant positive associations were found in the left postcentral and right posterior cingulate. Regarding white matter tracts, there was a positive relationship between the thickness of the right arcuate fasciculus and the left superior thalamic radiation.
Below scatterplots of significant relationships are shown.

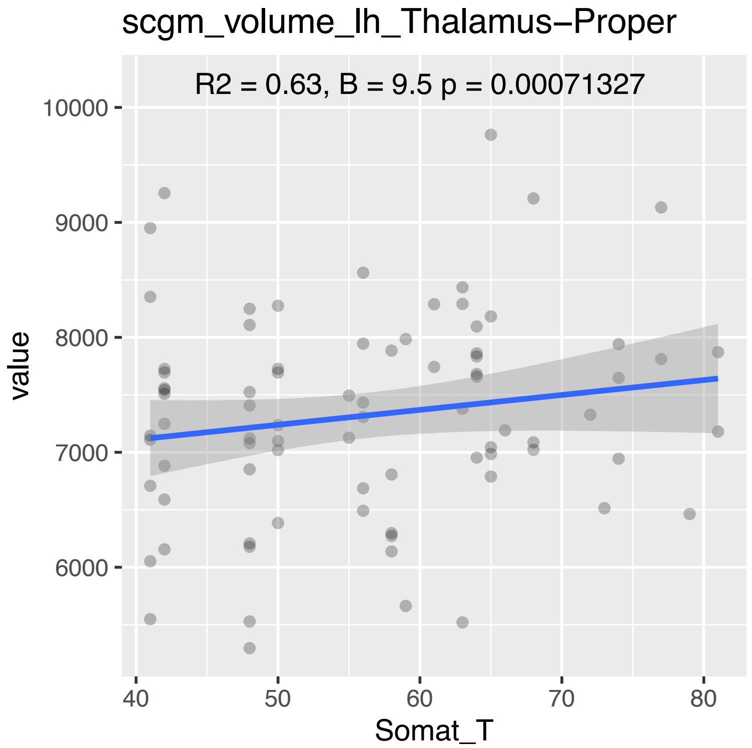

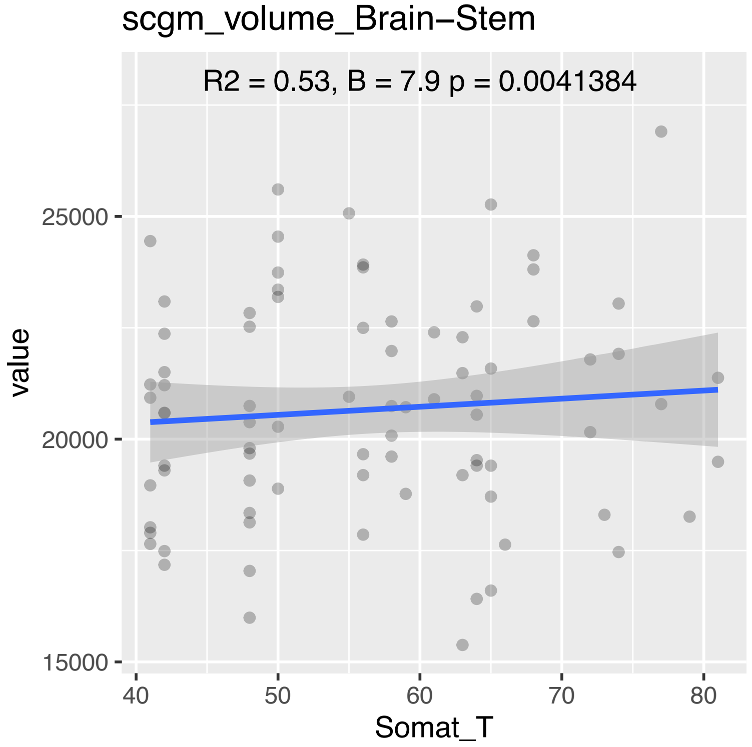

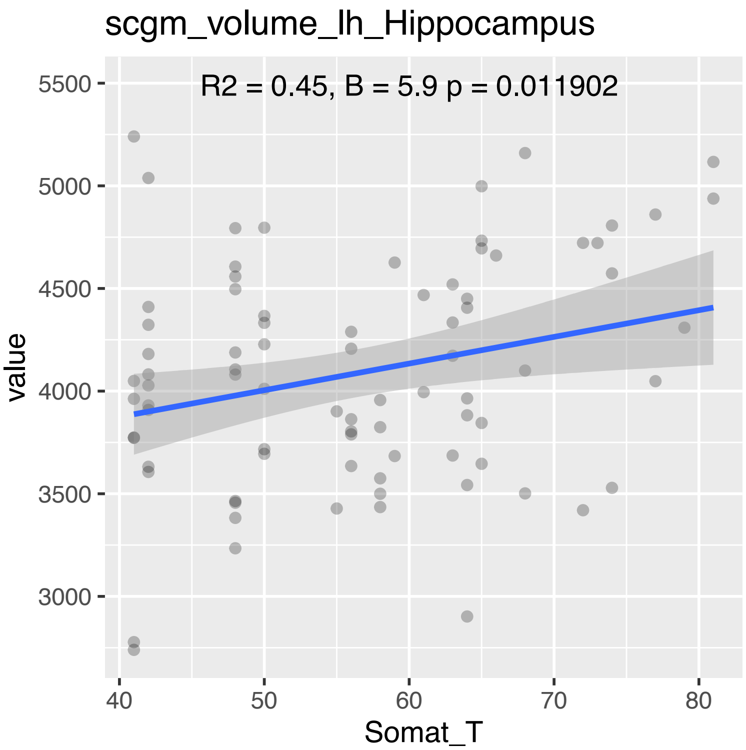
scgm_volume_lh_Hippocampus
R2 = 0.45, B = 5.9 p = 0.011902
value
Somat_T

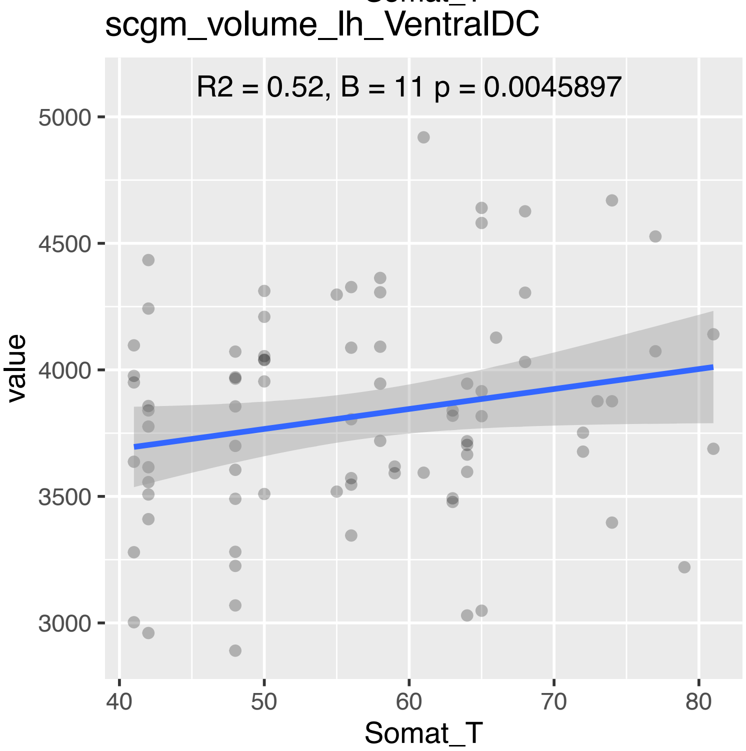
scgm_volume_lh_VentralDC
R2 = 0.52, B = 11 p = 0.0045897
value
Somat_T

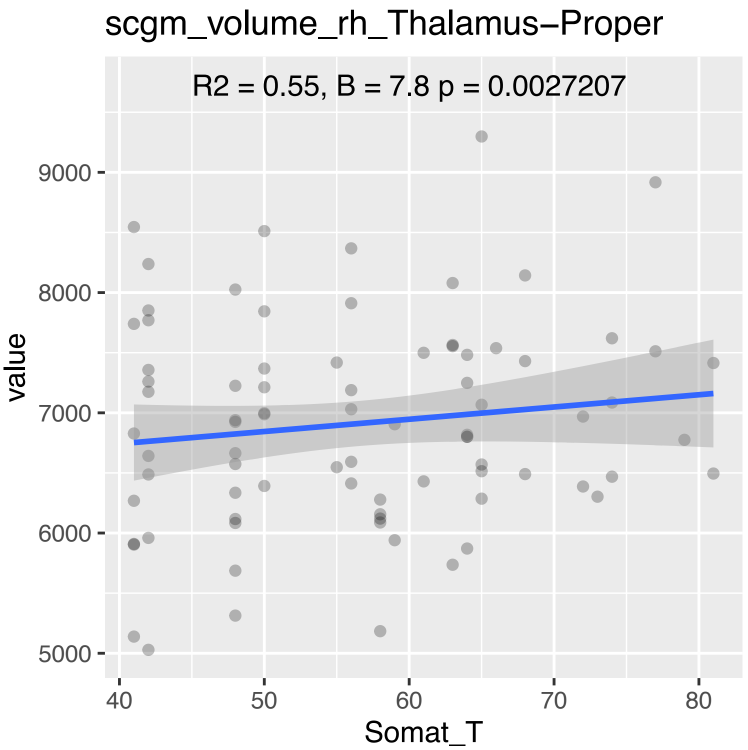
scgm_volume_rh_Thalamus−Proper
R2 = 0.55, B = 7.8 p = 0.0027207
value
9000
8000
7000
6000
5000
40
50
60
70
80
Somat_T

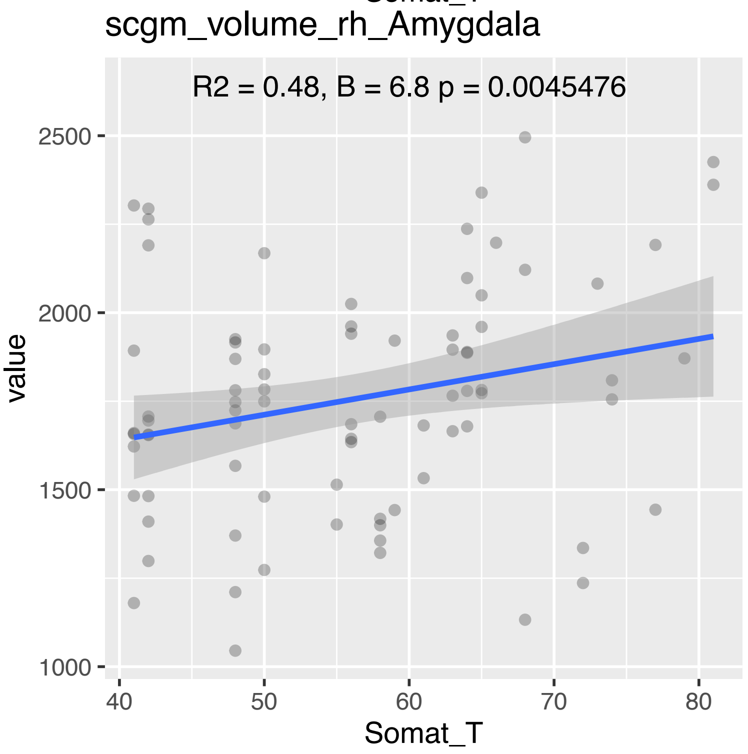
scgm_volume_rh_Amygdala
R2 = 0.48, B = 6.8 p = 0.0045476
value
2500
2000
1500
1000
40
50
60
70
80
Somat_T

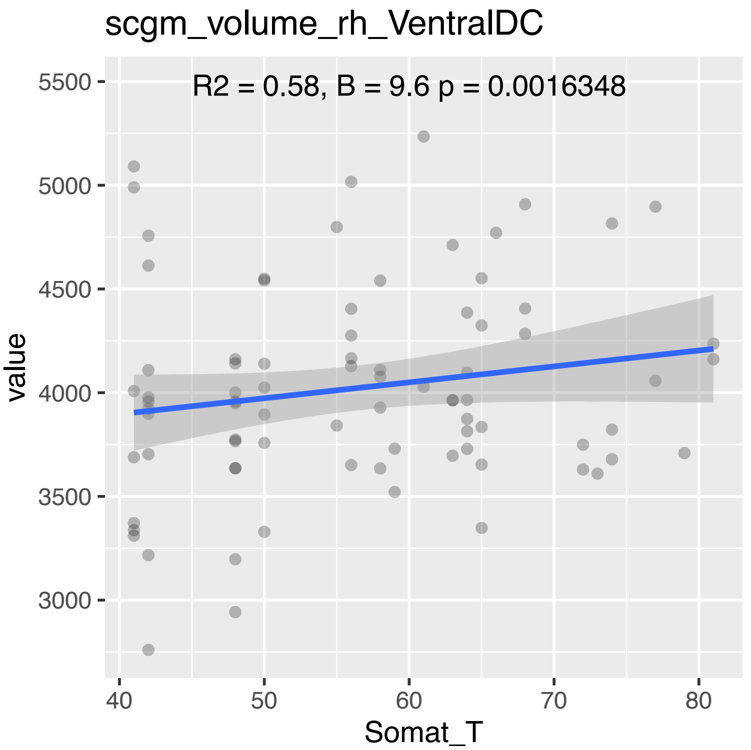
scgm_volume_rh_VentralDC
R2 = 0.58, B = 9.6 p = 0.0016348
value
5500
5000
4500
4000
3500
3000
40
50
60
70
80
Somat_T

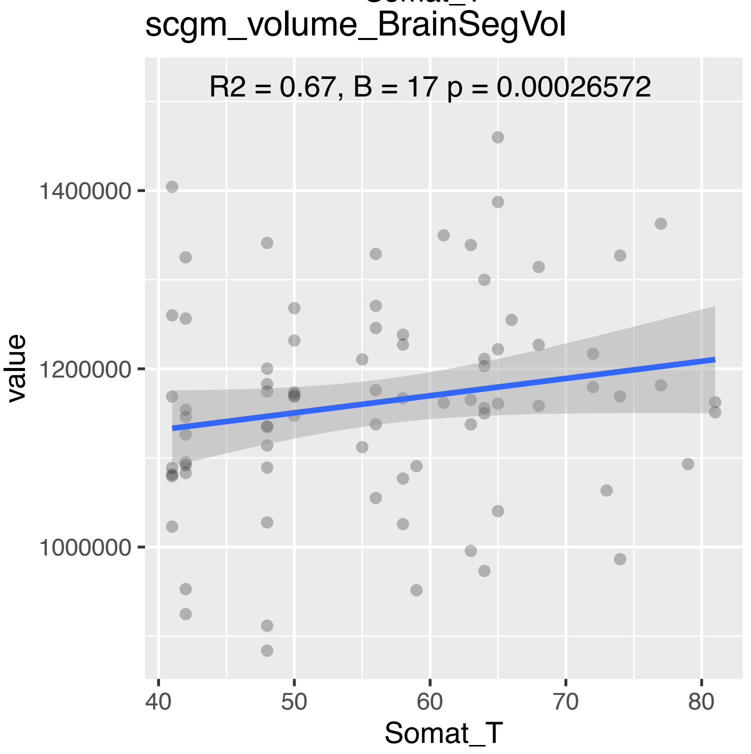
scgm_volume_BrainSegVol
R2 = 0.67, B = 17 p = 0.00026572
value
1400000
1200000
1000000
40
50
60
70
80
Somat_T

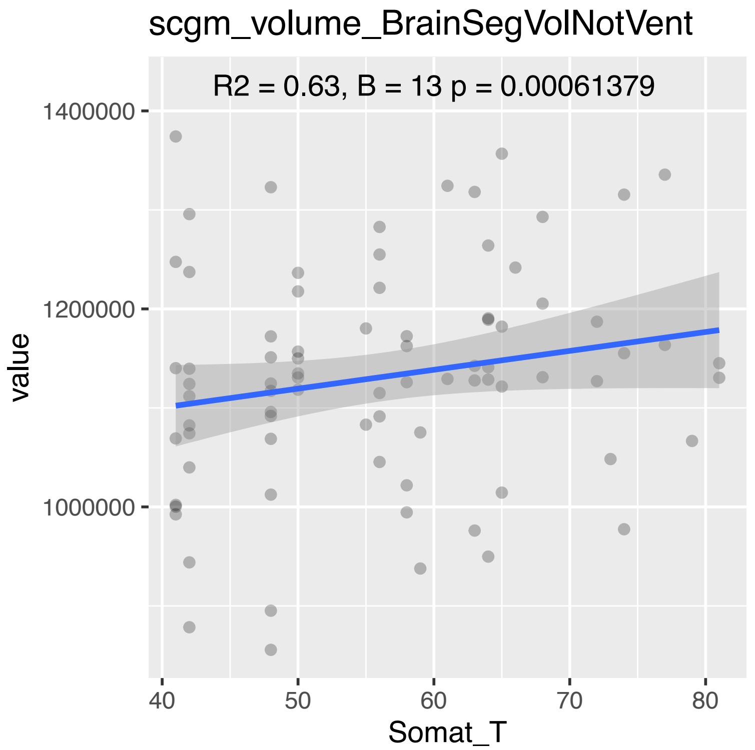
scgm_volume_BrainSegVolNotVent
R2 = 0.63, B = 13 p = 0.00061379
1400000
1200000
1000000
value
40
50
60
70
80
Somat_T

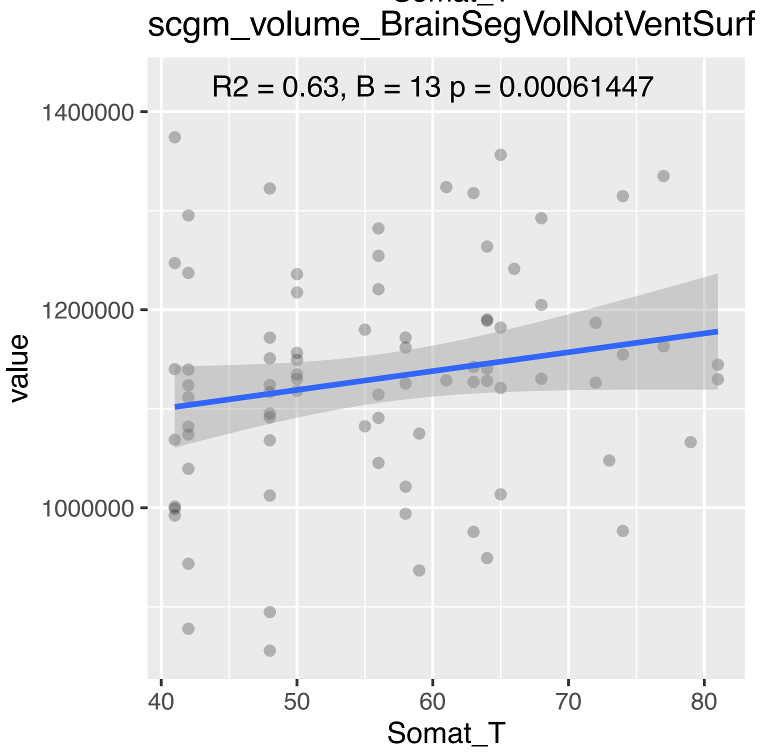
scgm_volume_BrainSegVolNotVentSurf
R2 = 0.63, B = 13 p = 0.00061447
1400000
1200000
1000000
value
40
50
60
70
80
Somat_T

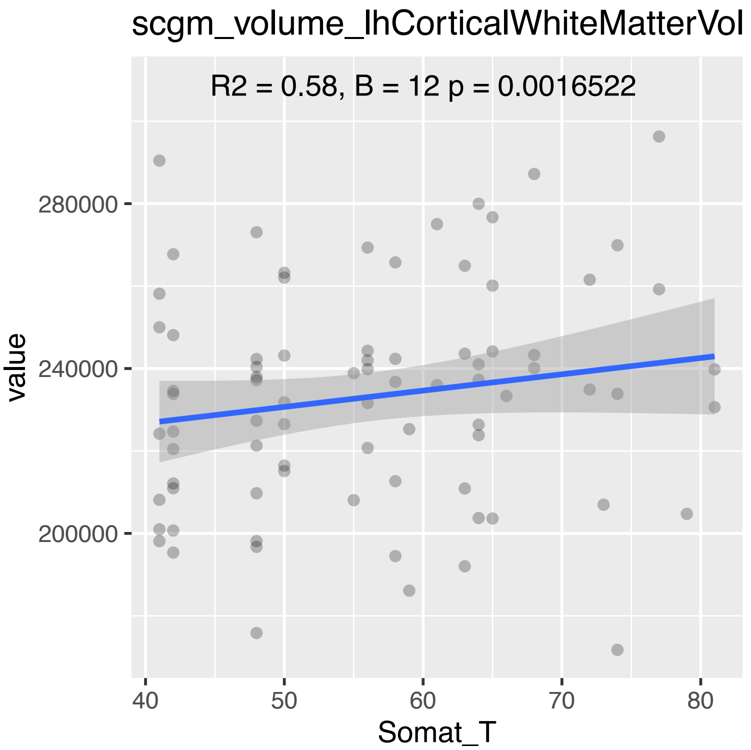
scgm_volume_lhCorticalWhiteMatterVol
R2 = 0.58, B = 12 p = 0.0016522
value
280000
240000
200000
40
50
60
70
80
Somat_T

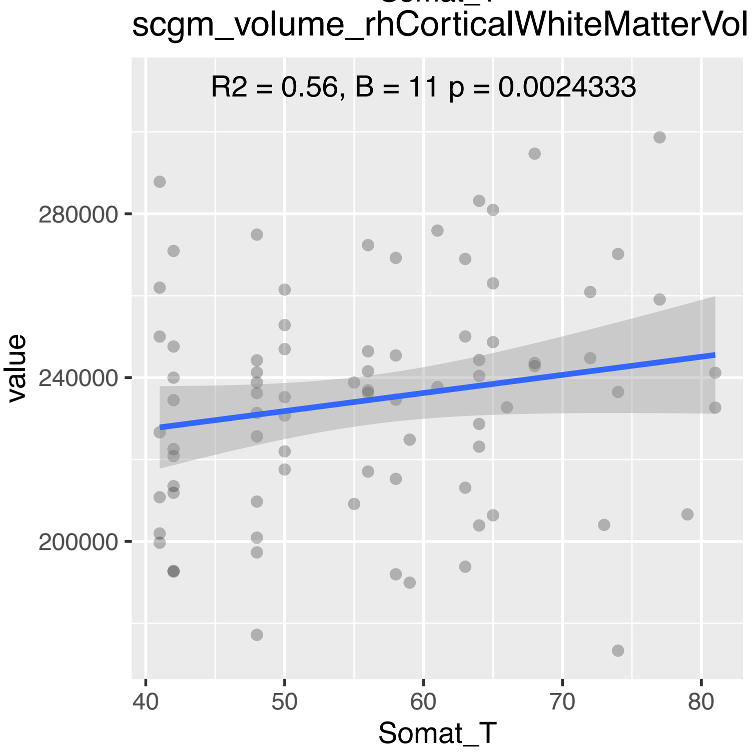
scgm_volume_rhCorticalWhiteMatterVol
R2 = 0.56, B = 11 p = 0.0024333
value
280000
240000
200000
40
50
60
70
80
Somat_T

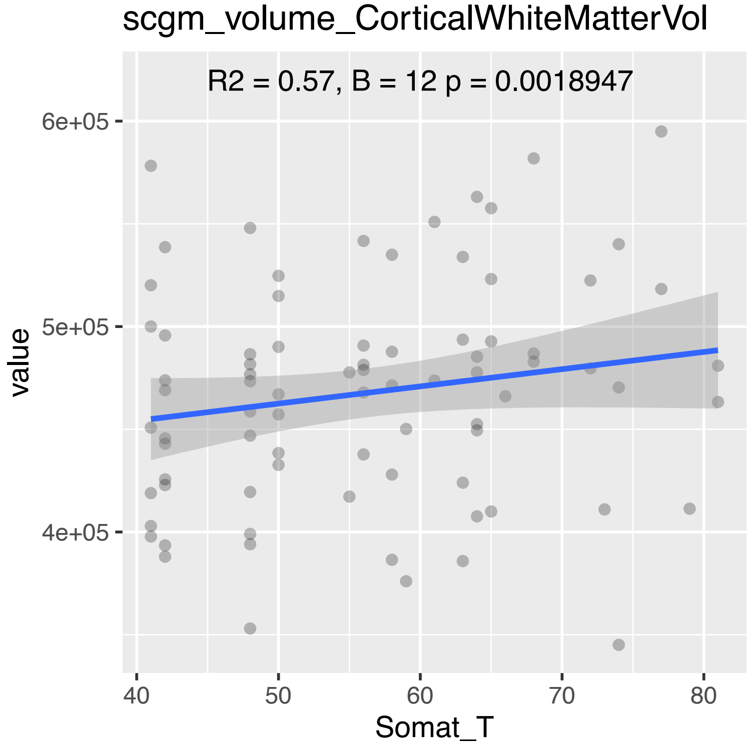
scgm_volume_CorticalWhiteMatterVol
R2 = 0.57, B = 12 p = 0.0018947
6e+05
5e+05
4e+05
value
40
50
60
70
80
Somat_T

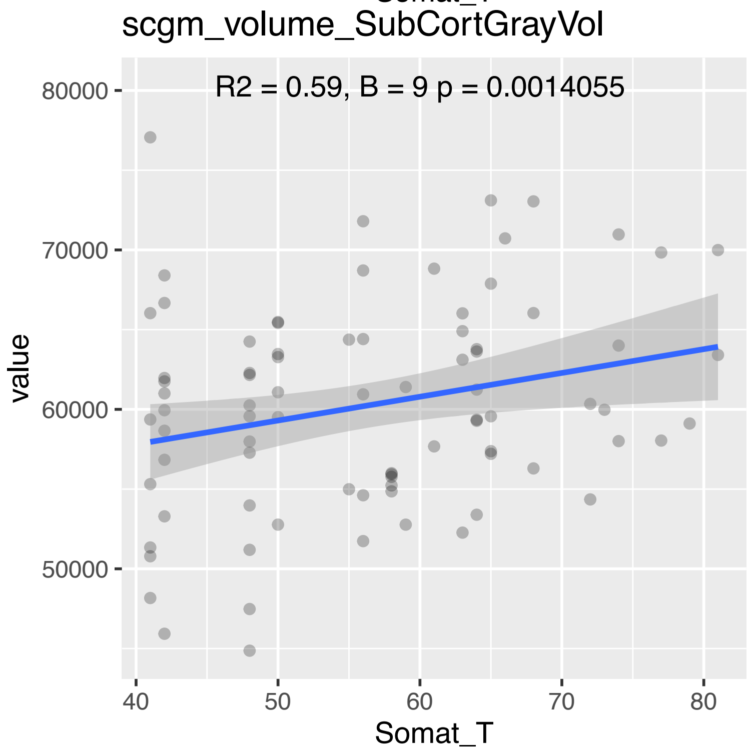
scgm_volume_SubCortGrayVol
R2 = 0.59, B = 9 p = 0.0014055
80000
70000
60000
50000
value
40
50
60
70
80
Somat_T

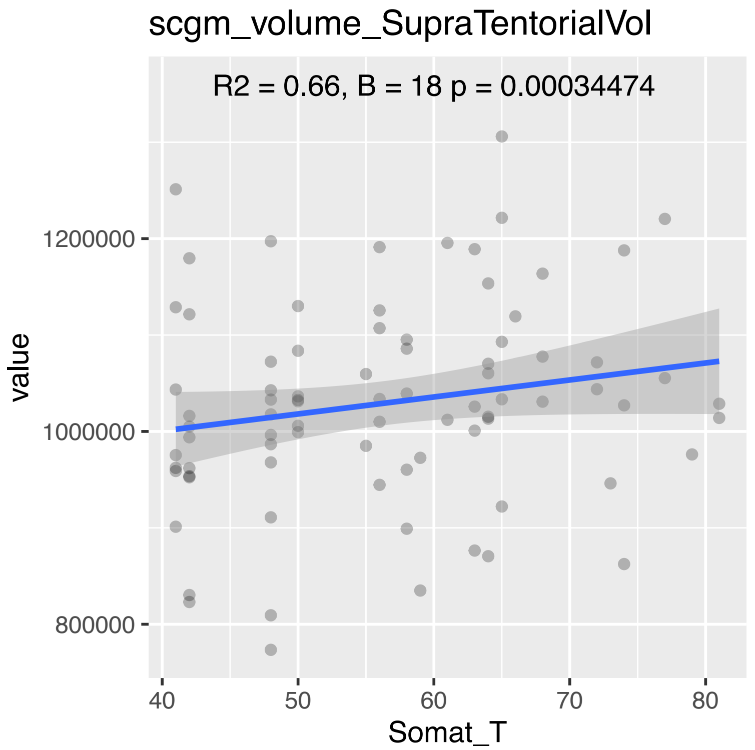
scgm_volume_SupraTentorialVol
R2 = 0.66, B = 18 p = 0.00034474
value
1200000
1000000
800000
40
50
60
70
80
Somat_T

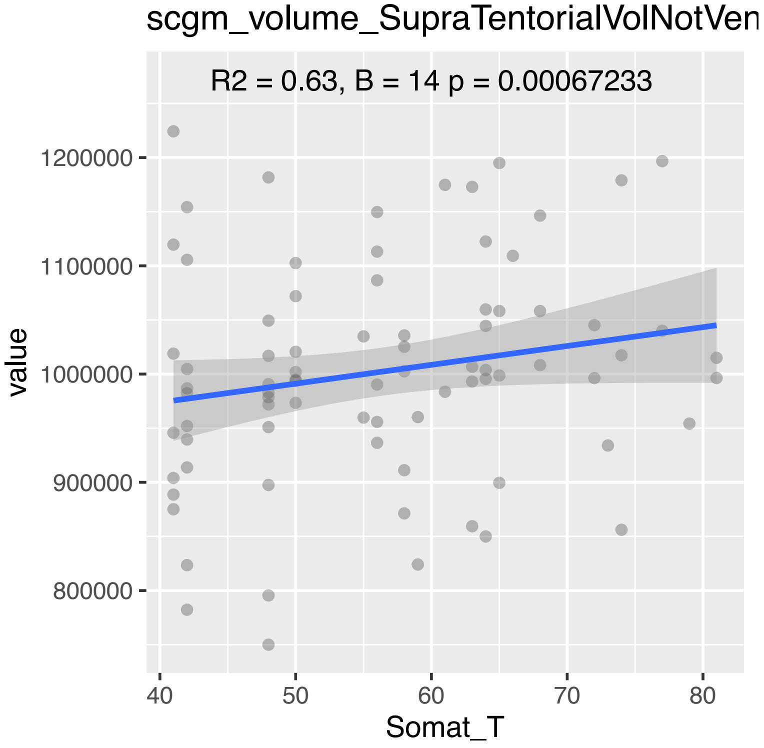
scgm_volume_SupraTentorialVolNotVen
R2 = 0.63, B = 14 p = 0.00067233
value
1200000
1100000
1000000
900000
800000
40
50
60
70
80
Somat_T

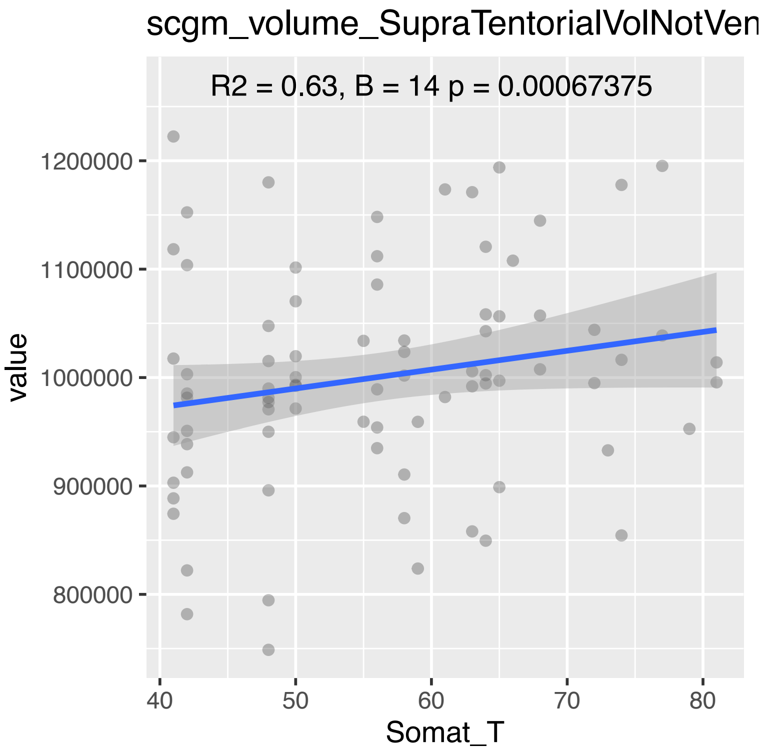
scgm_volume_SupraTentorialVolNotVen
R2 = 0.63, B = 14 p = 0.00067375
value
Somat_T

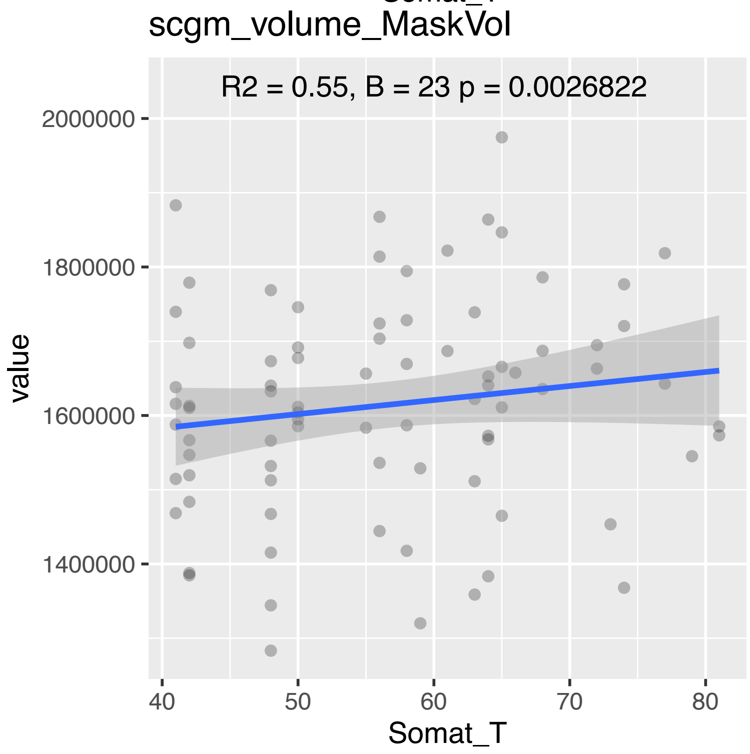
scgm_volume_MaskVol
R2 = 0.55, B = 23 p = 0.0026822
value
Somat_T

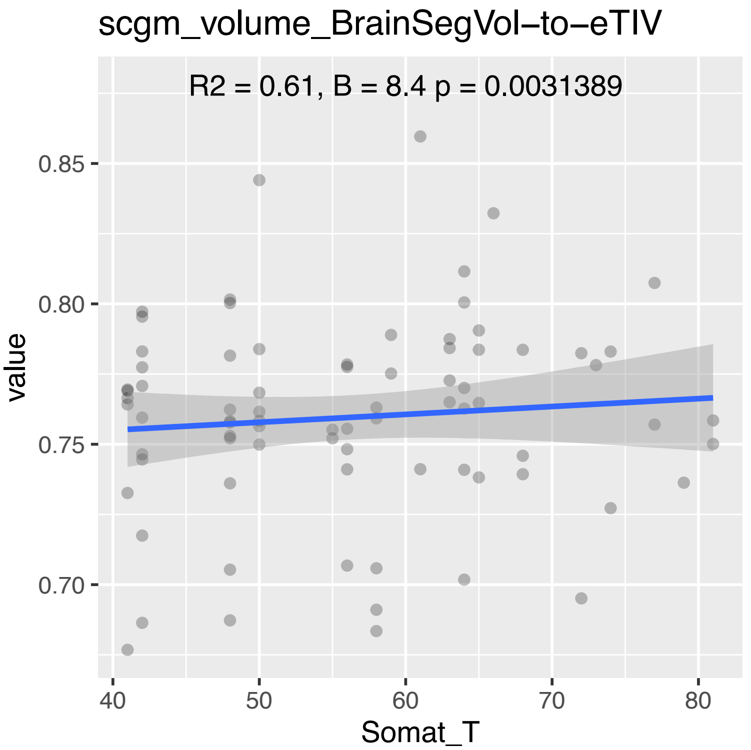
scgm_volume_BrainSegVol-to-eTIV
R2 = 0.61, B = 8.4 p = 0.0031389
0.85
0.80
0.75
0.70
value
40
50
60
70
80
Somat_T

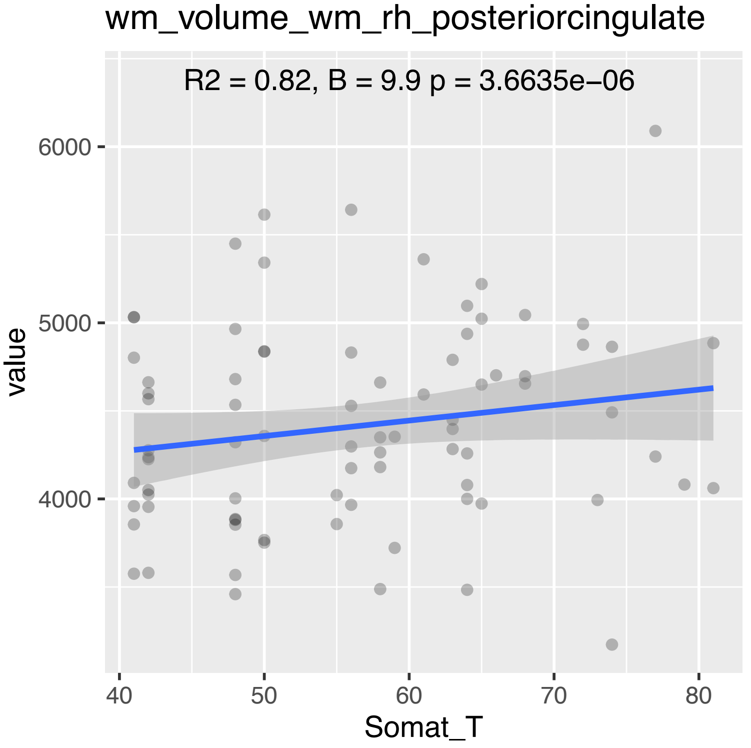
wm_volume_wm_rh_posteriorcingulate
R2 = 0.82, B = 9.9 p = 3.6635e-06
6000
5000
4000
value
40
50
60
70
80
Somat_T

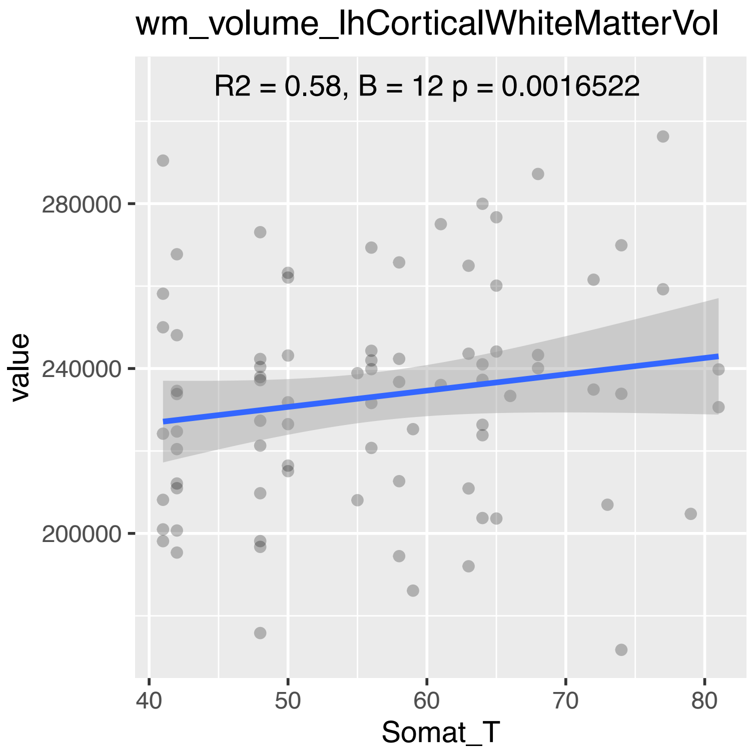
wm_volume_lhCorticalWhiteMatterVol
R2 = 0.58, B = 12 p = 0.0016522
280000
240000
200000
value
40
50
60
70
80
Somat_T

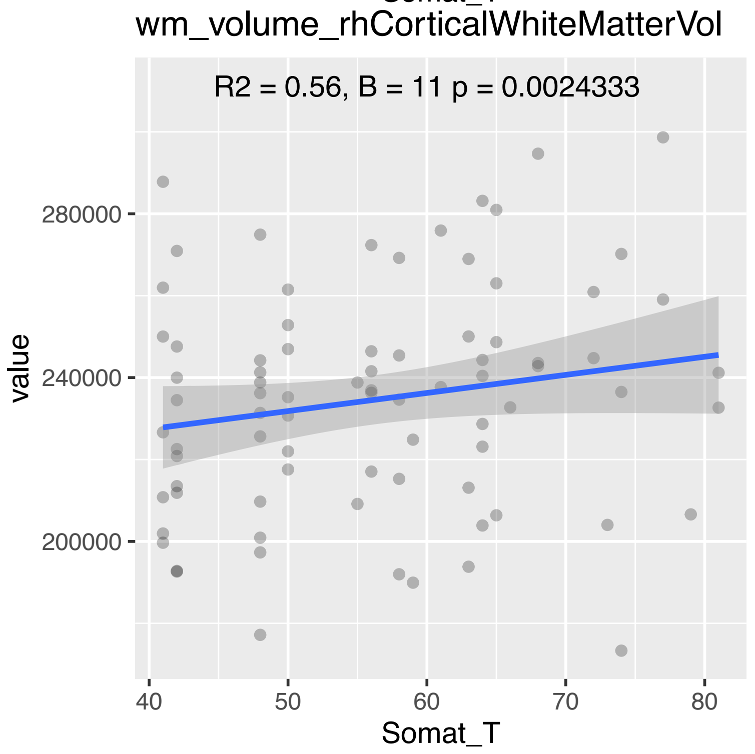
wm_volume_rhCorticalWhiteMatterVol
R2 = 0.56, B = 11 p = 0.0024333
280000
240000
200000
value
40
50
60
70
80
Somat_T

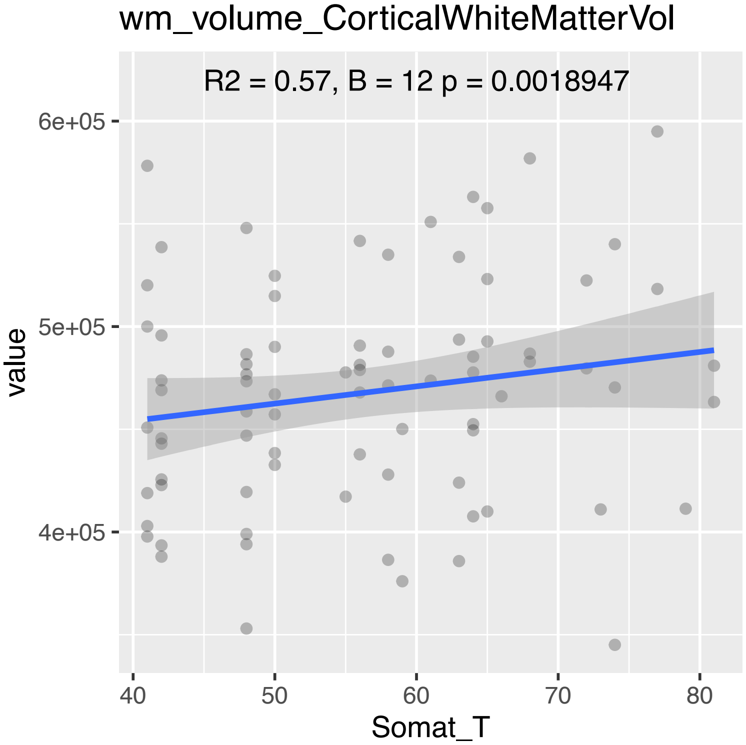
wm_volume_CorticalWhiteMatterVol
R2 = 0.57, B = 12 p = 0.0018947
6e+05
5e+05
4e+05
value
40
50
60
70
80
Somat_T

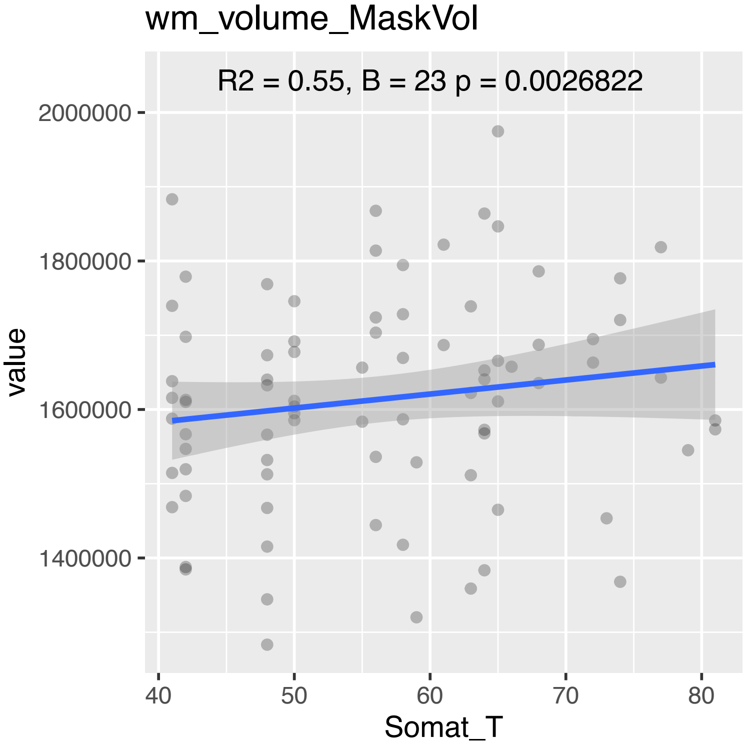
wm_volume_MaskVol
R2 = 0.55, B = 23 p = 0.0026822
2000000
1800000
1600000
1400000
value
40
50
60
70
80
Somat_T

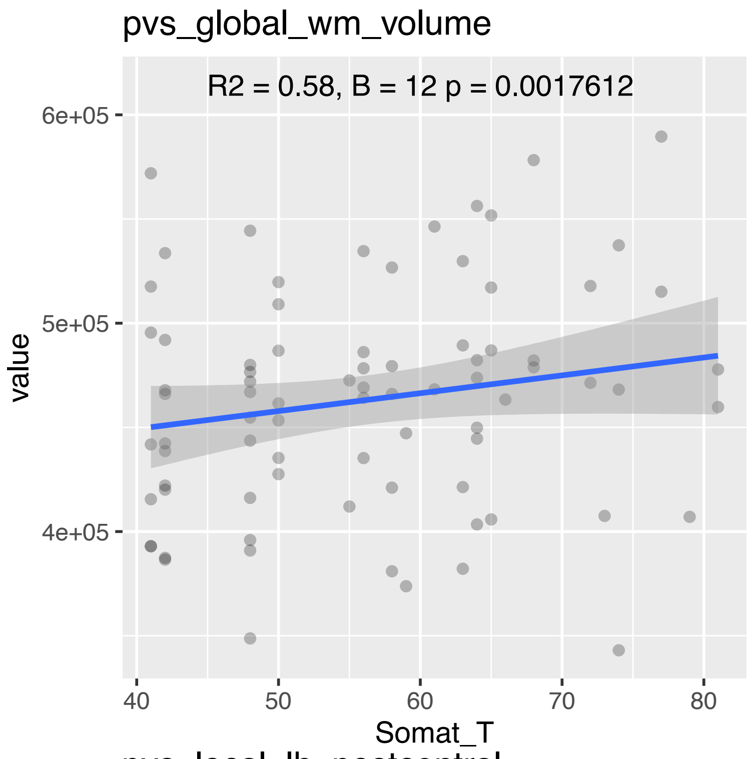
pvs_global_wm_volume
R2 = 0.58, B = 12 p = 0.0017612
6e+05
5e+05
4e+05
value
40
50
60
70
80
Somat_T

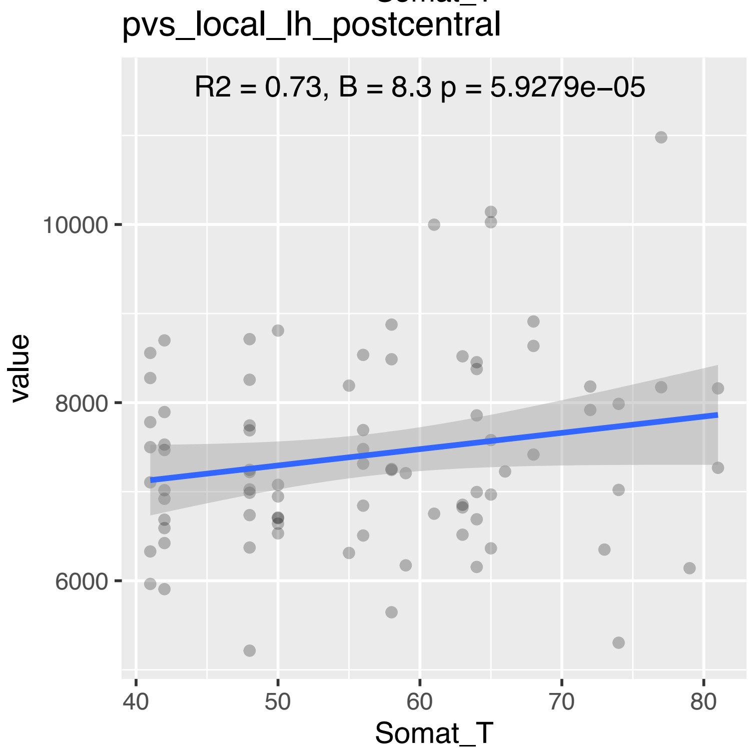
pvs_local_lh_postcentral
R2 = 0.73, B = 8.3 p = 5.9279e−05
10000
8000
6000
value
40
50
60
70
80
Somat_T

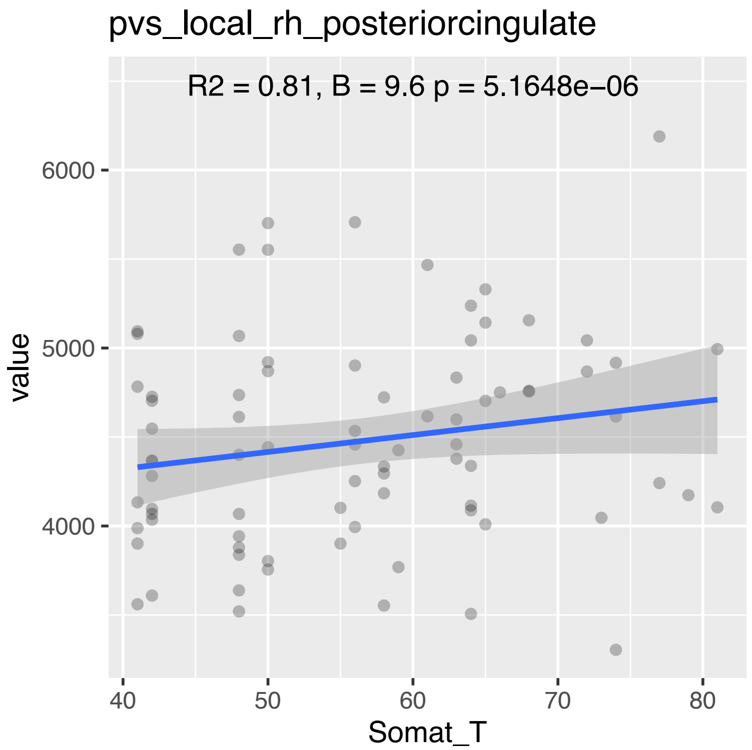
pvs_local_rh_posteriorcingulate
R2 = 0.81, B = 9.6 p = 5.1648e−06
6000
5000
4000
value
40
50
60
70
80
Somat_T

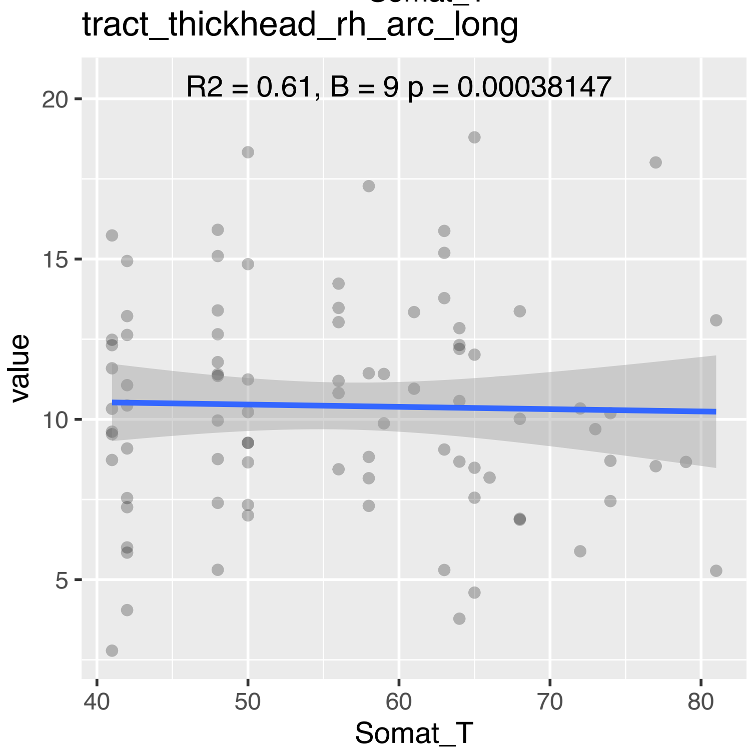
tract_thickhead_rh_arc_long
R2 = 0.61, B = 9 p = 0.00038147
20
15
10
5
value
40
50
60
70
80
Somat_T

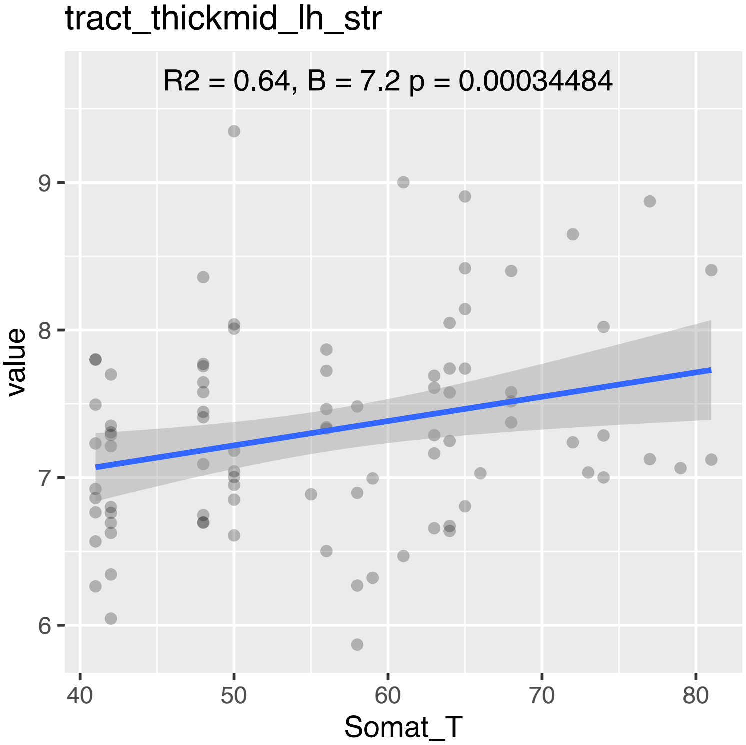

Figure S6: Scatterplots indicating the significant relationships between brain areas and somatization scores (here hemispheres are considered separately)

2.3. Life satisfactory

Uncorrected p-values showed significant relationships with diffusivity measures and tract geometrical features of the cingulum, SLF-I, fornix, uncinate and arcuate fasciculus. Associations were found with insular perivascular spaces and cortical thickness, but they didn't survive FDR correction.

Brain region	beta	p-val	improvebeta	improvepval	q-val
tract_length_cerebellar_sup	-3.2589278	0.00595542	0.004782674	0.949570986	0.16079621
jhu_fa_Superior_fronto-occipital_fasciculus	2.6315805	0.00677595	0.101130938	0.172375327	0.1829506
tract_ad_cerebellar_inf	2.92857412	0.00788333	0.130082526	0.126070951	0.21285003
ctx_thick_S_circular_insula_sup	-3.2639964	0.00993832	-0.040618702	0.629087768	0.56645679
tract_thickmid_unc	-2.907131	0.01016442	0.023033574	0.760883663	0.27443937
tract_md_cerebellar_inf	2.97982322	0.01379002	0.117938183	0.166367448	0.37233061
ctx_thick_S_temporal_sup	-3.8828774	0.01452453	-0.137287958	0.231619869	0.56645679
scgm_volume_Putamen	3.15676673	0.01547112	0.180319349	0.039310574	0.55700652
tract_num_cing_body	2.66612932	0.02152495	0.095757081	0.218613139	0.35369071
tract_fa_arc_post	2.91364685	0.02261512	0.137277056	0.108349065	0.28452171
tract_thicktail_arc_post	-2.4292016	0.02384095	0.033303405	0.670754402	0.64370559
tract_length_corona_ant	-4.1453338	0.02384844	0.137721677	0.09781633	0.2923092
tract_den_cing_body	2.50611015	0.02474612	0.097414658	0.213226607	0.63744612
tract_num_mid_cerebellar	4.19520781	0.02619931	0.050053089	0.68911258	0.35369071
tract_thickend_slfi	2.31382325	0.02638064	0.078180038	0.125067932	0.59680589
tract_fa_slfi	-2.8133649	0.02759834	-0.045894979	0.464269097	0.28452171
pvs_local_superiorfrontal	6.24937086	0.03119976	0.101754703	0.35534749	0.97481464
tract_thickhead_slfi	1.994169	0.03203223	0.080898681	0.120401784	0.86487008
scgm_volume_lhateral-Ventricle	-4.2655124	0.03352875	0.230276791	0.096849676	0.55700652
tract_mag_atr	2.29070498	0.03546998	0.030378227	0.676485934	0.48264618
tract_rd_slfi	3.17651897	0.03851476	-0.075227351	0.319847159	0.38952782
tract_length_fornix	3.15520524	0.04003661	0.240150635	0.040374973	0.2923092
scgm_volume_choroid-plexus	-2.4700572	0.04004692	0.093950291	0.220738102	0.55700652
tract_fa_arc_ant	2.2240613	0.04189553	0.057818781	0.436831752	0.28452171
tract_fa_ilf	2.15921447	0.04215136	0.118672847	0.147894445	0.28452171
tract_length_str	-3.2586526	0.04330507	0.153583842	0.084351231	0.2923092
scgm_fa_amygdala	2.34463001	0.04353469	0.094888765	0.226409313	0.2175578
tract_rd_cerebellar_sup	1.92781291	0.04376028	0.048343961	0.52878938	0.38952782
tract_thickend_arc_post	-2.5053613	0.04575646	0.007441113	0.92923542	0.59680589
scgm_volume_rhateral-Ventricle	-4.9696646	0.04641721	0.252510575	0.091061127	0.55700652
pvs_local_insula	-4.7042153	0.04748923	0.093615444	0.404944388	0.97481464

Table S4: Life satisfactory uncorrected p-values (hemispheres analyzed together)

Brain Region	beta	p-val	improvebeta	improvepval	q-val
tract_thickend_lh_arc_post	-5.3581662	0.00191152	-0.044001568	0.652286717	0.09366461
tract_thickhead_lh_fornix	3.98116562	0.00390835	0.085684846	0.415206481	0.19150919
scgm_fa_rh_hippocampus	4.87108281	0.00534329	0.173961116	0.10358201	0.07480609
tract_thicktail_lh_arc_post	-5.1060441	0.0114084	0.084898005	0.420085762	0.55901162
tract_fa_rh_arc_post	4.50148023	0.01364141	0.192289917	0.112370672	0.51588847
tract_mag_lh_atr	3.62427263	0.02018789	0.04457256	0.662633704	0.80938378
tract_length_rh_cerebellar_sup	-5.344197	0.02037677	-0.039315486	0.730503035	0.73367803
tract_thickmid_rh_cing_body	5.21900304	0.02079228	0.026365916	0.809930274	0.84400863
tract_fa_lh_fat	4.34097972	0.02204659	0.204721528	0.105894337	0.51588847
scgm_volume_rh_choroid-plexus	-4.7669942	0.0228515	0.183175675	0.125048477	0.70786245
tract_num_mid_cerebellar	4.19520781	0.02619931	0.050053089	0.68911258	0.87693613
tract_thicktail_rh_atr	-3.6258549	0.03098394	0.023910783	0.832105356	0.62522066
pvs_local_rh_superiorfrontal	6.24937086	0.03119976	0.101754703	0.35534749	0.98894239
wm_volume_wm_rh_superiorfrontal	6.43673064	0.03240064	0.092985811	0.396736647	0.96212422
scgm_volume_lh_Lateral-Ventricle	-4.2655124	0.03352875	0.230276791	0.096849676	0.70786245
tract_thickmid_lh_cst	-4.4209641	0.03444933	-0.099676167	0.461939164	0.84400863
tract_ad_lh_cerebellar_inf	4.30742767	0.03485696	0.124362294	0.376936664	0.90889421
tract_length_lh_fornix	5.40639502	0.03495305	0.427221394	0.054926237	0.73367803
scgm_volume_lh_Putamen	4.25158015	0.03633315	0.21180349	0.105627975	0.70786245
tract_num_rh_opticrad	3.33631706	0.03789296	0.079698207	0.530034889	0.87693613
tract_den_rh_cing_ven	-4.146431	0.03998771	0.100440014	0.372643776	0.75537109
tract_mag_lh_fat	4.50512411	0.04154846	0.178465581	0.161479056	0.80938378
tract_den_rh_cing_body	4.03647884	0.04197286	0.158863992	0.19834522	0.75537109
ctx_thick_lh_G_and_S_cingul-Ant	-7.5151563	0.04366729	-0.140371667	0.348659844	0.98923317
tract_thicktail_lh_opticrad	3.68986519	0.04562657	0.241150252	0.104806039	0.62522066
scgm_fa_rh_amygdala	4.26616603	0.04568265	0.124028578	0.284203213	0.21703499
tract_ad_rh_cst	-4.0382568	0.04568866	0.144970367	0.241374091	0.90889421
tract_fa_rh_arc_ant	3.79273623	0.04607692	0.073362819	0.507920282	0.51588847
scgm_volume_rh_Lateral-Ventricle	-4.9696646	0.04641721	0.252510575	0.091061127	0.70786245
tract_fa_lh_atr	3.44267595	0.04704308	0.00423252	0.968734802	0.51588847
tract_thickhead_lh_cerebellar_sup	-3.5104721	0.04732717	0.048651468	0.663941816	0.95487656
pvs_local_lh_insula	-4.7042153	0.04748923	0.093615444	0.404944388	0.98894239

Table S5: uncorrected p-values of associations between Life Satisfaction and brain regions, analyzing hemispheres separately

3. Imaging parameters used as dependent variables obtained using QIT

- John Hopkins University DTI FA atlas

jhu_fa_Middle_cerebellar_peduncle
jhu_fa_Pontine_crossing_tract
jhu_fa_Genu_of_corpus_callosum
jhu_fa_Body_of_corpus_callosum
jhu_fa_Splenium_of_corpus_callosum
jhu_fa_Fornix_(column_and_body_of_fornix)
jhu_fa_Corticospinal_tract
jhu_fa_Medial_lemniscus
jhu_fa_Inferior_cerebellar_peduncle
jhu_fa_Superior_cerebellar_peduncle
jhu_fa_Cerebral_peduncle
jhu_fa_Anterior_limb_of_internal_capsule
jhu_fa_Posterior_limb_of_internal_capsule
jhu_fa_Retrolenticular_part_of_internal_capsule
jhu_fa_Anterior_corona_radiata
jhu_fa_Superior_corona_radiata
jhu_fa_Posterior_corona_radiata
jhu_fa_Posterior_thalamic_radiation_(include_optic_radiation)
jhu_fa_Sagittal_stratum_(include_inferior_longitidinal_fasciculus_and_inferior_fronto-occipital_fasciculus)
jhu_fa_External_capsule
jhu_fa_Cingulum_(cingulate_gyrus)
jhu_fa_Cingulum_(hippocampus)
jhu_fa_Fornix_(cres)_Stria_terminalis
jhu_fa_Superior_longitudinal_fasciculus
jhu_fa_Superior_fronto-occipital_fasciculus
jhu_fa_Uncinate_fasciculus
jhu_fa_Tapetum

- Subcortical grey matter atlas

scgm_fa_thalamus_proper
scgm_fa_caudate
scgm_fa_putamen
scgm_fa_pallidum
scgm_fa_hippocampus
scgm_fa_amygdala
scgm_fa_accumbens_area
scgm_volume_lateral-Ventricle
scgm_volume_Inf-Lat-Ventricle
scgm_volume_Cerebellum-White-Matter
scgm_volume_Cerebellum-Cortex
scgm_volume_Thalamus-Proper

scgm_volume_Caudate
scgm_volume_Putamen
scgm_volume_Pallidum
scgm_volume_3rd-Ventricle
scgm_volume_4th-Ventricle
scgm_volume_Brainstem
scgm_volume_Hippocampus
scgm_volume_Amygdala
scgm_volume_CSF
scgm_volume_Accumbens-area
scgm_volume_VentralDC
scgm_volume_vessel
scgm_volume_choroid-plexus
scgm_volume_rh_lateral-Ventricle
scgm_volume_wm_hypointensities
scgm_volume_non-wm_hypointensities
scgm_volume_Optic-Chiasm
scgm_volume_CC_Posterior
scgm_volume_CC_Mid_Posterior
scgm_volume_CC_Central
scgm_volume_CC_Mid_Anterior
scgm_volume_CC_Anterior
scgm_volume_BrainSegVol
scgm_volume_BrainSegVolNotVent
scgm_volume_BrainSegVolNotVentSurf
scgm_volume_lhCortexVol
scgm_volume_rhCortexVol
scgm_volume_CortexVol
scgm_volume_lhCorticalWhiteMatterVol
scgm_volume_rhCorticalWhiteMatterVol
scgm_volume_CorticalWhiteMatterVol
scgm_volume_SubCortGrayVol
scgm_volume_TotalGrayVol
scgm_volume_SupraTentorialVol
scgm_volume_SupraTentorialVolNotVent
scgm_volume_SupraTentorialVolNotVentVox
scgm_volume_MaskVol
scgm_volume_BrainSegVol-to-eTIV
scgm_volume_MaskVol-to-eTIV
scgm_volume_lhSurfaceHoles
scgm_volume_rhSurfaceHoles
scgm_volume_SurfaceHoles

- White matter regional volume atlas

wm_volume_wm_bankssts

wm_volume_wm_caudalanteriorcingulate
wm_volume_wm_caudalmiddlefrontal
wm_volume_wm_cuneus
wm_volume_wm_entorhinal
wm_volume_wm_fusiform
wm_volume_wm_inferiorparietal
wm_volume_wm_inferiortemporal
wm_volume_wm_isthmuscingulate
wm_volume_wm_lateraloccipital
wm_volume_wm_lateralorbitofrontal
wm_volume_wm_lingual
wm_volume_wm_medialorbitofrontal
wm_volume_wm_middletemporal
wm_volume_wm_parahippocampal
wm_volume_wm_paracentral
wm_volume_wm_parsopercularis
wm_volume_wm_parsorbitalis
wm_volume_wm_parstriangularis
wm_volume_wm_pericalcarine
wm_volume_wm_postcentral
wm_volume_wm_posteriorcingulate
wm_volume_wm_precentral
wm_volume_wm_precuneus
wm_volume_wm_rostralanteriorcingulate
wm_volume_wm_rostralmiddlefrontal
wm_volume_wm_superiorfrontal
wm_volume_wm_superiorparietal
wm_volume_wm_superiortemporal
wm_volume_wm_supramarginal
wm_volume_wm_frontalpole
wm_volume_wm_temporalpole
wm_volume_wm_transversetemporal
wm_volume_wm_insula
wm_volume_UnsegmentedWhiteMatter
wm_volume_lhCorticalWhiteMatterVol
wm_volume_rhCorticalWhiteMatterVol
wm_volume_CorticalWhiteMatterVol
wm_volume_MaskVol

- Cortical thickness GM regional atlas (‘G’ stands for gyrus and ‘S’ stands for sulcus)

ctx_thick_G_and_S_frontomargin
ctx_thick_G_and_S_occipital_inf
ctx_thick_G_and_S_paracentral
ctx_thick_G_and_S_subcentral

ctx_thick_G_and_S_transv_frontopol
ctx_thick_G_and_S_cingul-Ant
ctx_thick_G_and_S_cingul-Mid-Ant
ctx_thick_G_and_S_cingul-Mid-Post
ctx_thick_G_cingul-Post-dorsal
ctx_thick_G_cingul-Post-ventral
ctx_thick_G_cuneus
ctx_thick_G_front_inf-Opercular
ctx_thick_G_front_inf-Orbital
ctx_thick_G_front_inf-Triangul
ctx_thick_G_front_middle
ctx_thick_G_front_sup
ctx_thick_G_Ins_lg_and_S_cent_ins
ctx_thick_G_insular_short
ctx_thick_G_occipital_middle
ctx_thick_G_occipital_sup
ctx_thick_G_oc-temp_lat-fusifor
ctx_thick_G_oc-temp_med-Lingual
ctx_thick_G_oc-temp_med-Parahip
ctx_thick_G_orbital
ctx_thick_G_pariet_inf-Angular
ctx_thick_G_pariet_inf-Supramar
ctx_thick_G_parietal_sup
ctx_thick_G_postcentral
ctx_thick_G_precentral
ctx_thick_G_precuneus
ctx_thick_G_rectus
ctx_thick_G_subcallosal
ctx_thick_G_temp_sup-G_T_transv
ctx_thick_G_temp_sup-Lateral
ctx_thick_G_temp_sup-Plan_polar
ctx_thick_G_temp_sup-Plan_tempo
ctx_thick_G_temporal_inf
ctx_thick_G_temporal_middle
ctx_thick_Lat_Fis-ant-Horizont
ctx_thick_Lat_Fis-ant-Vertical
ctx_thick_Lat_Fis-post
ctx_thick_Pole_occipital
ctx_thick_Pole_temporal
ctx_thick_S_calcarine
ctx_thick_S_central
ctx_thick_S_cingul-Marginalis
ctx_thick_S_circular_insula_ant
ctx_thick_S_circular_insula_inf
ctx_thick_S_circular_insula_sup
ctx_thick_S_collat_transv_ant

ctx_thick_S_collat_transv_post
ctx_thick_S_front_inf
ctx_thick_S_front_middle
ctx_thick_S_front_sup
ctx_thick_S_interm_prim-Jensen
ctx_thick_S_intrapariet_and_P_trans
ctx_thick_S_oc_middle_andunatus
ctx_thick_S_oc_sup_and_transversal
ctx_thick_S_occipital_ant
ctx_thick_S_oc-temp_lat
ctx_thick_S_oc-temp_med_andingual
ctx_thick_S_orbital_lateral
ctx_thick_S_orbital_med-olfact
ctx_thick_S_orbital-H_Shaped
ctx_thick_S_parieto_occipital
ctx_thick_S_pericallosal
ctx_thick_S_postcentral
ctx_thick_S_precentral-inf-part
ctx_thick_S_precentral-sup-part
ctx_thick_S_suborbital
ctx_thick_S_subparietal
ctx_thick_S_temporal_inf
ctx_thick_S_temporal_sup
ctx_thick_S_temporal_transverse
ctx_thick_MeanThickness
ctx_thick_rhat_Fis-ant-Horizont
ctx_thick_rhat_Fis-ant-Vertical
ctx_thick_rhat_Fis-post

- PVS global and regional atlas

pvs_global_wm_pvs_volume
pvs_global_wm_volume
pvs_local_bankssts
pvs_local_caudalanteriorcingulate
pvs_local_caudalmiddlefrontal
pvs_local_cuneus
pvs_local_entorhinal
pvs_local_fusiform
pvs_local_inferiorparietal
pvs_local_inferiortemporal
pvs_local_isthmuscingulate
pvs_local_lateraloccipital
pvs_local_lateralorbitofrontal
pvs_local_lingual
pvs_local_medialorbitofrontal

pvs_local_middletemporal
pvs_local_parahippocampal
pvs_local_paracentral
pvs_local_parsopercularis
pvs_local_parsorbitalis
pvs_local_parstriangularis
pvs_local_pericalcarine
pvs_local_postcentral
pvs_local_posteriorcingulate
pvs_local_precentral
pvs_local_precuneus
pvs_local_rostralanteriorcingulate
pvs_local_rostralmiddlefrontal
pvs_local_superiorfrontal
pvs_local_superiorparietal
pvs_local_superiortemporal
pvs_local_supramarginal
pvs_local_frontalpole
pvs_local_temporalpole
pvs_local_transversetemporal
pvs_local_insula
pvs_local_inferiorparietal
pvs_local_inferiortemporal
pvs_local_isthmuscingulate
pvs_local_lateraloccipital
pvs_local_lateralorbitofrontal
pvs_local_lingual
pvs_local_medialorbitofrontal
pvs_local_middletemporal
pvs_local_parahippocampal
pvs_local_paracentral
pvs_local_parsopercularis
pvs_local_parsorbitalis
pvs_local_parstriangularis
pvs_local_pericalcarine
pvs_local_postcentral
pvs_local_posteriorcingulate
pvs_local_precentral
pvs_local_precuneus
pvs_local_rostralmiddlefrontal
pvs_local_superiorfrontal
pvs_local_superiorparietal
pvs_local_superiortemporal
pvs_local_supramarginal
pvs_local_frontalpole
pvs_local_temporalpole

pvs_local_transversetemporal
pvs_local_insula
pvs_local_Left-UnsegmentedWhiteMatter
pvs_local_Right-UnsegmentedWhiteMatter

- DTI tract-based diffusivity measures (FA, MD, RD, AD) and tract properties (density, length, magnitude, number, volume and thickness)

tract_den_arc_ant
tract_den_opticrad
tract_den_unc
tract_den_ifof
tract_den_cst
tract_den_arc_post
tract_den_fornix
tract_den_cerebellar_sup
tract_den_slfii
tract_den_cing_ven
tract_den_corona_ant
tract_den_vof
tract_den_slfiii
tract_den_cing_body
tract_den_arc_long
tract_den_cerebellar_inf
tract_den_str
tract_den_cing_genu
tract_den_mid_cerebellar
tract_den_ilf
tract_den_forceps_minor
tract_den_mlf
tract_den_forceps_major
tract_den_corona_post
tract_den_fat
tract_den_atr
tract_den_slfi
tract_length_arc_ant
tract_length_opticrad
tract_length_unc
tract_length_ifof
tract_length_cst
tract_length_arc_post
tract_length_fornix
tract_length_cerebellar_sup
tract_length_slfii
tract_length_cing_ven
tract_length_corona_ant
tract_length_vof

tract_length_slfiii
tract_length_cing_body
tract_length_arc_long
tract_length_cerebellar_inf
tract_length_str
tract_length_cing_genu
tract_length_mid_cerebellar
tract_length_ilf
tract_length_forceps_minor
tract_length_mlf
tract_length_forceps_major
tract_length_corona_post
tract_length_fat
tract_length_atr
tract_length_slfi
tract_mag_arc_ant
tract_mag_opticrad
tract_mag_unc
tract_mag_ifof
tract_mag_cst
tract_mag_arc_post
tract_mag_fornix
tract_mag_cerebellar_sup
tract_mag_slfii
tract_mag_cing_ven
tract_mag_corona_ant
tract_mag_vof
tract_mag_slfiii
tract_mag_cing_body
tract_mag_arc_long
tract_mag_cerebellar_inf
tract_mag_str
tract_mag_cing_genu
tract_mag_mid_cerebellar
tract_mag_ilf
tract_mag_forceps_minor
tract_mag_mlf
tract_mag_forceps_major
tract_mag_corona_post
tract_mag_fat
tract_mag_atr
tract_mag_slfi
tract_num_arc_ant
tract_num_opticrad
tract_num_unc
tract_num_ifof

tract_num_cst
tract_num_arc_post
tract_num_fornix
tract_num_cerebellar_sup
tract_num_slfii
tract_num_cing_ven
tract_num_corona_ant
tract_num_vof
tract_num_slfiii
tract_num_cing_body
tract_num_arc_long
tract_num_cerebellar_inf
tract_num_str
tract_num_cing_genu
tract_num_mid_cerebellar
tract_num_ilf
tract_num_forceps_minor
tract_num_mlf
tract_num_forceps_major
tract_num_corona_post
tract_num_fat
tract_num_atr
tract_num_slfi
tract_thickend_arc_ant
tract_thickend_opticrad
tract_thickend_unc
tract_thickend_ifof
tract_thickend_cst
tract_thickend_arc_post
tract_thickend_fornix
tract_thickend_cerebellar_sup
tract_thickend_slfii
tract_thickend_cing_ven
tract_thickend_corona_ant
tract_thickend_vof
tract_thickend_slfiii
tract_thickend_cing_body
tract_thickend_arc_long
tract_thickend_cerebellar_inf
tract_thickend_str
tract_thickend_cing_genu
tract_thickend_mid_cerebellar
tract_thickend_ilf
tract_thickend_forceps_minor
tract_thickend_mlf
tract_thickend_forceps_major

tract_thickend_corona_post
tract_thickend_fat
tract_thickend_atr
tract_thickend_slfi
tract_thickhead_arc_ant
tract_thickhead_opticrad
tract_thickhead_unc
tract_thickhead_ifof
tract_thickhead_cst
tract_thickhead_arc_post
tract_thickhead_fornix
tract_thickhead_cerebellar_sup
tract_thickhead_slfii
tract_thickhead_cing_ven
tract_thickhead_corona_ant
tract_thickhead_vof
tract_thickhead_slfiii
tract_thickhead_cing_body
tract_thickhead_arc_long
tract_thickhead_cerebellar_inf
tract_thickhead_str
tract_thickhead_cing_genu
tract_thickhead_mid_cerebellar
tract_thickhead_ilf
tract_thickhead_forceps_minor
tract_thickhead_mlf
tract_thickhead_forceps_major
tract_thickhead_corona_post
tract_thickhead_fat
tract_thickhead_atr
tract_thickhead_slfi
tract_thickmid_arc_ant
tract_thickmid_opticrad
tract_thickmid_unc
tract_thickmid_ifof
tract_thickmid_cst
tract_thickmid_arc_post
tract_thickmid_fornix
tract_thickmid_cerebellar_sup
tract_thickmid_slfii
tract_thickmid_cing_ven
tract_thickmid_corona_ant
tract_thickmid_vof
tract_thickmid_slfiii
tract_thickmid_cing_body
tract_thickmid_arc_long

tract_thickmid_cerebellar_inf
tract_thickmid_str
tract_thickmid_cing_genu
tract_thickmid_mid_cerebellar
tract_thickmid_ilf
tract_thickmid_forceps_minor
tract_thickmid_mlf
tract_thickmid_forceps_major
tract_thickmid_corona_post
tract_thickmid_fat
tract_thickmid_atr
tract_thickmid_slfi
tract_thicktail_arc_ant
tract_thicktail_opticrad
tract_thicktail_unc
tract_thicktail_ifof
tract_thicktail_cst
tract_thicktail_arc_post
tract_thicktail_fornix
tract_thicktail_cerebellar_sup
tract_thicktail_slfii
tract_thicktail_cing_ven
tract_thicktail_corona_ant
tract_thicktail_vof
tract_thicktail_slfiii
tract_thicktail_cing_body
tract_thicktail_arc_long
tract_thicktail_cerebellar_inf
tract_thicktail_str
tract_thicktail_cing_genu
tract_thicktail_mid_cerebellar
tract_thicktail_ilf
tract_thicktail_forceps_minor
tract_thicktail_mlf
tract_thicktail_forceps_major
tract_thicktail_corona_post
tract_thicktail_fat
tract_thicktail_atr
tract_thicktail_slfi
tract_volume_arc_ant
tract_volume_opticrad
tract_volume_unc
tract_volume_ifof
tract_volume_cst
tract_volume_arc_post
tract_volume_fornix

tract_volume_cerebellar_sup
tract_volume_slfii
tract_volume_cing_ven
tract_volume_corona_ant
tract_volume_vof
tract_volume_slfiii
tract_volume_cing_body
tract_volume_arc_long
tract_volume_cerebellar_inf
tract_volume_str
tract_volume_cing_genu
tract_volume_mid_cerebellar
tract_volume_ilf
tract_volume_forceps_minor
tract_volume_mlf
tract_volume_forceps_major
tract_volume_corona_post
tract_volume_fat
tract_volume_atr
tract_volume_slfi
tract_ad_arc_ant
tract_ad_opticrad
tract_ad_unc
tract_ad_ifof
tract_ad_cst
tract_ad_arc_post
tract_ad_fornix
tract_ad_cerebellar_sup
tract_ad_slfii
tract_ad_cing_ven
tract_ad_corona_ant
tract_ad_vof
tract_ad_slfiii
tract_ad_cing_body
tract_ad_arc_long
tract_ad_cerebellar_inf
tract_ad_str
tract_ad_cing_genu
tract_ad_mid_cerebellar
tract_ad_ilf
tract_ad_forceps_minor
tract_ad_mlf
tract_ad_forceps_major
tract_ad_corona_post
tract_ad_fat
tract_ad_atr

tract_ad_slfi
tract_fa_arc_ant
tract_fa_opticrad
tract_fa_unc
tract_fa_ifof
tract_fa_cst
tract_fa_arc_post
tract_fa_fornix
tract_fa_cerebellar_sup
tract_fa_slfii
tract_fa_cing_ven
tract_fa_corona_ant
tract_fa_vof
tract_fa_slfiii
tract_fa_cing_body
tract_fa_arc_long
tract_fa_cerebellar_inf
tract_fa_str
tract_fa_cing_genu
tract_fa_mid_cerebellar
tract_fa_ilf
tract_fa_forceps_minor
tract_fa_mlf
tract_fa_forceps_major
tract_fa_corona_post
tract_fa_fat
tract_fa_atr
tract_fa_slfi
tract_md_arc_ant
tract_md_opticrad
tract_md_unc
tract_md_ifof
tract_md_cst
tract_md_arc_post
tract_md_fornix
tract_md_cerebellar_sup
tract_md_slfii
tract_md_cing_ven
tract_md_corona_ant
tract_md_vof
tract_md_slfiii
tract_md_cing_body
tract_md_arc_long
tract_md_cerebellar_inf
tract_md_str
tract_md_cing_genu

tract_md_mid_cerebellar
tract_md_ilf
tract_md_forceps_minor
tract_md_mlf
tract_md_forceps_major
tract_md_corona_post
tract_md_fat
tract_md_atr
tract_md_slfi
tract_rd_arc_ant
tract_rd_opticrad
tract_rd_unc
tract_rd_ifof
tract_rd_cst
tract_rd_arc_post
tract_rd_fornix
tract_rd_cerebellar_sup
tract_rd_slfii
tract_rd_cing_ven
tract_rd_corona_ant
tract_rd_vof
tract_rd_slfiii
tract_rd_cing_body
tract_rd_arc_long
tract_rd_cerebellar_inf
tract_rd_str
tract_rd_cing_genu
tract_rd_mid_cerebellar
tract_rd_ilf
tract_rd_forceps_minor
tract_rd_mlf
tract_rd_forceps_major
tract_rd_corona_post
tract_rd_fat
tract_rd_atr
tract_rd_slfi

Table S6: List of the variables used for the statistical analysis. A regression model was run on each variable with an alpha level = 0.05 for each symptom separately. An FDR correction for multiple comparisons based on Benjamini-Hochberg approach was run on the variables (i.e. tests) that were statistically significant at the alpha level.
